# Catalytic aza-Nazarov cyclization reactions to access α-methylene-γ-lactam heterocycles

**DOI:** 10.3762/bjoc.19.6

**Published:** 2023-01-17

**Authors:** Bilge Banu Yagci, Selin Ezgi Donmez, Onur Şahin, Yunus Emre Türkmen

**Affiliations:** 1 Department of Chemistry, Faculty of Science, Bilkent University, Ankara 06800, Turkey; 2 Department of Occupational Health & Safety, Faculty of Health Sciences, Sinop University, Sinop 57000, Turkey; 3 UNAM – National Nanotechnology Research Center, Institute of Materials Science and Nanotechnology, Bilkent University, Ankara 06800, Turkey

**Keywords:** α-methylene-γ-lactam, aza-Nazarov reaction, β-silicon effect, heterocycles, intramolecular cyclization

## Abstract

We have developed a catalytic aza-Nazarov reaction of *N*-acyliminium salts generated in situ from the reaction of a variety of cyclic and acyclic imines with α,β-unsaturated acyl chlorides to afford substituted α-methylene-γ-lactam heterocycles. The reactions proceed effectively in the presence of catalytic (20 mol %) amounts of AgOTf as an anion exchange agent or hydrogen-bond donors such as squaramides and thioureas as anion-binding organocatalysts. The aza-Nazarov cyclization of 3,4-dihydroisoquinolines with α,β-unsaturated acyl chlorides gives tricyclic lactam products **7** in up to 79% yield with full diastereocontrol (dr = >99:1). The use of acyclic imines in a similar catalytic aza-Nazarov reaction with 20 mol % of AgOTf results in the formation of α-methylene-γ-lactam heterocycles **19** in up to 76% yield and with good to high diastereoselectivities (4.3:1 to 16:1). We have demonstrated the scalability of the reaction with a gram-scale example. The relative stereochemistry of the α-methylene-γ-lactam products **19** has been determined via the single-crystal X-ray analysis of lactam **19l**. In order to shed light on the details of the reaction mechanism, we have performed carefully designed mechanistic studies which consist of experiments on the effect of β-silicon stabilization, the alkene geometry of the α,β-unsaturated acyl chloride reactants, and adventitious water on the success of the catalytic aza-Nazarov reaction.

## Introduction

The rapid construction of aliphatic heterocycles from acyclic building blocks via cyclization or cycloaddition reactions constitutes one of the main pillars of organic synthesis [1]. In this respect, the all-carbon Nazarov cyclization of divinyl ketones represents a direct method for the synthesis of five-membered carbocycles [2–9]. Variants of the Nazarov cyclization with substrates bearing one or more heteroatoms in the dienone core have the potential to provide efficient access to a variety of heterocyclic scaffolds. Among such variants, the aza-Nazarov reaction appears as a highly suitable cyclization reaction that presents opportunities for the construction of a diverse array of five-membered nitrogen-heterocycles [10].

**Scheme 1:**
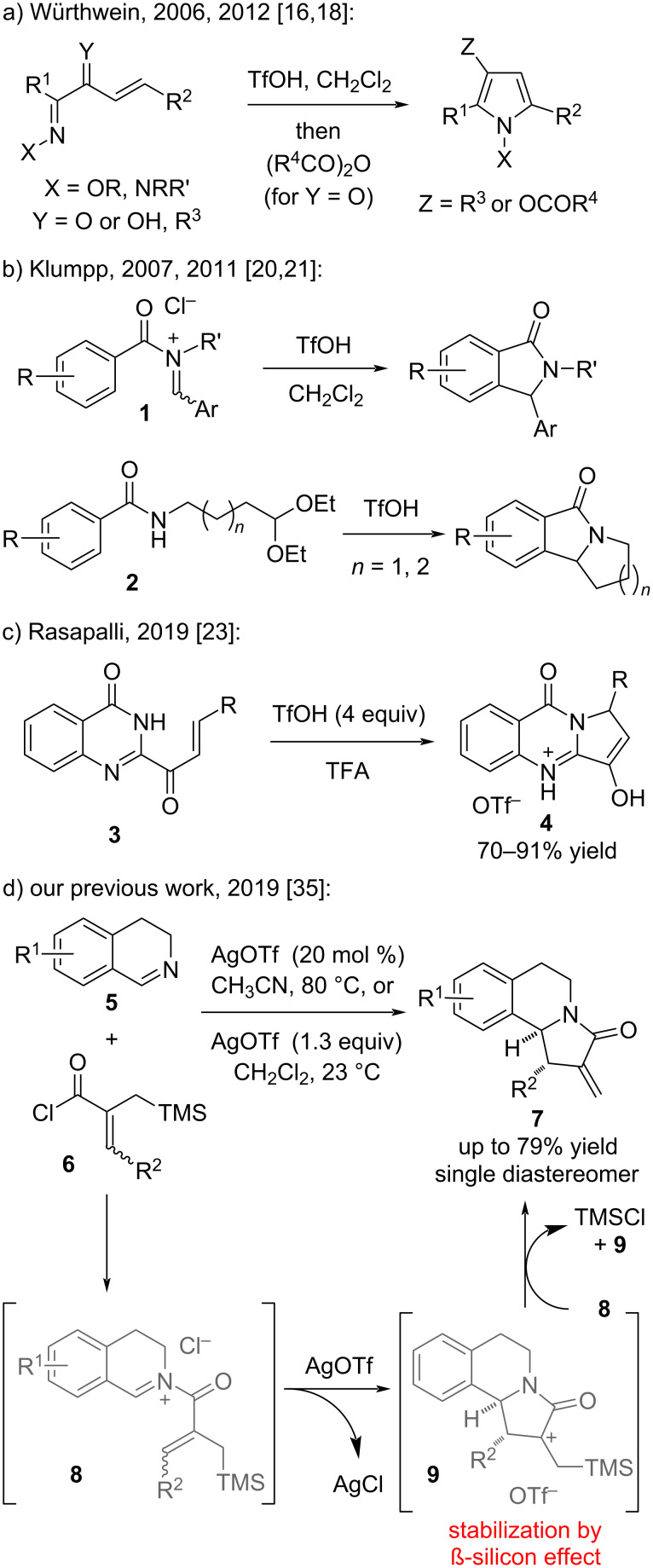
Examples of aza-Nazarov reactions.

Whereas an aza-Nazarov-type cyclization may be operative in some earlier examples [11–15], general interest in this area increased after the elegant studies of Würthwein and co-workers where they utilized the aza-Nazarov cyclization for the construction of multisubstituted pyrrole derivatives (Scheme 1a) [16–19]. Another seminal work in this field was the aza-Nazarov cyclization of *N*-acyliminium salts **1** reported by Klumpp and co-workers in 2007 (Scheme 1b) [20]. This reaction was promoted with the use of superstoichiometric amounts of TfOH (trifluoromethanesulfonic acid), and the *N*-acyliminium cation was proposed to be protonated with the super acidic TfOH to form a dicationic species, which was shown by computational studies to be crucial for the success of this transformation. In a later study, the same research group showed that benzamides **2** bearing an acetal group could be used as substrates in an aza-Nazarov cyclization with the intermediacy of in situ-generated *N*-acyliminium ions (Scheme 1b) [21]. The first catalytic aza-Nazarov reaction was reported by Tius and co-workers in 2010, which involved the kinetic resolution of azirine derivatives via an enantioselective organocatalytic aza-Nazarov cyclization affording six-membered heterocycles after a ring expansion of the cyclization products [22]. Rasapalli and co-workers recently developed an efficient aza-Nazarov cyclization of quinazolinonyl enones promoted by TfOH or MsOH (methanesulfonic acid) for the syntheses of C-aryl luotonins and vasicinones (Scheme 1c) [23–24]. Moreover, an aza-Nazarov cyclization was utilized for the construction of a variety of heterocyclic frameworks such as aminopyrroles [25–26], *N*-hydroxyoxindoles [27], indolizines [28], pyrido[1,2-*a*]indoles [29–30], indoles [31–32], and pyrrole-fused heterocyclic tricycles [33]. The involvement of Nazarov and in particular aza-Nazarov reactions in the cyclization of alkynes that go through metal carbene intermediates has recently been reviewed by Gao and co-workers [34].

In 2019, we reported a highly effective aza-Nazarov cyclization for the synthesis of tricyclic α-methylene-γ-lactams **7** as single diastereomers and in good to high yields through the use of a catalytic amount of AgOTf (silver trifluoromethanesulfonate) as an anion-exchange agent (Scheme 1d) [35]. In this transformation, treatment of 3,4-dihydroisoquinolines **5** with acyl chlorides **6** initially gives *N*-acyliminium salts **8**, which are activated upon treatment with AgOTf resulting in an anion metathesis of Cl^−^ with OTf^−^. This activation is proposed to facilitate the desired aza-Nazarov reaction to afford the cyclized intermediate **9**, which is stabilized by the trimethylsilyl (TMS) group via the β-silicon stabilization effect [36–38] as supported by computational studies [35]. The desilylation of intermediate **9** with the chloride of another molecule of **8** would result in the formation of final aza-Nazarov product **7** along with TMSCl (Scheme 1d). Five-membered nitrogen-heterocycles [39–45] and specifically α-methylene-γ-lactams as a subset of this compound class, are popular targets in heterocyclic chemistry and drug discovery [46–56]. Against this background, we herein disclose a full account of our studies on the catalytic aza-Nazarov reaction starting from cyclic and acyclic imines and TMS-substituted α,β-unsaturated acyl chlorides to yield α-methylene-γ-lactam heterocycles with high diastereoselectivities. We also report the results of our detailed mechanistic studies along with the necessary control experiments in order to shed light on the mechanism and specific features of this transformation.

## Results and Discussion

In the initial phase of our work, we used AgOTf as an anion-exchange agent in order to promote the desired aza-Nazarov cyclization [35]. The reaction between 3,4-dihydoisoquinoline (**5a**) and acyl chloride **6a** proceeds well at 23 °C in CH_2_Cl_2_ with the use of a stoichiometric amount (1.3 equiv) of AgOTf providing the aza-Nazarov product **7a** in 57% yield and as single diastereomer, whereas no cyclization was observed in the absence of AgOTf (Table 1, entries 1 and 2). When the same reaction was tested at 80 °C in CH_3_CN, a substoichiometric amount of AgOTf (20 mol %) was found to be sufficient to efficiently promote the reaction to give product **7a** in 79% yield (Table 1, entry 3). When AgOTf was not used under these conditions, the reaction was observed to proceed only with 13% yield (Table 1, entry 4). The scope of the reaction with 3,4-dihydroisoquinoline substrates as cyclic imines was investigated under these conditions [35]. In the current work, we first opted to examine the activities of other Lewis acids in the aza-Nazarov cyclization of imine **5a** with acyl chloride **6b**. The reason of using acyl chloride **6b** with an isobutyl side chain is its low volatility in contrast to the highly volatile compound **6a**. The aza-Nazarov product **7b** was isolated in 61% yield with 20 mol % of AgOTf at 80 °C (Table 1, entry 5). The use of TMSOTf as a Si-based Lewis acid catalyst with 20 mol % loading afforded the cyclization product **7b** in 47% yield (Table 1, entry 6), whereas the activity of Zn(OTf)_2_ was observed to be lower providing the desired product in only 36% yield (entry 7). Surprisingly, NaBF_4_ appeared to be effective for the aza-Nazarov reaction, and the α-methylene-γ-lactam product **7b** was isolated in 54% yield (Table 1, entry 8). The exchange of chloride with the non-coordinating BF_4_^−^ anion, driven by the precipitation of NaCl, is proposed to be responsible for this positive result.

**Table 1:** Screening of Lewis acids and hydrogen-bond donors (HBD) for the aza-Nazarov cyclization.^a^

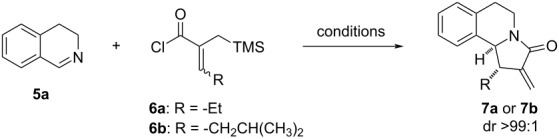					

Entry	Lewis acid or HBD (equiv)	Solvent	*T* (°C)	Product	Yield (%)^b^

1	AgOTf (1.3)	CH_2_Cl_2_	23	**7a**	57
2	–	CH_2_Cl_2_	23	**7a**	<1
3	AgOTf (0.2)	CH_3_CN	80	**7a**	79
4	–	CH_3_CN	80	**7a**	13
5^c^	AgOTf (0.2)	CH_3_CN	80	**7b**	61
6	TMSOTf (0.2)	CH_3_CN	80	**7b**	47
7	Zn(OTf)_2_ (0.2)	CH_3_CN	80	**7b**	36
8	NaBF_4_ (0.2)	CH_3_CN	80	**7b**	54
9	**10** (0.2)	CH_2_Cl_2_	23	**7b**	<1
10	**10** (1.2)	CH_2_Cl_2_	23	**7b**	15
11	**10** (0.2)	CH_3_CN	80	**7b**	40
12	**11** (1.2)	CH_2_Cl_2_	23	**7b**	22
13	**11** (0.2)	CH_3_CN	80	**7b**	48
14	**12** (1.2)	CH_2_Cl_2_	23	**7b**	17
15	**12** (0.2)	CH_3_CN	80	**7b**	59
16	**13** (1.2)	CH_2_Cl_2_	23	**7b**	25

^a^1.3 equiv of acyl chloride **6** and 1.0 equiv of imine **5a** were reacted for 22 h. ^b^Yields refer to isolated product yields after purification by column chromatography. ^c^Reaction scale: 1.0 mmol.
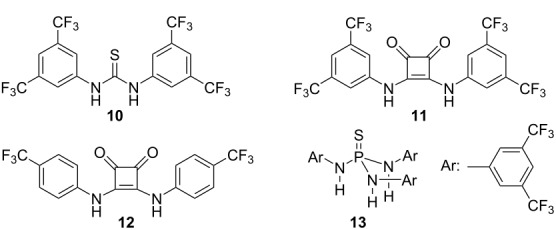

Next, we turned our attention to the use of hydrogen-bond donors as anion-binding catalysts [57–58]. To this end, we first tested the achiral thiourea derivative **10**, which exhibited very low activity at 23 °C when used in catalytic and stoichiometric amounts (Table 1, entries 9 and 10) [59–60]. The yield of the aza-Nazarov product **7b** increased to 40% when 20 mol % of thiourea **10** were used at 80 °C in CH_3_CN (Table 1, entry 11). With this promising result in hand, we next examined the use of squaramides, which were shown to be highly effective hydrogen-bonding catalysts in a broad range of transformations [61–64]. When achiral squaramide derivatives **11** [65] and **12** [66] were tested in stoichiometric amounts at 23 °C in CH_2_Cl_2_, the aza-Nazarov product **7b** was formed in low yields (22 and 17%, Table 1, entries 12 and 14, respectively). However, the yields of the isolated products increased to 48 and 59% with the use of catalytic amounts of **11** and **12**, respectively, at 80 °C (Table 1, entries 13 and 15). Finally, we investigated the activity of the achiral thiophosphoric triamide **13** as a triple hydrogen-bond donor [67]. With the use of 1.2 equiv of **13** at 23 °C, product **7b** was isolated in 25% yield (Table 1, entry 16). Overall, while AgOTf still appeared to be the best reagent to be used in catalytic amount to promote the aza-Nazarov reactions investigated in this study, the results discussed above showcase the potential of strong hydrogen-bond donors as effective anion binding catalysts for this transformation. Finally, it should be noted that all aza-Nazarov products that are presented in Table 1, were obtained as single diastereomers.

**Scheme 2:**
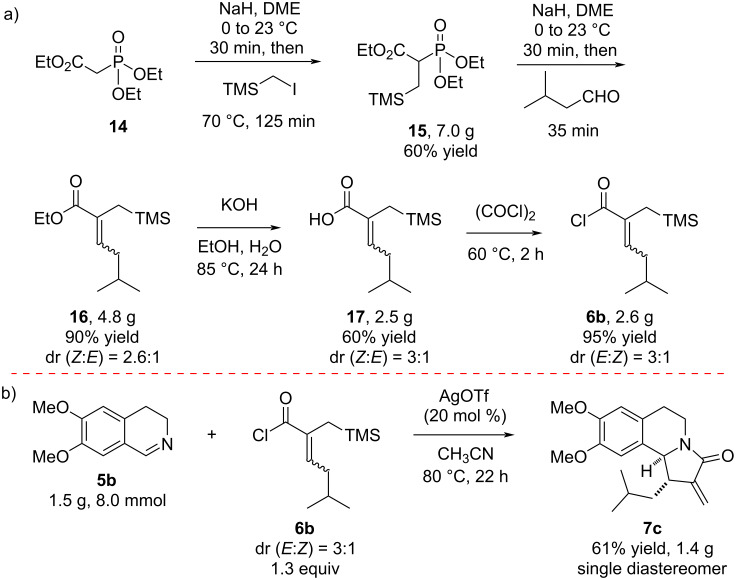
Aza-Nazarov cyclization on gram scale.

In order to assess the scalability of the aza-Nazarov cyclization, we investigated the synthesis of the previously unreported lactam **7c** via the reaction between imine **5b** and acyl chloride **6b** on a gram scale (Scheme 2). Acyl chloride **6b** was prepared in four steps starting from the commercially available triethyl phosphonoacetate (**14**, Scheme 2a). The deprotonation of **14** with NaH followed by alkylation using (iodomethyl)trimethylsilane afforded phosphonate **15** in 60% yield (7.0 g). Its subsequent Horner–Wadsworth–Emmons reaction with isovaleraldehyde resulted in the formation of ester **16** in 90% yield (4.8 g, dr (diastereomeric ratio) = 2.6:1). Hydrolysis of the α,β-unsaturated ester **16** under basic conditions followed by acidification gave carboxylic acid **17** in 60% yield. Finally, the α,β-unsaturated acyl chloride **6b** was obtained via the treatment of acid **17** with oxalyl chloride at 60 °C in 95% yield (2.6 g) and with a dr (*E*:*Z*) of 3:1 (Scheme 2a). When imine **5b** was reacted with **6b** on an 8.0 mmol scale with the use of AgOTf (20 mol %) in acetonitrile at 80 °C, the aza-Nazarov product **7c** was isolated in 61% yield (1.42 g) as a single diastereomer. This result clearly demonstrates the preparative value of the catalytic aza-Nazarov cyclization developed in this work.

**Scheme 3:**
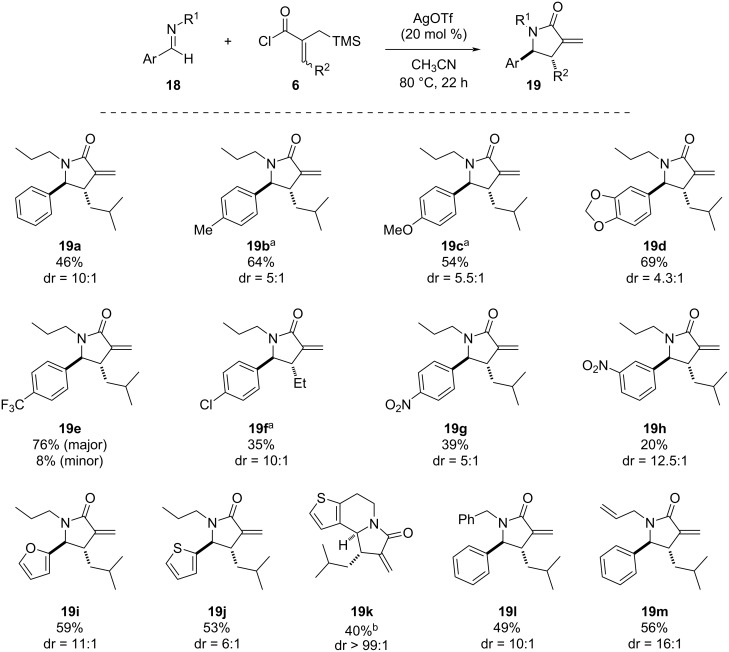
Scope of the aza-Nazarov cyclization with acyclic imines. ^a^The syntheses of aza-Nazarov products **19b**, **19c**, and **19f** were described previously [35]. ^b^The reaction was carried out at 23 °C in CH_2_Cl_2_ using 1.3 equiv of AgOTf.

Following the success of the newly developed aza-Nazarov reaction with 3,4-dihydroisoquinoline derivatives as cyclic imines [35], we next opted to investigate its effectiveness with acyclic imine substrates. The aza-Nazarov reactions of such imines were found to proceed successfully, albeit with slightly lower diastereoselectivities (ca. 4:1 to 16:1 dr) compared to their cyclic counterparts (Scheme 3). The reaction of the aldimine formed via the condensation of benzaldehyde and *n*-propylamine with acyl chloride **6b** gave the aza-Nazarov product **19a** in 46% yield and with a dr of 10:1 when 20 mol % AgOTf were used as anion exchange reagent in CH_3_CN at 80 °C. Aldimines with electron-donating substituents were found to be suitable substrates for this transformation affording the α-methylene-γ-lactam products **19b**–**d** in good yields (54–69%). We were pleased to see that the CF_3_-substituted aza-Nazarov product **19e** was isolated as single diastereomer in 76% yield along with only 8% of the minor diastereomer. Surprisingly, aza-Nazarov products **19f** with a 4-Cl substituent on the phenyl ring as well as **19g** and **19h** bearing a strongly electron-withdrawing NO_2_ substituent at the 4- and 3-positions were obtained in lower yields (35, 39 and 20%, respectively) under the optimized conditions. We next turned our attention to the use of heteroaromatic side chains on the imine component. Gratifyingly, the reaction was found to tolerate the presence of furan and thiophene rings where 2-furyl- and thienyl-substituted aza-Nazarov products **19i** and **19j** were isolated in 59 and 53% yields, and with a dr of 11:1 and 6:1, respectively. Inspired by the success of the thiophene-containing imine reactant, we also wanted to check the reactivity of a thiophene-fused dihydropyridine as a cyclic imine. The tricyclic α-methylene-γ-lactam product **19k** was obtained in 40% yield and as single diastereomer. It should be noted that due to the low solubility of the imine substrate in CH_3_CN, this reaction was carried out in CH_2_Cl_2_ at 23 °C and with the use of 1.3 equivalents of AgOTf. Finally, we sought to check the reactivity of other substituents on the amine component of acyclic imine substrates. With the use of benzyl-substituted aldimine, the aza-Nazarov product **19l** was obtained in 49% yield and with 10:1 dr. Similarly, the aza-Nazarov cyclization of the allyl-substituted imine substrate afforded α-methylene-γ-lactam product **19m** in 56% yield.

**Figure 1:**
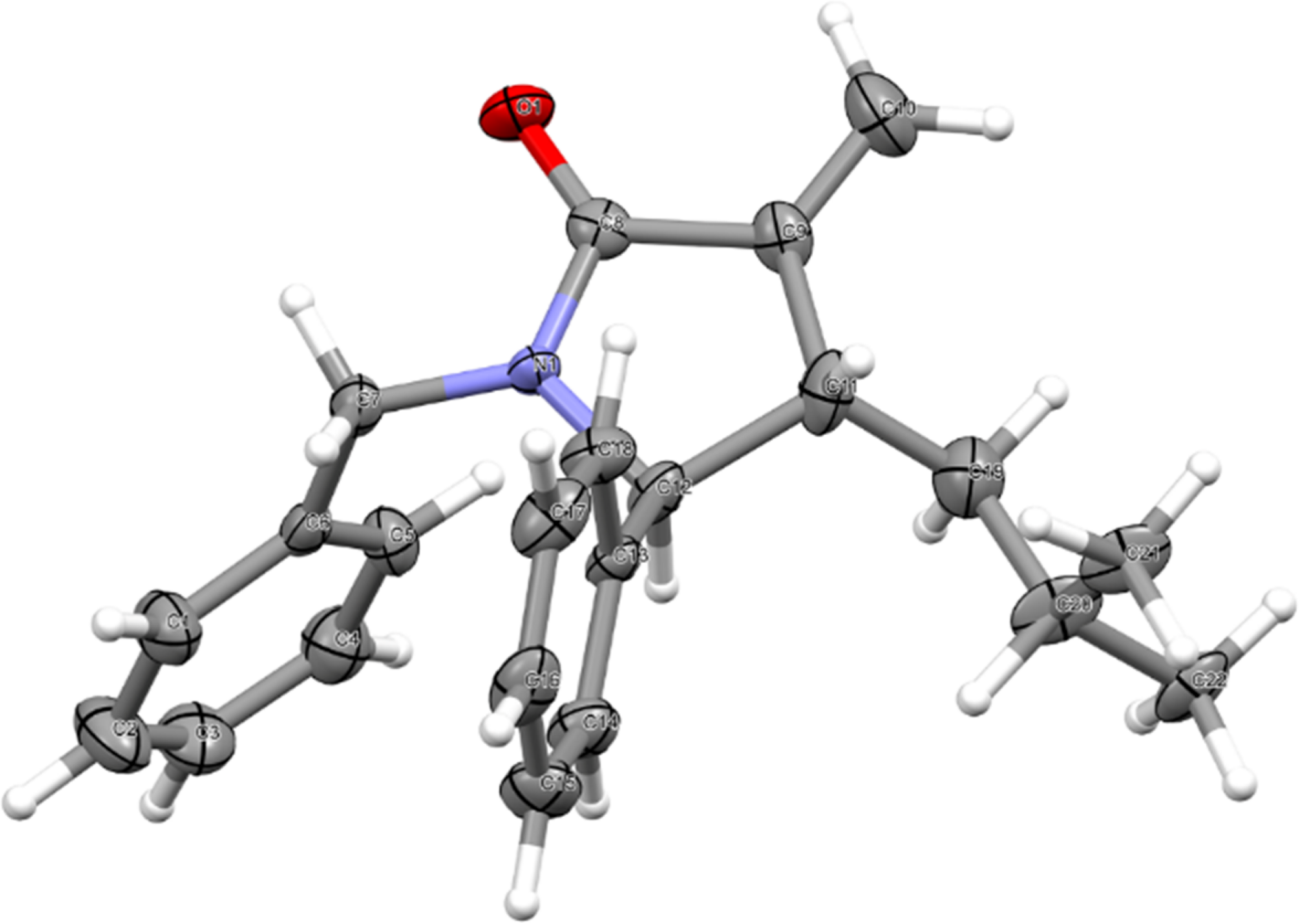
X-ray crystal structure of compound **19l**.

In our previous study, the relative stereochemistry of a tricyclic aza-Nazarov product obtained from a 3,4-dihydroisoquinoline derivative was secured by single-crystal X-ray analysis [35]. In the present work, the α-methylene-γ-lactam products **19** were in most cases isolated as oils after column chromatography. Fortunately, lactam product **19l** turned out to be a solid, and high-quality crystals could be obtained via slow vapor diffusion of pentane into its CH_2_Cl_2_ solution. Single-crystal X-ray analysis revealed that the relative stereochemistry of lactam **19l** with the isobutyl and phenyl groups in a *trans* arrangement (Figure 1, CCDC 2116978). This finding confirms that both cyclic and acyclic imines undergo the aza-Nazarov cyclization developed in this work via the same stereochemical arrangement.

**Scheme 4:**
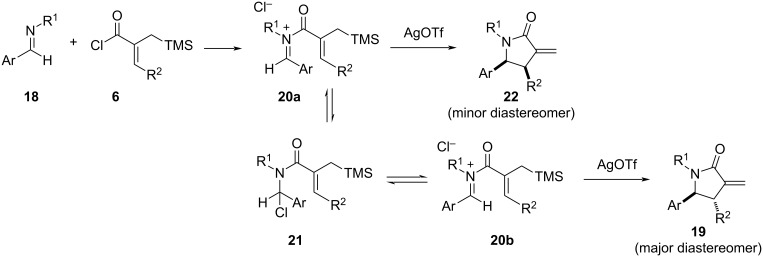
Proposed mechanism for the formation of diastereomers **19** and **22**.

Another intriguing aspect of the aza-Nazarov reactions performed in the current work with acyclic imines is the formation of the *cis* isomer as the minor diastereomer in contrast to the reactions of 3,4-dihydroisoquinoline derivatives where the aza-Nazarov products are obtained as single diastereomers. This reaction outcome was attributed to a potential *cis*–*trans* isomerization of the C–N double bond upon iminium formation (Scheme 4). In this respect, the mixing of imine **18** with the α,β-unsaturated acyl chloride **6** is expected to form the iminium ion **20a** with *Z*-configuration. A chloride-mediated iminium *E*–*Z* isomerization may take place through the intermediacy of α-chloroamide **21**. The aza-Nazarov reaction of the more stable *E*-iminium ion **20b** is expected to proceed faster due to steric considerations giving the major diastereomer **19** whereas the less stable *Z*-iminium ion **20a** would provide the minor diastereomer **22**. In the case of aza-Nazarov reactions of 3,4-dihydroisoquinoline derivatives, an *E*–*Z* isomerization of the iminium intermediates is not possible due to their cyclic nature, which leads to the formation of the aza-Nazarov products as single diastereomers.

**Scheme 5:**
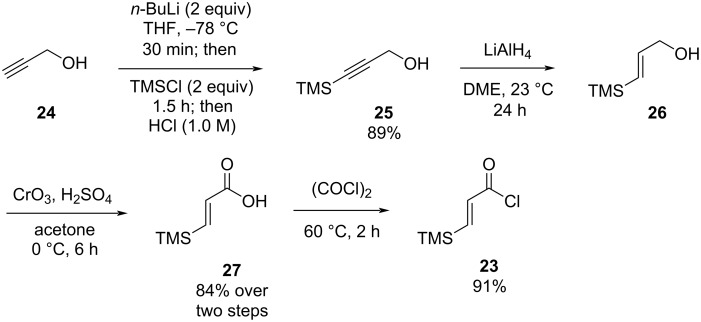
Preparation of acyl chloride **23**.

The aza-Nazarov reactions that we examined so far involved the use of α,β-unsaturated acyl chlorides having a -CH_2_TMS group at the α-position. Next, we decided to test the reactivity of an α,β-unsaturated acyl chloride possessing a TMS group at the β-position, which would still be suitable to take advantage of the β-silicon effect. For this purpose, we prepared the known acyl chloride **23** in four steps starting from propargyl alcohol (Scheme 5) [68]. Trimethylsilylation of propargyl alcohol (**24**, 89% yield) followed by reduction of the alkyne using LiAlH_4_ afforded the allylic alcohol **26** as a single (*E*) diastereomer. Oxidation of **26** with Jones reagent afforded the carboxylic acid **27** (84% over two steps) and its subsequent treatment with oxalyl chloride gave the desired β-TMS-substituted acyl chloride **23** in 91% yield.

**Scheme 6:**
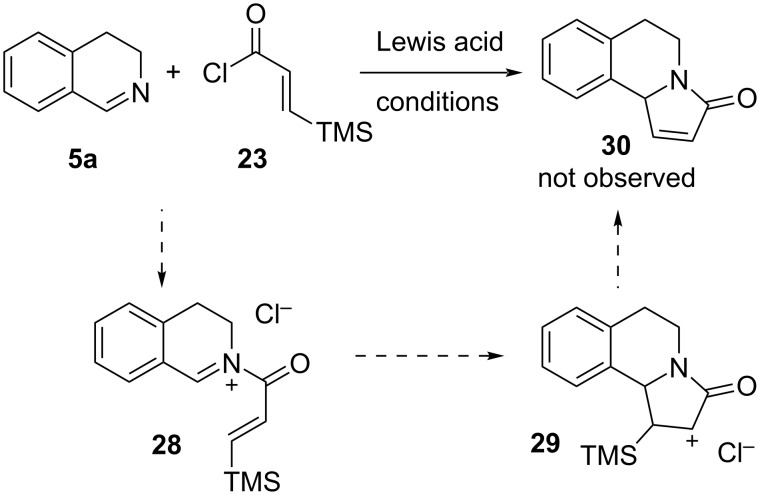
Aza-Nazarov reaction tested using β-TMS-substituted acyl chloride **23**.

Based on our previous observations, the reaction of imine **5a** with acyl chloride **23** was expected to give *N*-acyliminium ion **28**, the aza-Nazarov cyclization of which would provide the final product **30** through the intermediacy of **29** (Scheme 6). According to our hypothesis, the carbocation of intermediate **29** would be stabilized by the β-silicon effect similar to intermediate **9** (vide supra). Unfortunately, screening a variety of conditions including the use of stoichiometric and substoichiometric amounts of Lewis acids such as AgOTf and BF_3_·OEt_2_ with and without heating (23 and 80 °C) did not afford any targeted aza-Nazarov product **30** (for details see Table S1 in Supporting Information File 1). The reactivity differences between acyl chlorides **23** and **6** in the examined aza-Nazarov cyclizations can be understood when the electron densities on the two olefin moieties are considered. Indeed, while both of the proposed intermediates **29** and **9** (Scheme 1) can benefit from the β-silicon stabilization effect, the olefinic group of intermediate **8** is expected to be more nucleophilic than that of intermediate **28** given the fact that allylsilanes possess a significantly higher nucleophilicity than vinylsilanes [69]. Therefore, the electron richness of the nucleophilic alkene appears to be an important parameter in addition to the β-silicon effect in this aza-Nazarov cyclization.

**Scheme 7:**
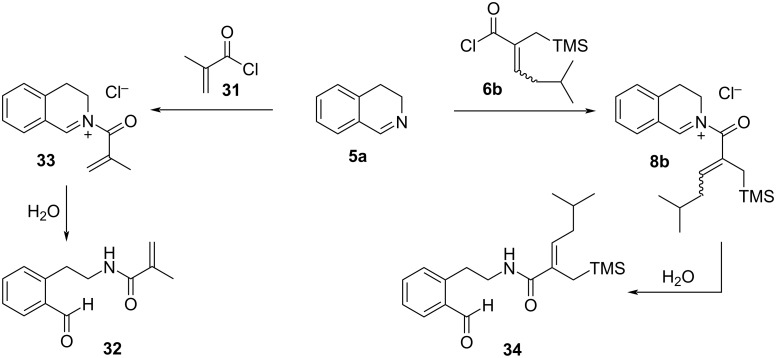
Hydrolysis of *N*-acyliminium intermediates.

During the course of our studies, we observed occasionally the formation of aldehyde-containing side products, the amount of which increased when the aza-Nazarov cyclizations did not proceed efficiently. We proposed that, if the aza-Nazarov cyclization of an in situ-formed iminium intermediate is not efficient under certain reaction conditions, then its hydrolysis with adventitious water, which might be present in the reaction medium, would lead to the formation of an aldehyde side product. Unfortunately, our attempts to isolate such a side product in pure form from a crude reaction mixture failed. However, when a mixture of imine **5a** and methacryloyl chloride (**31**) was stirred in a biphasic mixture of CH_2_Cl_2_ and aqueous NaHCO_3_ solution, we were able to isolate and fully characterize aldehyde **32** which would form via the hydrolysis of iminium ion **33** (Scheme 7). Pleasingly, the same approach worked successfully with the acyl chloride **6b** where aldehyde product **34** was isolated in diastereomerically pure form (Scheme 7). The ^1^H NMR spectral data of **34** matched those observed in the spectra of the crude reaction mixtures. These findings underscore the importance of maintaining strictly anhydrous reaction conditions during the aza-Nazarov cyclization for achieving high reaction yields.

**Scheme 8:**
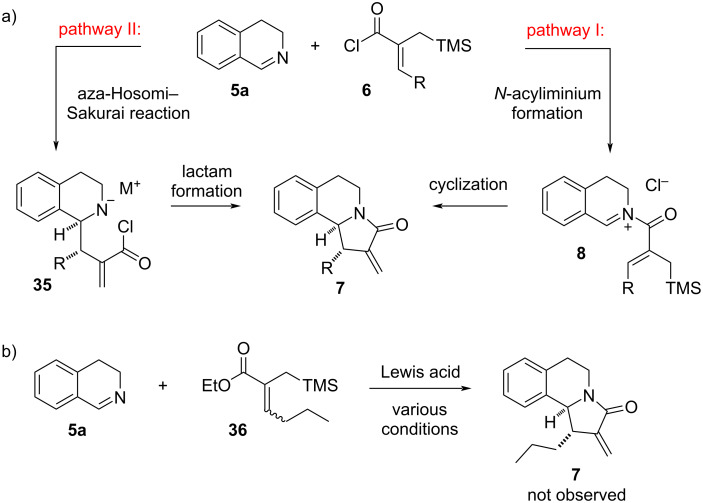
(a) Two possible pathways for the formation of **7** and (b) investigation of the reaction between imine **5a** and ester **36**.

Regarding the reaction between imine **5a** and acyl chloride **6** to yield final product **7** two pathways can be envisaged as shown in Scheme 8a. According to the originally proposed pathway I, the initial formation of *N*-acyliminium ion **8** followed by an aza-Nazarov cyclization would lead to product **7**. Alternatively, an aza-Hosomi–Sakurai-type reaction may be considered as the initial step between imine **5a** and the allylsilane moiety of **6** to give intermediate **35**, even though the nucleophilicity of the allylsilane is expected to be low due to the presence of the electron-withdrawing acyl chloride functionality (pathway II, Scheme 8a). The subsequent lactam formation in intermediate **35** would give the final product **7**. While general reactivity considerations and the aforementioned formation of the hydrolysis side product **34** support pathway I, we sought to design an experiment to rule out pathway II. To this end, we investigated the potential reaction between imine **5a** and α,β-unsaturated ester **36** in the presence of a variety of Lewis acids (Scheme 8b). The reason of using ester **36** with an *n*-propyl group at the β-position is that it does not have the volatility issues of the ethyl-substituted ester, and that it is sterically less hindered than the isobutyl-substituted ester. When a broad range of Lewis and Brønsted acids such as AgOTf, Zn(OTf)_2_, ZnBr_2_, CuCl_2_, BF_3_·OEt_2_, and Tf_2_NH were tested at different temperatures (Table S2 in Supporting Information File 1) in the reaction between imine **5a** and ester **36**, the reactants were observed to remain intact, and cyclization product **7** or any other side products, which may be indicative of an aza-Hosomi–Sakurai reaction, were not observed (Scheme 8b). These results strongly support pathway I for the formation of **7** starting from **5a** and acyl chloride **6**.

**Scheme 9:**
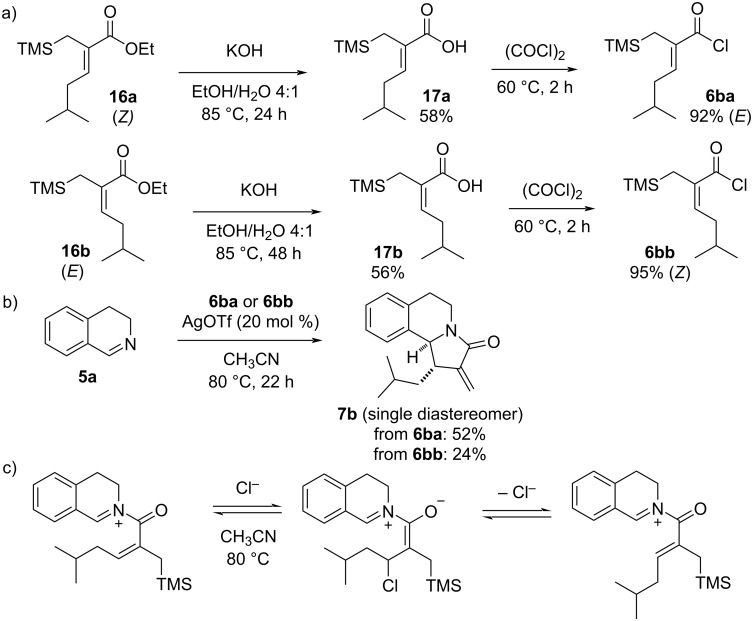
(a) Preparation of acyl chlorides **6ba** and **6bb** in diastereomerically pure forms, (b) aza-Nazarov cyclization of (*E*)-**6ba** and (*Z*)-**6bb** under the optimized reaction conditions and (c) tentative mechanism for the olefin *E*–*Z* isomerization.

In the present work, the α,β-unsaturated acyl chlorides were generally used as mixtures of diastereomers (typically dr = 3:1 or 4:1) due to the difficulties in the isolation of pure (*E*)- and (*Z*)-isomers. The aza-Nazarov cyclization of such diastereomeric mixtures always resulted in the formation of products as single diastereomers with cyclic imines (e.g., imine **5a**) [35]. We initially attributed this observed diastereoselectivity to the reaction taking place only with the major (*E*)-isomer of the acyl chloride based on steric considerations. However, in order to show lack of reactivity of the minor (*Z*)-isomer, we sought to isolate the two diastereomers in pure form. After extensive efforts, a mixture of benzotrifluoride (PhCF_3_) and hexanes was identified as a suitable mobile phase for the column chromatographic separation of the diastereomeric esters **16a** and **16b**. With the successful isolation of these esters in pure form, both were converted to the corresponding acyl chlorides **6ba** and **6bb** via an initial basic hydrolysis followed by treatment with oxalyl chloride (Scheme 9a). As expected, the aza-Nazarov product **7b** was obtained as single diastereomer in 52% yield when the major acyl chloride **6ba** with (*E*)-configuration was subjected to the reaction conditions (Scheme 9b). Surprisingly, the minor (*Z*)-isomer **6bb** also gave the same diastereomerically pure aza-Nazarov product **7b**, albeit in a lower yield (24%). The fact that the other diastereomer of the aza-Nazarov product **7b**, which would be formed via the reaction of acyl chloride **6bb** with (*Z*)-olefin configuration, was not observed in this transformation points to a potential *E*–*Z* isomerization during the reaction. This type of olefin *E*–*Z* isomerization is likely to take place after the *N*-acyliminium formation, possibly via a Michael/retro-Michael addition sequence between the chloride (Cl^−^) anion and the electron-deficient olefin of the *N*-acyliminium intermediate at 80 °C (Scheme 9c). In conclusion, these results show that while both isomers of the acyl chloride (**6ba** and **6bb**) are capable of undergoing the aza-Nazarov cyclization with imine **5a** affording the same cyclization product, the major (*E*)-isomer is understandably more reactive due to the favorable sterics of the C–C-bond-forming cyclization step.

## Conclusion

In summary, we developed an effective aza-Nazarov reaction of 3,4-dihydroisoquinolines and acyclic imines with α,β-unsaturated acyl chlorides to yield α-methylene-γ-lactam heterocyclic products. Whereas a variety of metal-based Lewis acids and organic hydrogen-bond donors were shown to be capable of catalyzing the reaction via anion binding, AgOTf stands out as the optimal Lewis acid for this transformation. The aza-Nazarov cyclization of 3,4-dihydroisoquinolines with TMS-substituted α,β-unsaturated acyl chlorides proceeds efficiently in the presence of AgOTf (20 mol %) in CH_3_CN at 80 °C to afford tricyclic lactam products in up to 79% yield and as single diastereomers. Moreover, acyclic imines were shown to be competent substrates for this reaction where substituted α-methylene-γ-lactam heteocycles were obtained in up to 76% yield and with dr values of 4.3:1 to 16:1. Detailed mechanistic and control experiments provided valuable insights on the reaction mechanism including the importance of the β-silicon effect and the alkene geometry of the α,β-unsaturated acyl chloride reactants on reactivity, different potential modes of cyclization, and the effect of adventitious water on the aza-Nazarov cyclization.

## Supporting Information

File 1 Experimental procedures, characterization data, and copies of ^1^H and ^13^C{^1^H} NMR spectra.

File 2 CIF file of compound **19l**.
