# Supplementary material for: Catalytic aza-Nazarov cyclization reactions to access α-methylene-γ-lactam heterocycles
Source: Beilstein J Org Chem. 2023 Jan 17;19:66–77. doi: 10.3762/bjoc.19.6 (PMC9874235; doi:10.3762/bjoc.19.6)
Supplement: File 1 — Experimental procedures, characterization data, and copies of 1H and 13C{1H} NMR spectra. [file Beilstein_J_Org_Chem-19-66-s001.pdf]

BEILSTEIN JOURNAL OF ORGANIC CHEMISTRY

Supporting Information

for

Catalytic aza-Nazarov cyclization reactions to access α-methylene-γ-lactam heterocycles

Bilge Banu Yagci, Selin Ezgi Donmez, Onur Şahin and Yunus Emre Türkmen

Beilstein J. Org. Chem. **2023,** *19,* 66–77. doi:10.3762/bjoc.19.6

Experimental procedures, characterization data, and copies of ^{1}H and $^{13}C\{^{1}H\}$ NMR spectra

License and Terms: This is a supporting information file under the terms of the Creative Commons Attribution License (https://creativecommons.org/licenses/by/4.0). Please note that the reuse, redistribution and reproduction in particular requires that the author(s) and source are credited and that individual graphics may be subject to special legal provisions.
The license is subject to the *Beilstein Journal of Organic Chemistry* terms and conditions: (https://www.beilstein-journals.org/bjoc/terms)

Table of contents

General information and materials S2
General procedure A for the catalyst screening S3
Gram-scale synthesis of acyl chloride **6b** S3
Gram-scale aza-Nazarov reaction S5
Syntheses of aza-Nazarov products **19** S6
List of unsuccessful substrates in the aza-Nazarov reaction S16
Preparation of acyl chloride **23** S16
Table S1: Investigation of the aza-Nazarov reaction with acyl chloride **23** S18
Isolation of aldehydes **32** and **34** S19
Table S2: Investigation of the aza-Hosomi–Sakurai reaction between imine **5a** and ester **36** S20
Synthesis of acyl chlorides **6ba** and **6bb** S21
X-ray diffraction analysis of aza-Nazarov product **19l** S24
References S26
^{1}H NMR and $^{13}C\{^{1}H\}$ NMR spectra S27

General Information: All reactions were performed using oven-dried glassware under an inert atmosphere of nitrogen. Reactions were monitored by thin-layer chromatography (TLC) using aluminum-backed plates precoated with silica gel (Merck or Silicycle, Silica Gel 60 F_{254}). UV light and/or $KMnO_4$ and PMA (phosphomolybdic acid) staining solutions were used for TLC visualization. Flash column chromatography was performed on Silicycle 40–63 μm (230–400 mesh) flash silica gel. NMR spectra were measured on a Bruker spectrometer at 400 MHz for ^1H NMR spectra and 100 MHz for $^{13}C\{^1H\}$ NMR spectra. The spectra were calibrated with internal standard (TMS, 0 ppm) or residual solvent signals (chloroform at 7.26 ppm for ^1H NMR spectra and at 77.16 ppm for $^{13}C\{^1H\}$ NMR spectra). ^1H NMR data are reported as follows: chemical shift (parts per million, ppm), integration, multiplicity (s = singlet, d = doublet, t = triplet, dd = doublet of doublets, ddd = doublet of doublet of doublets, m = multiplet, br = broad, app = apparent), coupling constant (Hz). Infrared (FTIR) spectra were recorded on a Bruker Alpha-Platinum-ATR spectrometer with only selected peaks reported. Mass spectral analyses were performed at UNAM-National Nanotechnology Research Center and Institute of Materials Science and Nanotechnology, Bilkent University. XRD analysis was performed at the Scientific and Technological Research Application and Research Center, Sinop University, Turkey.

Materials: Anhydrous CH_3CN was obtained by distillation over P_2O_5 under an inert atmosphere of nitrogen. Anhydrous CH_2Cl_2 and DME were purchased from Acros Organics (AcroSeal®) and used as received. Silver trifluoromethanesulfonate (AgOTf) (99+%) was purchased from Acros Organics, and stored in a desiccator. All other commercially available reagents were used as received unless stated otherwise.

3,4-Dihydroisoquinoline derivatives **5a** and **5b** were prepared following literature procedures.[1] The syntheses of the aza-Nazarov products **19b**, **19c**, and **19f** were described in our initial communication.[1]

General procedure A for the catalyst screening:

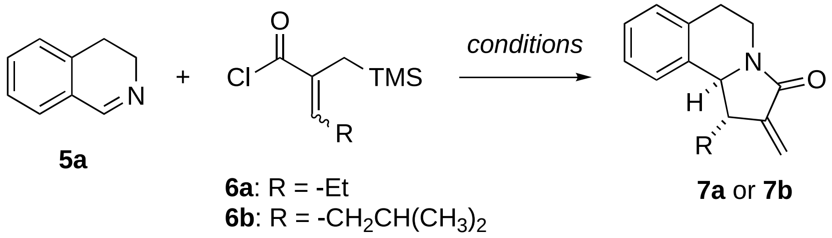

A 25-mL, oven-dried Schlenk flask was cooled to ambient temperature under an inert atmosphere of nitrogen and charged with imine **5a** (1.0 equiv) and 0.5–1.0 mL of anhydrous CH_2Cl_2 or CH_3CN. The solution of acyl chloride reactant (1.3 equiv) in 0.5–1.0 mL of anhydrous CH_2Cl_2 or CH_3CN was added to the reaction mixture, and the resulting clear solution was stirred for 5 min at rt. After the addition of Lewis acid or hydrogen-bond donor (0.2 or 1.2 equiv), the reaction mixture was stirred at 23 °C or 80 °C for 22 h. It was then quenched at ambient temperature with saturated $NaHCO_{3(aq)}$ solution. The aqueous phase was extracted three times with EtOAc. The combined organic layers were dried over Na_2SO_4, filtered, and concentrated under reduced pressure. Purification of the product was carried out by flash column chromatography using SiO_2 as the stationary phase.

Gram-scale synthesis of acyl chloride 6b:

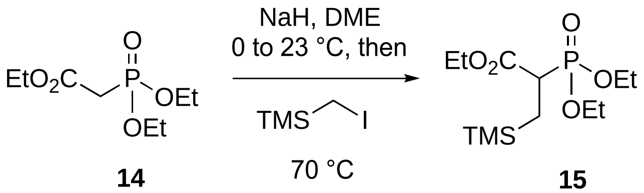

Compound **15** was prepared following a procedure reported in the literature.[2] To a solution of triethyl phosphonoacetate (**14**, 4.00 g, 3.57 mL, 18.7 mmol) in DME (8.0 mL) was added NaH (749 mg, 18.7 mmol, 60% dispersion in mineral oil) as a solid at 0 °C under an inert atmosphere of nitrogen. After the reaction mixture was stirred at 23 °C for 30 min, $TMSCH_2I$ (4.59 g, 3.18 mL, 21.4 mmol) was added. The resulting mixture was stirred at 70 °C for 125 min, and the progress of the reaction was monitored by TLC. The reaction mixture was then cooled to ambient temperature and quenched with a saturated aqueous solution of NH_4Cl. The aqueous phase was extracted three times with $CHCl_3$. The combined organic phase was dried over Na_2SO_4, filtered, and concentrated under reduced pressure.

This reaction was repeated twice, and the crude mixtures were combined.

Purification by flash column chromatography (EtOAc/hexanes 1:1) gave pure product **15** as a colorless oil (6.997 g, 60% yield).

The spectral data match those reported in the literature.[1,2]

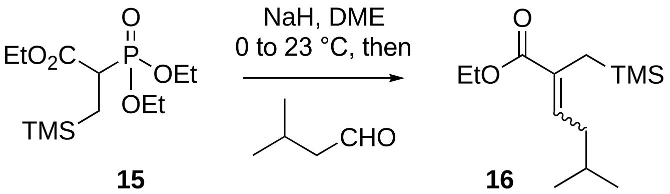

Experiment 1:

To a solution of phosphonate **15** (3.47 g, 11.2 mmol) in DME (15.0 mL) was added NaH (667 mg, 16.8 mmol, 60% dispersion in mineral oil) at 0 °C under nitrogen. The mixture was stirred at 23 °C for 30 min, and then isovaleraldehyde (1.45 g, 1.80 mL, 16.8 mmol) was added. The resulting mixture was stirred at 23 °C for an additional 35 min, and then quenched with a saturated aqueous solution of NH_4Cl. The aqueous phase was extracted three times with CH_2Cl_2. The combined organic phase was dried over Na_2SO_4, filtered, and concentrated under reduced pressure.

Experiment 2:

To a solution of phosphonate **15** (3.42 g, 11.0 mmol) in DME (15.0 mL) was added NaH (660 mg, 16.5 mmol, 60% dispersion in mineral oil) at 0 °C under nitrogen. The mixture was stirred at 23 °C for 30 min, and then isovaleraldehyde (1.45 g, 1.80 mL, 16.8 mmol) was added. The resulting mixture was stirred at 23 °C for an additional 35 min, and then quenched with a saturated aqueous solution of NH_4Cl. The aqueous phase was extracted three times with CH_2Cl_2. The combined organic phase was dried over Na_2SO_4, filtered, and concentrated under reduced pressure.

Crude mixtures from experiment 1 and 2 were combined.

Purification by flash column chromatography (EtOAc/hexanes 1:100) gave product **16** as a yellow oil (4.832 g, 90% yield, *Z*:*E* = 2.6:1).

The spectral data match those reported in the literature.[1]

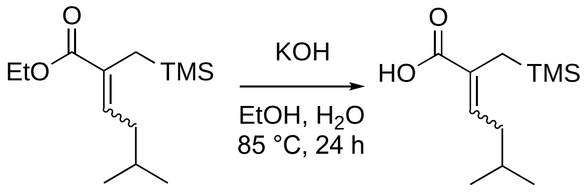

16 **17**

To a solution of ester **16** (4.666 g, 19.25 mmol) in 20% H_2O in EtOH (15.0 mL) was added KOH (3.294 g, 58.7 mmol) at 23 °C, and the mixture was stirred at 85 °C for 24 h. It was then cooled to ambient temperature and quenched with 1.0 M $HCl_{(aq)}$ solution, followed by a saturated aqueous solution of NH_4Cl. The aqueous phase was extracted three times with CH_2Cl_2. The combined organic phase was dried over Na_2SO_4, filtered, and concentrated under reduced pressure. Purification by flash column chromatography (EtOAc/hexanes 1:5) gave product **17** as a yellow oil (2.489 g, 60% yield, *Z*:*E* = 3:1).

The spectral data match those reported in the literature.[1]

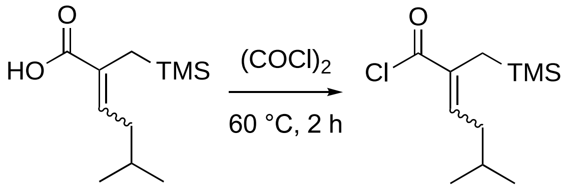

17 **6b**

A solution of **17** (2.489 g, 11.6 mmol) in 10.0 mL of $(COCl)_2$ (oxalyl chloride) was heated at 60 °C for 2 h. It was then cooled to ambient temperature, and removal of excess $(COCl)_2$ using a rotary evaporator inside a well-ventilated fume hood gave **6b** as an orange oil (2.571 g, 95% yield, *E*:*Z* = 3:1).

The spectral data match those reported in the literature.[1]

Note: This product is sensitive to water. Therefore, it was used immediately after preparation.

Gram-scale aza-Nazarov reaction:

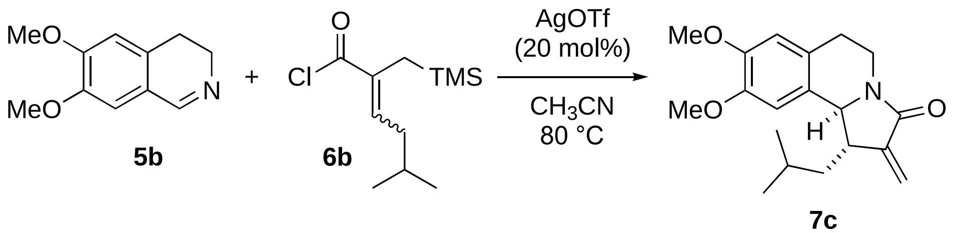

A 100-mL, round-bottomed flask was cooled to ambient temperature under nitrogen and charged with imine **5b** (1.538 g, 8.04 mmol) and 5.0 mL of anhydrous CH_3CN. A solution of acyl chloride **6b** (2.433 g, 10.45 mmol, dr = 3:1) in 4.8 mL of anhydrous CH_3CN was added to the reaction mixture, and the resulting clear solution was stirred for 5 min at 23 °C. After the addition of AgOTf (414 mg, 1.61 mmol), the solution turned cloudy. The flask was inserted into a preheated oil bath at 80 °C, and the reaction mixture was stirred at this temperature for 22 h. It was then cooled to ambient temperature and quenched with a saturated aqueous solution of $NaHCO_3$ (10 mL). The aqueous phase was extracted with CH_2Cl_2 (3 × 15 mL). The combined organic phase was dried over Na_2SO_4, filtered, and concentrated under reduced pressure. Purification by flash column chromatography (EtOAc/hexanes 1:1 to only EtOAc) gave pure aza-Nazarov product **7c** as an orange oil (1.421 g, 61% yield) as single diastereomer.

R$_f$ = 0.50 (only EtOAc)

^{1}H NMR (400 MHz; $CDCl_3$) δ: 6.64 (1H, s), 6.57 (1H, s), 6.01 (1H, d, *J* = 2.5 Hz), 5.29 (1H, d, *J* = 1.9 Hz), 4.39-4.34 (2H, m), 3.84 (3H, s), 3.83 (3H, s), 3.13 (1H, ddd, *J* = 12.7, 11.3, 4.7 Hz), 3.03-2.92 (2H, m), 2.64-2.59 (1H, m), 2.02-1.92 (1H, m), 1.75-1.59 (2H, m), 1.09 (3H, d, *J* = 6.5 Hz), 1.05 (3H, d, *J* = 6.6 Hz).

^{13}C{^{1}H} NMR (100 MHz; $CDCl_3$) δ: 166.8, 148.2, 148.0, 145.0, 129.5, 126.5, 116.0, 112.0, 107.8, 61.0, 56.0, 46.6, 42.5, 38.3, 27.8, 26.0, 23.1, 22.8

FTIR ν_{max} (ATR, film)/cm^{-1} 2953, 2930, 2868, 1687, 1655, 1513, 1463, 1452.

HRMS (+APCI) Calcd for $C_{19}H_{26}NO_3$ $[M+H]^+$ 316.1907, found: 316.1911.

Syntheses of the aza-Nazarov products 19:

General procedure B for the aza-Nazarov cyclization:

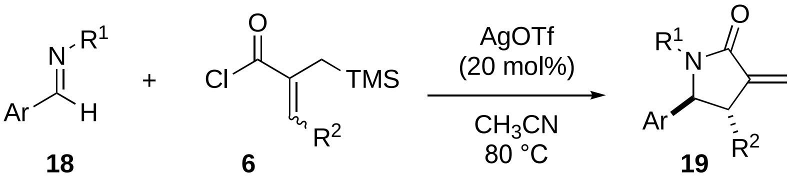

For the preparation of imine **18**, the corresponding amine was added to a mixture of the corresponding aldehyde on anhydrous Na_2SO_4 (750 mg) in 5.0 mL of anhydrous CH_2Cl_2. The reaction mixture was stirred at 23 °C for 16 h and filtered to give the imine product **18**, which was observed to be pure by ^{1}H NMR spectroscopy. A 25-mL, flame-dried Schlenk flask was cooled to ambient temperature under an inert atmosphere of nitrogen and charged with the

imine reactant **18** and 0.5 mL of anhydrous CH_3CN. A solution of acyl chloride **6** in 0.5 mL of anhydrous CH_3CN was added, and the resulting clear solution was stirred for 5 min at ambient temperature. Then, AgOTf was added. The flask was inserted into a preheated oil bath at 80 °C, and the reaction mixture was stirred at this temperature for 22 h. It was then cooled to ambient temperature and quenched with saturated $NaHCO_{3(aq)}$ solution (3 mL). The aqueous phase was extracted with EtOAc (3 × 10 mL). The combined organic layers were dried over Na_2SO_4, filtered, and concentrated under reduced pressure. Purification of the product was carried out by flash column chromatography using SiO_2 as the stationary phase.

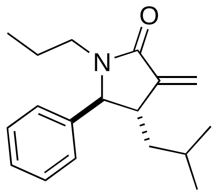

19a

Aza-Nazarov product **19a** was prepared according to the general procedure B using the corresponding imine reactant (58.9 mg, 0.40 mmol), acyl chloride **6b** (121 mg, 0.52 mmol), AgOTf (20.6 mg, 0.080 mmol), and 1.0 mL of anhydrous CH_3CN. Purification by flash column chromatography (EtOAc/hexanes 1:5) gave pure aza-Nazarov product **19a** as a yellow oil (50.1 mg, 46 % yield) in 10:1 dr.

R_f = 0.56 (EtOAc:hexanes = 1:2)

^{1}H NMR (400 MHz; $CDCl_3$) δ: 7.34-7.28 (3H, m), 7.16-7.13 (2H, m), 6.08 (1H, dd, *J* = 2.3, 0.4 Hz), 5.29 (1H, dd, *J* = 2.0, 0.6 Hz), 4.19 (1H, d, *J* = 2.8 Hz), 3.67 (1H, ddd, *J* = 13.6, 8.4, 7.6 Hz), 2.80-2.75 (1H, m), 2.59 (1H, ddd, 13.6, 8.4, 5.1 Hz), 1.83-1.73 (1H, m), 1.58-1.40 (4H, m), 0.90 (3H, d, *J* = 6.7 Hz), 0.83 (3H, t, *J* = 7.4 Hz), 0.76 (3H, d, *J* = 6.6 Hz).

^{13}C{^{1}H} NMR (100 MHz; $CDCl_3$) δ; **Major diastereomer**: 168.1, 144.4, 141.1, 129.1, 128.2, 126.7, 115.9, 66.3, 45.6, 44.5, 42.7, 25.2, 23.1, 22.1, 20.3, 11.4.

Minor diastereomer: 168.9, 144.7, 133.0, 128.6, 128.4, 116.5, 114.3, 64.0, 43.1, 41.4, 40.6, 25.4, 23.3, 22.6, 20.6, 11.5.

FTIR ν_{max} (ATR, film)/cm^{-1} 2959, 2932, 2871, 1691, 1658, 1457, 1421.

HRMS (+APCI) Calcd for $C_{18}H_{26}NO$ $[M+H]^+$ 272.2009, found: 272.2023.

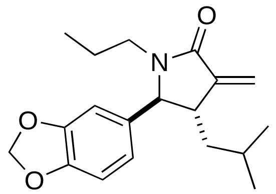

19d

Aza-Nazarov product **19d** was prepared according to the general procedure B using the corresponding imine reactant (54.4 mg, 0.28 mmol), acyl chloride **6b** (99 mg, 0.43 mmol), AgOTf (14.6 mg, 0.057 mmol), and 1.0 mL of anhydrous CH_3CN. Purification by flash column chromatography (EtOAc/hexanes 1:4) gave pure aza-Nazarov product **19d** as a yellow oil (63.4 mg, 69% yield) in 4.3:1 dr.

$\boldsymbol{R_f}$ = 0.28 (EtOAc:hexanes = 1:4)

^{1}H NMR (400 MHz; $CDCl_3$) δ: 6.73 (1H, d, *J* = 7.9 Hz), 6.62 (1H, dd, *J* = 7.9, 1.3 Hz), 6.59 (1H, s), 6.05 (1H, d, *J* = 2.1 Hz), 5.93 (2H, s), 5.27 (1H, d, *J* = 1.4 Hz), 4.09 (1H, d, *J* = 2.7 Hz), 3.68-3.59 (1H, m), 2.72 (1H, ddd, *J* = 8.5, 6.5, 2.3 Hz), 2.59 (1H, ddd, *J* = 13.6, 8.2, 5.3 Hz), 1.79-1.69 (1H, m), 1.56-1.34 (4H, m), 0.88 (3H, d, *J* = 6.5 Hz), 0.82 (3H, t, *J* = 7.4 Hz), 0.76 (3H, d, *J* = 6.5 Hz).

^{13}C{^{1}H} NMR (100 MHz; $CDCl_3$) δ; **Major diastereomer**: 167.9, 148.5, 147.6, 144.4, 134.9, 120.3, 115.8, 108.4, 106.6, 101.3, 66.2, 45.5, 44.4, 42.5, 25.1, 23.0, 22.1, 20.3, 11.4

Minor diastereomer: 168.6, 148.1, 147.6, 144.6, 131.4, 114.3, 108.1, 101.3, 63.8, 42.9, 40.5, 37.6, 25.5, 23.3, 22.0, 20.6, 11.5

FTIR ν_{max} (ATR, film)/cm^{-1} 2958, 2929, 2872, 1688, 1657, 1503, 1445, 1422.

HRMS (+ESI) Calcd for $C_{19}H_{25}NO_3$ $[M+H]^+$ 316.1907, found: 316.1907.

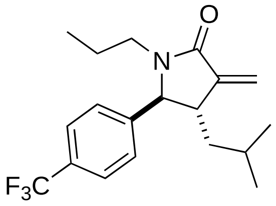

19e

Aza-Nazarov product **19e** was prepared according to the general procedure B using the corresponding imine reactant (63.1 mg, 0.29 mmol), acyl chloride **6b** (102 mg, 0.44 mmol), AgOTf (15.1 mg, 0.059 mmol), and 1.0 mL of anhydrous CH_3CN. Purification by flash column chromatography (EtOAc/hexanes 1:5) gave pure aza-Nazarov product **19e** as a pale yellow oil

(74.2 mg, 76% yield, major diastereomer) and **19e'** as a pale yellow oil (8.3 mg, 8% yield, minor diastereomer).

Major diastereomer, 19e:

***R**$_f$* = 0.29 (EtOAc:hexanes = 1:5)

^{1}H NMR (400 MHz; CDCl$_3$) δ: 7.61 (2H, d, *J* = 8.1 Hz), 7.27 (2H, d, *J* = 8.2 Hz), 6.11 (1H, d, *J* = 2.1 Hz), 5.32 (1H, d, *J* = 1.7 Hz), 4.27 (1H, d, *J* = 8.1 Hz), 3.71 (1H, dt, *J* = 13.8, 8.0 Hz), 2.76-2.71 (1H, m), 2.57 (1H, ddd, *J* = 13.6, 8.4, 5.1 Hz), 1.83-1.73 (1H, m), 1.59-1.39 (4H, m), 0.91 (3H, d, *J* = 6.7 Hz), 0.85 (3H, t, *J* = 7.4 Hz), 0.79 (3H, d, *J* = 6.6 Hz).

^{13}C{^{1}H} NMR (100 MHz; CDCl$_3$) δ: 168.1, 145.3, 143.6, 130.6 (q, $J_{C\text{-}F}$ = 32.7 Hz), 127.0, 126.2 (q, $J_{C\text{-}F}$ = 3.6 Hz), 124.1 (q, $J_{C\text{-}F}$ = 272.0 Hz), 116.7, 65.8, 45.7, 44.6, 42.9, 25.2, 23.0, 22.1, 20.4, 11.4

FTIR ν_{max} (ATR, film)/cm^{-1} 2960, 2933, 2873, 1692, 1659, 1620, 1416, 1324.

HRMS (+ESI) Calcd for $C_{19}H_{24}F_3NO$ [M+H]$^+$ 340.1883, found: 340.1883.

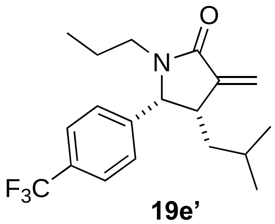

19e'

Minor diastereomer, 19e':

***R**$_f$* = 0.24 (EtOAc:hexanes = 1:5)

^{1}H NMR (400 MHz; CDCl$_3$) δ: 7.58 (2H, d, *J* = 8.2 Hz), 7.18 (2H, d, *J* = 7.4 Hz), 6.11 (1H, d, *J* = 3.3 Hz), 5.29 (1H, d, *J* = 8.0 Hz), 4.71 (1H, d, *J* = 8.0 Hz), 3.71 (1H, dt, *J* = 14.0, 8.0 Hz), 3.30-3.21 (1H, m), 2.55 (1H, ddd, *J* = 13.7, 8.5, 5.2 Hz), 1.57-1.42 (4H, m), 1.24-1.20 (1H, m), 0.90 (3H, d, *J* = 6.7 Hz), 0.87 (3H, t, *J* = 7.5 Hz), 0.67 (3H, d, *J* = 6.6 Hz).

^{13}C{^{1}H} NMR (100 MHz; CDCl$_3$) δ: 168.9, 144.0, 142.0, 130.8 (q, $J_{C\text{-}F}$ = 32.5 Hz), 128.3, 125.7 (q, $J_{C\text{-}F}$ = 3.7 Hz), 124.1 (q, $J_{C\text{-}F}$ = 272.1 Hz), 115.1, 63.6, 43.2, 40.6, 37.6, 25.6, 23.3, 22.1, 20.7, 11.5.

HRMS (+ESI) Calcd for $C_{19}H_{24}F_3NO$ [M+H]$^+$ 340.1883, found: 340.1865.

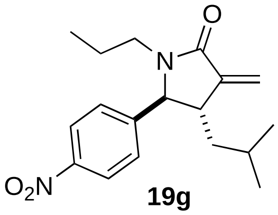

19g

Aza-Nazarov product **19g** was prepared according to the general procedure B using the corresponding imine reactant (76.9 mg, 0.40 mmol), acyl chloride **6b** (121 mg, 0.52 mmol), AgOTf (20.6 mg, 0.080 mmol), and 1.0 mL of anhydrous CH_3CN. Purification by flash column chromatography (EtOAc/hexanes 1:2) gave pure aza-Nazarov product **19g** as an orange oil (48.9 mg, 39% yield) in 5:1 dr.

$\boldsymbol{R_f}$ = 0.57 (EtOAc:hexanes = 1:2)
^{1}H NMR (400 MHz; $CDCl_3$) δ: 8.22-8.18 (2H, m), 7.35-7.31 (2H, m), 6.11 (1H, d, *J* = 2.1 Hz), 5.33 (1H, d, *J* = 1.8 Hz), 4.32 (1H, d, *J* = 2.6 Hz), 3.70 (1H, ddd, *J* = 13.9, 8.4, 7.5 Hz), 2.75-2.70 (1H, m), 2.57 (1H, ddd, 13.7, 8.5, 5.2 Hz), 1.80-1.70 (1H, m), 1.59-1.39 (4H, m), 0.90 (3H, d, *J* = 6.6 Hz), 0.84 (3H, t, *J* = 7.4 Hz), 0.78 (3H, d, *J* = 6.6 Hz).
^{13}C{^{1}H} NMR (100 MHz; $CDCl_3$) δ; **Major diastereomer**: 168.0, 148.6, 143.1, 127.4, 124.5, 117.1, 65.6, 45.6, 44.5, 43.0, 25.2, 22.9, 22.1, 20.4, 11.4
Minor diastereomer: 168.7, 147.9, 143.5, 128.9, 123.9, 115.4, 63.3, 43.3, 40.6, 37.6, 25.5, 23.2, 22.0, 20.7, 11.5.
FTIR ν_{max} (ATR, film)/cm^{-1} 2959, 2932, 2872, 1690, 1659, 1522, 1345.
HRMS (+APCI) Calcd for $C_{18}H_{25}N_2O_3$ $[M+H]^+$ 317.1860, found: 317.1862.

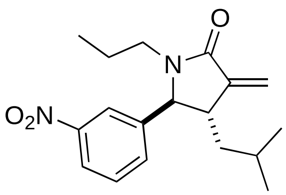

19h

Aza-Nazarov product **19h** was prepared according to the general procedure B using imine reactant (76.9 mg, 0.40 mmol), acyl chloride **6b** (112 mg, 0.48 mmol), AgOTf (20.6 mg, 0.080 mmol), and 1.0 mL of anhydrous CH_3CN. Purification by flash column chromatography (EtOAc/hexanes 1:2) gave pure aza-Nazarov product **19h** as a yellow oil (25.2 mg, 20% yield) in 12.5:1 dr.

$\boldsymbol{R_f}$ = 0.60 (EtOAc:hexanes = 1:2)
^{1}H NMR (400 MHz; $CDCl_3$) δ: 8.18 (1H, ddd, *J* = 8.0, 2.3, 1.2 Hz), 8.04 (1H, t, *J* = 2.0 Hz), 7.56 (1H, t, *J* = 7.9 Hz), 7.49 (1H, dt, *J* = 7.7, 1.4 Hz), 6.14 (1H, d, *J* = 2.2 Hz), 5.35 (1H, d, *J* = 2.0 Hz), 4.32 (1H, d, *J* = 2.6 Hz), 3.71 (1H, ddd, *J* = 13.8, 8.5, 7.5 Hz), 2.78-2.73 (1H, m), 2.58

(1H, ddd, 13.7, 8.5, 5.1 Hz), 1.83-1.73 (1H, m), 1.61-1.41 (4H, m), 0.92 (3H, d, J = 6.6 Hz), 0.85 (3H, t, J = 7.4 Hz), 0.79 (3H, d, J = 6.6 Hz).

^{13}C{^{1}H} NMR (100 MHz; CDCl$_3$) δ: 168.0, 148.9, 143.6, 143.2, 132.4, 130.4, 123.4, 121.8, 117.1, 65.7, 45.6, 44.6, 42.9, 25.2, 22.9, 22.1, 20.4, 11.4

FTIR ν_{max} (ATR, film)/cm^{-1} 2960, 2931, 2872, 1692, 1659, 1532, 1420.

HRMS (+APCI) Calcd for $C_{18}H_{25}N_2O_3$ [M+H]$^+$ 317.1860, found: 317.1878.

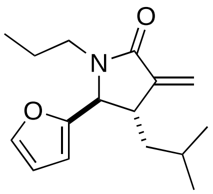

19i

Aza-Nazarov product **19i** was prepared according to the general procedure B using the corresponding imine reactant (54.9 mg, 0.40 mmol), acyl chloride **6b** (130 mg, 0.56 mmol), AgOTf (20.6 mg, 0.080 mmol), and 1.0 mL of anhydrous CH_3CN. Purification by flash column chromatography (EtOAc/hexanes 1:2) gave pure aza-Nazarov product **19i** as an orange oil (61.7 mg, 59% yield) in 11:1 dr.

R_f = 0.68 (EtOAc:hexanes = 1:2)

^{1}H NMR (400 MHz; CDCl$_3$) δ: 7.35 (1H, dd, J = 1.8, 0.8 Hz), 6.30 (1H, dd, J = 3.2, 1.8 Hz), 6.22 (1H, dd, J = 3.3, 0.6 Hz), 6.03 (1H, d, J = 2.5 Hz), 5.27 (1H, d, J = 2.1 Hz), 4.25 (1H, d, J = 3.7 Hz), 3.51 (1H, ddd, J = 13.7, 8.6, 7.1 Hz), 3.05-2.99 (1H, m), 2.78 (1H, ddd, J = 13.7, 8.6, 5.3 Hz), 1.74-1.64 (2H, m), 1.58-1.50 (1H, m), 1.43-1.27 (3H, m), 0.88 (3H, d, J = 6.4 Hz), 0.81 (3H, t, J = 7.4 Hz), 0.76 (3H, d, J = 6.5 Hz).

^{13}C{^{1}H} NMR (100 MHz; CDCl$_3$) δ; **Major diastereomer**: 167.6, 152.7, 144.1, 142.8, 115.4, 110.5, 108.2, 59.7, 44.7, 42.9, 40.7, 25.2, 23.2, 21.8, 20.4, 11.3.

Minor diastereomer: 168.6, 151.2, 144.0, 142.7, 116.4, 110.3, 109.2, 57.8, 43.2, 41.3, 40.3, 25.5, 23.5, 21.9, 20.6, 11.4.

FTIR ν_{max} (ATR, film)/cm^{-1} 2958, 2931, 2871, 1689, 1658, 1530, 1505, 1418.

HRMS (+APCI) Calcd for $C_{16}H_{24}NO_2$ [M+H]$^+$ 262.1802, found: 262.1804.

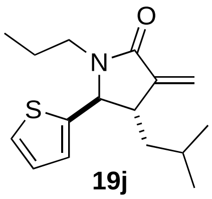

19j

Aza-Nazarov product **19j** was prepared according to the general procedure B using the corresponding imine reactant (61.3 mg, 0.40 mmol), acyl chloride **6b** (130 mg, 0.56 mmol), AgOTf (20.6 mg, 0.080 mmol), and 1.0 mL of anhydrous CH_3CN. Purification by flash column chromatography (EtOAc/hexanes 1:9 to 1:1) gave pure aza-Nazarov product **19j** as an orange oil (58.9 mg, 53% yield) in 6:1 dr.

$\boldsymbol{R_f}$ = 0.59 (EtOAc:hexanes = 1:2)

^{1}H NMR (400 MHz; $CDCl_3$) δ: 7.28-7.26 (1H, m), 6.97-6.94 (2H, m), 6.10 (1H, d, *J* = 2.3 Hz), 5.33 (1H, d, *J* = 2.1, 0.5 Hz), 4.50 (1H, d, *J* = 3.2 Hz), 3.66 (1H, ddd, *J* = 13.7, 8.6, 7.4 Hz), 2.96-2.90 (1H, m), 2.75 (1H, ddd, 13.7, 8.5, 5.1 Hz), 1.82-1.75 (1H, m), 1.60-1.45 (4H, m), 0.92 (3H, d, *J* = 6.5 Hz), 0.86 (3H, t, *J* = 7.4 Hz), 0.82 (3H, d, *J* = 6.5 Hz).

^{13}C{^{1}H} NMR (100 MHz; $CDCl_3$) δ; **Major diastereomer**: 167.4, 144.9, 143.83, 126.9, 125.7, 125.6, 116.1, 61.8, 45.2, 45.1, 42.6, 25.3, 23.1, 22.1, 20.4, 11.4.

Minor diastereomer: 168.3, 143.75, 126.7, 125.5, 116.5, 59.7, 43.0, 45.1, 41.2, 37.2, 25.7, 23.5, 22.0, 20.7, 11.5.

FTIR ν_{max} (ATR, film)/cm^{-1} 2957, 2928, 2870, 1688, 1658, 1532, 1417.

HRMS (+APCI) Calcd for $C_{16}H_{24}NOS$ $[M+H]^+$ 278.1574, found: 278.1579.

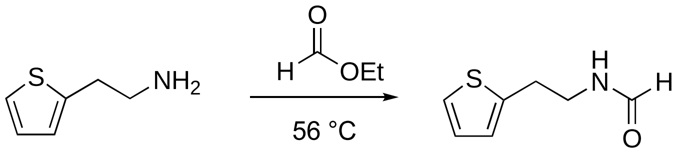

S1

Ethyl formate (5 mL) was added directly to thiophene-2-ethylamine (200 mg, 1.57 mmol) at 23 °C under N_2. The reaction mixture was placed in oil bath with a condenser to reflux at 56 °C. The product formation was followed by TLC analysis. After stirring the reaction mixture for 24 h, ethyl formate was directly evaporated under reduced pressure. The residue was purified by flash column chromatography (only EtOAc) to afford compound **S1** was as pale yellow oil (236 mg, 97% yield). The ^{1}H NMR and ^{13}C{^{1}H} NMR spectral data match those reported in the literature.[3]

$\boldsymbol{R_f}$ = 0.43 (EtOAc)

^{1}H NMR (400 MHz; $CDCl_3$) δ: 8.11 (1H, s), 7.15 (1H, d, J = 5.1 Hz), 6.93 (1H, dd, J = 5.1, 3.6 Hz), 6.84 (1H, d, J = 2.4 Hz), 6.02 (1H, br s), 3.56 (2H, q, J = 6.5 Hz), 3.04 (2H, t, J = 6.7 Hz).

^{13}C{^{1}H} NMR (100 MHz; $CDCl_3$) δ; Major rotamer: 161.4, 141.0, 127.2, 125.6, 124.1, 39.5, 29.9.

Minor rotamer: 164.6, 139.8, 127.3, 126.0, 124.5, 43.3, 31.9

FTIR ν_{max} (ATR, film)/cm^{-1} 3282, 3065, 2930, 2863, 1660, 1532, 1437, 1384, 1245.

HRMS (+ESI) Calcd for $C_7H_{10}NOS$ $[M+H]^+$ 156.0478, found: 156.0470.

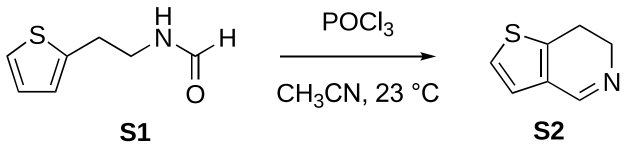

To a solution of **S1** (434 mg, 2.78 mmol) in CH_3CN (2 mL), $POCl_3$ (417 µL, 4.47 mmol) was added slowly at 23 °C under N_2. The product formation was followed by TLC analysis. After stirring the reaction mixture for 24 h, the color of the reaction medium became green, and CH_3CN was directly evaporated under reduced pressure. The residue was dissolved in water (3 mL) by heating gently to dissolve the green residue completely, and saturated $Na_2CO_{3(aq9}$ solution was added slowly. H_2 gas formation was observed during the addition of saturated $Na_2CO_{3(aq)}$ solution, and the reaction medium became brown. The aqueous phase was extracted with CH_2Cl_2 (3 × 30 mL). The combined organic layers were dried over Na_2SO_4, filtered, and concentrated under reduced pressure. The residue was purified by flash column chromatography (EtOAc/MeOH 9:1) to afford compound **S2** as a brownish solid (77 mg, 21% yield). While the synthesis of compound **S2** was reported in the literature, it was characterized only by elemental analysis and UV–vis spectroscopy.[4]

Mp: 102.6 – 105.2 °C

$\boldsymbol{R_f}$ = 0.23 (EtOAc:MeOH = 9:1)

^{1}H NMR (400 MHz; $CDCl_3$) δ: 8.29 (1H, t, J = 2.1 Hz), 7.09 (1H, d, J = 5.1 Hz), 7.02 (1H, d, J = 5.1 Hz), 3.85-3.81 (2H, m), 2.89-2.85 (2H, m)

^{13}C{^{1}H} NMR (100 MHz; $CDCl_3$) δ: 155.1, 142.6, 131.2, 124.7, 123.0, 47.9, 22.2.

FTIR ν_{max} (ATR, film)/cm^{-1} 3200, 3086, 2875, 2856, 1675, 1530, 1390, 1384, 1248.

HRMS (+ESI) Calcd for C_7H_8NS $[M+H]^+$ 138.0372, found: 138.0372.

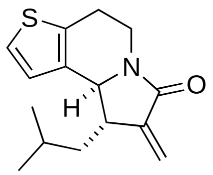

19k

The synthesis of compound **19k** was carried out using imine **S2** (20 mg, 0.15 mmol) and acyl chloride **6b** (44 mg, 0.19 mmol) with AgOTf (44.9 mg, 0.175 mmol) in 1 mL of anhydrous CH_2Cl_2. Purification by column chromatography (EtOAc/hexanes 1:1) provided aza-Nazarov product **19k** as a pale yellow solid (15.3 mg, 40% yield) as a single diastereomer.

Mp: 156.2 – 158.7 °C

$\boldsymbol{R_f}$ = 0.56 (1:1, EtOAc: hexanes)

^{1}H NMR (400 MHz; $CDCl_3$) δ: 7.17 (1H, d, *J* = 5.2 Hz), 6.84 (1H, d, *J* = 5.2 Hz), 6.05 (1H, d, *J* = 2.7 Hz), 5.32 (1H, d, *J* = 2.3 Hz), 4.61-4.56 (1H, m), 4.37-4.35 (1H, m), 3.11 (1H, ddd, *J* = 12.6, 11.6, 4.8 Hz), 3.04-2.95 (1H, m), 2.88-2.79 (2H, m), 1.92 (1H, sept, *J* = 6.6 Hz), 1.72-1.28 (3H, m), 1.08 (3H, d, *J* = 6.5 Hz), 1.03 (3H, d, *J* = 6.6 Hz).

^{13}C{^{1}H} NMR (100 MHz; $CDCl_3$) δ: 167.1, 144.9, 135.9, 134.2, 124.1, 123.7, 116.3, 60.8, 45.6, 42.1, 38.1, 26.1, 24.6, 23.1, 22.9.

FTIR ν_{max} (ATR, film)/cm^{-1} 2954, 2924, 2868, 1694, 1657, 1452, 1420, 1326.

HRMS (+ESI) Calcd for $C_{15}H_{20}NOS$ $[M+H]^+$ 262.1261, found: 262.1259.

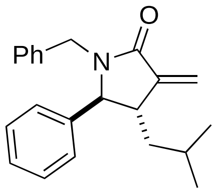

19l

Aza-Nazarov product **19l** was prepared according to the general procedure B using the corresponding imine reactant (78.1 mg, 0.40 mmol), acyl chloride **6b** (121 mg, 0.52 mmol), AgOTf (20.6 mg, 0.080 mmol), and 1.0 mL of anhydrous CH_3CN. Purification by flash column chromatography (EtOAc/hexanes 1:5) gave pure aza-Nazarov product **19l** as a white solid (62.7 mg, 49% yield) in 10:1 dr.

$\boldsymbol{R_f}$ = 0.65 (EtOAc:hexanes = 1:2)

^{1}H NMR (400 MHz; $CDCl_3$) δ: 7.37-7.26 (6H, m), 7.12-7.09 (4H, m), 6.17 (1H, d, *J* = 2.3 Hz), 5.36 (1H, d, *J* = 2.0 Hz), 5.18 (1H, d, *J* = 14.7 Hz), 3.97 (1H, d, *J* = 3.0 Hz), 3.50 (1H, d, *J*

= 14.7 Hz), 2.83-2.78 (1H, m), 1.68-1.61 (1H, m), 1.42-1.36 (2H, m), 0.82 (3H, d, *J* = 6.6 Hz), 0.69 (3H, d, *J* = 6.6 Hz).

^{13}C{^{1}H} NMR (100 MHz; $CDCl_3$) δ; Major diastereomer: 168.0, 144.3, 140.6, 136.3, 129.1, 128.7, 128.6, 128.3, 127.7, 127.0, 116.4, 65.3, 45.2, 44.7, 44.3, 25.1, 23.0, 22.0.

Minor diastereomer: 168.7, 144.5, 137.1, 136.5, 128.8, 128.7, 128.4, 127.7, 115.1, 63.0, 45.0, 40.3, 37.8, 25.4, 23.3.

FTIR ν_{max} (ATR, film)/cm^{-1} 2955, 2927, 2869, 1693, 1659, 1419.

HRMS (+APCI) Calcd for $C_{22}H_{26}NO$ $[M+H]^+$ 320.2009, found: 320.2009.

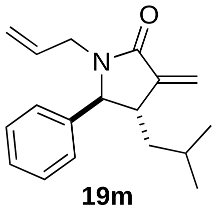

19m

Aza-Nazarov product **19m** was prepared according to the general procedure B using the corresponding imine reactant (33.3 mg, 0.23 mmol), acyl chloride **6b** (80.2 mg, 0.34 mmol), AgOTf (11.8 mg, 0.046 mmol), and 1.0 mL of anhydrous CH_3CN. Purification by flash column chromatography (EtOAc/hexanes 1:5) gave pure aza-Nazarov product **19m** as a pale yellow oil (34.6 mg, 56% yield) in 16:1 dr.

R_f = 0.26 (EtOAc:hexanes = 1:5)

^{1}H NMR (400 MHz; $CDCl_3$) δ: 7.36-7.30 (3H, m), 7.15-7.13 (2H, m), 6.12 (1H, d, *J* = 2.3 Hz), 5.67 (1H, dddd, *J* = 17.8, 10.1, 7.9, 4.5 Hz), 5.33 (1H, d, *J* = 1.9 Hz), 5.15 (1H, d, *J* = 10.2 Hz), 5.02 (1H, dd, *J* = 17.1, 1.0 Hz), 4.50-4.45 (1H, m), 4.20 (1H, d, *J* = 2.9 Hz), 3.08 (1H, dd, *J* = 15.2, 7.9 Hz), 2.81 (1H, tt, 8.6, 2.3 Hz), 1.77 (1H, qd, *J* = 13.1, 6.5 Hz), 1.58-1.51 (1H, m), 1.48-1.41 (1H, m), 0.90 (3H, d, *J* = 6.6 Hz), 0.75 (3H, d, *J* = 6.6 Hz).

^{13}C{^{1}H} NMR (100 MHz; $CDCl_3$) δ: 167.8, 144.3, 140.8, 132.1, 129.1, 128.3, 126.9, 118.5, 116.3, 65.8, 45.5, 44.4, 43.6, 25.2, 23.1, 22.1

FTIR ν_{max} (ATR, film)/cm^{-1} 2956, 2927, 2870, 1695, 1659, 1413, 1283.

HRMS (+ESI) Calcd for $C_{18}H_{23}NO$ $[M+H]^+$ 270.1852, found: 270.1848.

List of unsuccessful substrates in the aza-Nazarov reaction:

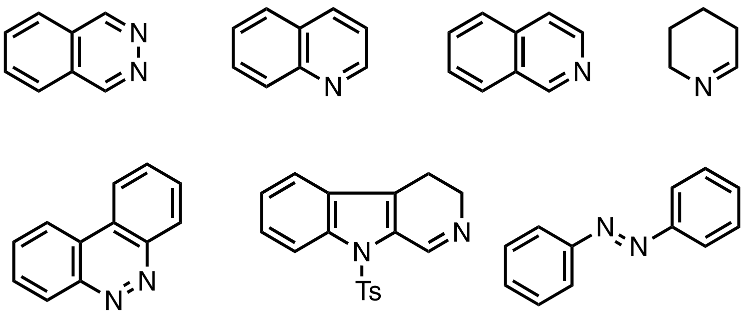

Preparation of acyl chloride 23:

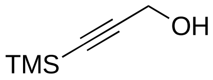

25

Compound **25** was prepared following a literature procedure.[5] A solution of propargyl alcohol (320 mg, 5.64 mmol, 0.30 mL) in anhydrous THF (15 mL) was cooled to −78 °C, and *n*-BuLi (1.6 M in hexanes, 8.80 mL, 14.1 mmol) was added dropwise. After stirring for 30 min at this temperature, TMSCl (1.80 mL, 14.1 mmol) was added carefully. After stirring the reaction mixture at this temperature for 5 min, it was allowed to warm up to ambient temperature and stirred for 1.5 h. To the reaction mixture, 16 mL of water and 10% $HCl_{(aq)}$ solution were added, and it was stirred until complete hydrolysis of the TMS ether. The aqueous phase was then extracted three times with CH_2Cl_2, and the combined organic phase was washed with brine. It was then dried over anhydrous Na_2SO_4, filtered, and concentrated under vacuum. Purification by flash column chromatography (CH_2Cl_2/pentane 1:4) gave pure product **25** as a yellow oil (649 mg, 89% yield). The spectral data match those reported in the literature.[3]

Note: Since the product is volatile, it should be handled with care.

^{1}H NMR (400 MHz; $CDCl_3$) δ: 4.27 (2H, s), 1.67 (1H, br s), 0.18 (9H, s).

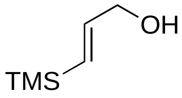

26

Compound **26** was prepared following a literature procedure.[6] To a suspension of $LiAlH_4$ (74 mg, 1.95 mmol) in 2 mL of DME at 0 °C was added a solution of alkyne **25** (239 mg, 1.86 mmol) in 3 mL of DME under an inert atmosphere of N_2. The reaction mixture was stirred at 23 °C for 24 h. TLC analysis indicated full consumption of the starting material **25**. The reaction mixture was quenched with 3 mL of water and diluted with 1 M $HCl_{(aq)}$ (40 mL). The aqueous phase was extracted with EtOAc. The organic phase was dried over anhydrous Na_2SO_4, filtered, and concentrated under vacuum. The product was used in the next step without further purification.

^{1}H NMR (400 MHz; $CDCl_3$) δ: 6.18 (1H, dt, J = 18.8, 4.4 Hz), 5.92 (1H, dt, J = 18.8, 1.7 Hz), 4.18 (2H, dd, J = 4.4, 1.7 Hz), 0.08 (9H, s).

^{13}C{^{1}H} NMR (100 MHz; $CDCl_3$) δ: 144.9, 129.7, 65.7, -1.22.

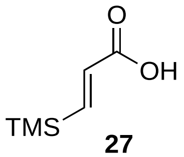

27

Compound **27** was prepared following a literature procedure.[7] To a stirred solution of allylic alcohol **26** (142 mg, 1.09 mmol) in 1.4 mL of acetone was added a solution of CrO_3 (291 mg, 2.91 mmol) in concentrated $H_2SO_{4(aq)}$ (0.35 mL) and H_2O (1.8 mL) at 0 °C. The reaction mixture was stirred at 0 °C for 6 h and the warmed up to ambient temperature. The reaction mixture was diluted with H_2O, and the aqueous phase was extracted with CH_2Cl_2. The combined organic phase was then dried over anhydrous Na_2SO_4, filtered, and concentrated under vacuum. Purification by flash column chromatography (EtOAc/hexanes 1:5) gave pure product **27** as a pale yellow solid (132 mg, 84% yield over two steps). The spectral data match those reported in the literature.[5]

Mp: 38.7 – 39.9 °C

^{1}H NMR (400 MHz; $CDCl_3$) δ: 11.66 (1H, br s), 7.39 (1H, d, J = 18.9 Hz), 6.25 (1H, d, J = 18.9 Hz), 0.15 (9H, s).

^{13}C{^{1}H} NMR (100 MHz; $CDCl_3$) δ: 171.4, 153.1, 133.4, -1.87.

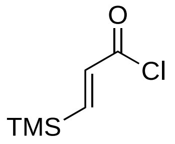

23

Compound **23** was prepared following a literature procedure.[8] To a solution of carboxylic acid **27** (81.5 mg, 0.58 mmol) in 1.0 mL of CH_2Cl_2 was added oxalyl chloride (0.10 mL, 1.16 mmol). The reaction mixture was stirred at 40 °C for 2 h. Afterward, the reaction mixture was cooled to ambient temperature, and all volatiles were removed under vacuum to afford pure acyl chloride **23** (83.4 mg, 91%).

Note: This product was used immediately after its preparation due to its sensitivity toward moisture.

^{1}H NMR (400 MHz; CDCl$_3$) δ: 7.51 (1H, d, *J* = 18.3 Hz), 6.39 (1H, d, *J* = 18.3 Hz), 0.19 (9H, s).

Table S1: Investigation of the aza-Nazarov reaction with acyl chloride **23**.

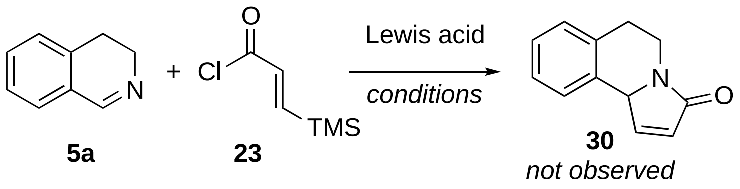

entry	Lewis acid (equiv)	solvent	*T* (°C)
1	AgOTf (1.2)	CH_3CN	23
2	AgOTf (0.2)	CH_3CN	80
3	AgOTf (1.2)	CH_3CN	80
4	AgOTf (1.2)	CH_2Cl_2	23
5	$BF_3·OEt_2$ (0.2)	CH_3CN	80

Isolation of aldehydes 32 and 34:

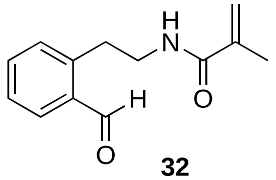

32

To a mixture of **5a** (200 mg, 1.53 mmol) in CH_2Cl_2 (2 mL) and saturated aqueous solution of $NaHCO_3$ (2 mL), methacryloyl chloride (**31**, 186 µL, 1.91 mmol) in CH_2Cl_2 (2 mL) was added at 23 °C. After stirring the reaction mixture for 1 h, the reaction was quenched with 10% aqueous ammonia solution (10 mL) solution and brine (10 mL). The aqueous phase was extracted with EtOAc (3 × 30 mL). The combined organic layer was dried over Na_2SO_4, filtered, and concentrated under reduced pressure. The residue was purified by flash column chromatography twice: first attempt (EtOAc/hexanes 1:1, ***Rf*** = 0.44) gave a colorless oil. Further purification (toluene/acetone 2:1) provided compound **32** as a pure white solid.

Mp: 70.2 – 72.6 °C

$\boldsymbol{R_f}$ = 0.56 (toluene:acetone = 2:1)

^{1}H NMR (400 MHz; $CDCl_3$) δ: 10.16 (1H, s), 7.80 (1H, dd, *J* = 7.6, 1.4 Hz), 7.54 (1H, td, *J* = 7.5, 1.2 Hz), 7.44 (1H, td, *J* = 7.5, 1.2 Hz), 7.33 (1H, d, *J* = 7.6 Hz), 6.22 (1H, bs, *NH*), 5.62 (1H, app t, *J* = 0.8 Hz), 5.28 (1H, app quint, *J* = 1.5 Hz), 3.58 (2H, q, *J* = 6.9 Hz), 3.28 (2H, t, *J* = 6.9 Hz), 1.91 (3H, dd, *J* = 1.4, 0.9 Hz).

^{13}C{^{1}H} NMR (100 MHz; $CDCl_3$) δ: 193.9, 168.6, 141.5, 140.1, 134.44, 134.36, 134.1, 132.1, 127.4, 119.6, 41.4, 32.4, 18.7

FTIR ν_{max} (ATR, film)/cm^{-1} 3331 (br), 2924, 2854, 1693, 1655, 1615, 1600, 1526, 1452, 1432.

HRMS (+APCI) Calcd for $C_{13}H_{16}NO_2$ $[M+H]^+$ 218.1176, found: 218.1191.

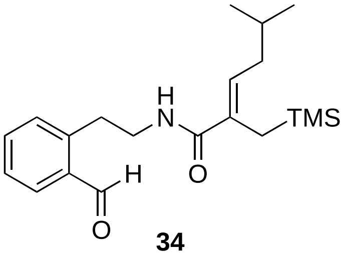

34

To a mixture of **5a** (59 mg, 0.45 mmol) in CH_2Cl_2 (2 mL) and saturated aqueous solution of $NaHCO_3$ (2 mL), acyl chloride **6b** (130 mg, 0.56 mmol) in CH_2Cl_2 (2 mL) was added at 23 °C. After stirring the reaction mixture for 1 h, the reaction was quenched with 10% aqueous

ammonia solution (10 mL) solution and brine (10 mL). The aqueous phase was extracted with EtOAc (3 × 30 mL). The combined organic layer was dried over Na_2SO_4, filtered, and concentrated under reduced pressure. The residue was purified by flash column chromatography twice: first attempt (EtOAc/hexanes 1:2, ***Rf*** = 0.38) gave a colorless oil. Further purification (toluene/acetone 9:1) provided compound **34** as a pure white solid.

Mp: 93.8 – 96.0 °C

$\boldsymbol{R_f}$ = 0.40 (toluene:acetone = 9:1)

^{1}H NMR (400 MHz; $CDCl_3$) δ: 10.18 (1H, s), 7.80 (1H, dd, *J* = 7.6, 1.5 Hz), 7.54 (1H, td, *J* = 7.5, 1.5 Hz), 7.44 (1H, td, *J* = 7.5, 1.3 Hz), 7.35 (1H, d, *J* = 7.5 Hz), 6.09 (1H, app t, *J* = 5.7 Hz, *NH*), 5.89 (1H, t, *J* = 7.0 Hz), 3.56 (2H, q, *J* = 6.8 Hz), 3.28 (2H, t, *J* = 7.1 Hz), 1.92 (2H, t, *J* = 6.9 Hz), 1.79 (2H, s), 1.73-1.63 (1H, m), 0.91 (6H, d, *J* = 6.7 Hz), -0.04 (9H, s).

^{13}C{^{1}H} NMR (100 MHz; $CDCl_3$) δ: 193.8, 170.6, 141.7, 135.8, 134.5, 134.3, 134.1, 132.1, 129.6, 127.3, 41.4, 37.9, 32.6, 28.6, 22.7, 18.2, -1.0.

FTIR ν_{max} (ATR, film)/cm^{-1} 3339 (br), 2954, 2897, 2870, 1695, 1650, 1615, 1601, 1525, 1428.

HRMS (+APCI) Calcd for $C_{20}H_{32}NO_2Si$ $[M+H]^+$ 346.2197, found: 346.2209.

Table S2: Investigation of the aza-Hosomi–Sakurai reaction between imine **5a** and ester **36**.

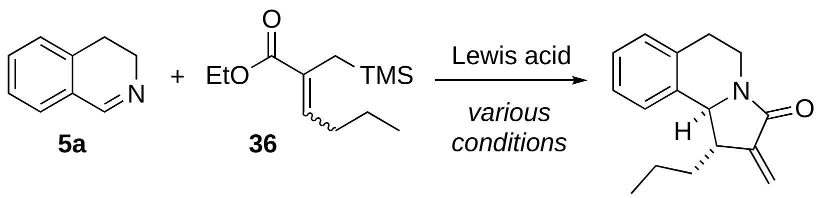

7
not observed

entry	Lewis acid (20 mol%)	solvent	*T* (°C)
1	$Zn(OTf)_2$	CH_3CN	70
2	$ZnBr_2$	CH_3CN	70
3	$CuCl_2$	CH_3CN	70
4	Tf_2NH	CH_3CN	70
5	$BF_3 \cdot OEt_2$	CH_2Cl_2	23
6	AgOTf	CH_3CN	80

Synthesis of acyl chlorides 6ba and 6bb:

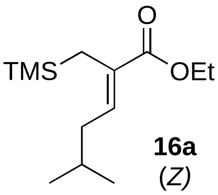

16a
(*Z*)

Purification of **16** (dr = 3:1) by flash column chromatography (hexanes/$PhCF_3$ 10:1) provided **16a** as a pure (*Z)* diastereomer.

R

$\boldsymbol{R_f}$ = 0.68 (hexanes:$PhCF_3$ = 10:1)

TLC Visualization: UV active; stains with $KMnO_4$ solution.

^{1}H NMR (400 MHz; $CDCl_3$) δ: 6.62 (1H, t, *J* = 7.3 Hz), 4.15 (2H, q, *J* = 7.2 Hz), 1.96 (2H, t, *J* = 7.1 Hz), 1.78 (2H, s), 1.70 (1H, sept, *J* = 6.7 Hz), 1.27 (3H, t, *J* = 7.1 Hz), 0.91 (6H, d, *J* = 6.7 Hz), -0.02 (9H, s).

^{13}C{^{1}H} NMR (100 MHz; $CDCl_3$) δ: 168.4, 137.5, 130.7, 60.4, 38.3, 28.5, 22.7, 17.4, 14.4, -0.9.

FTIR ν_{max} (ATR, film)/cm^{-1} 2956, 2902, 2872, 1710, 1636, 1465, 1368, 1330, 1283.

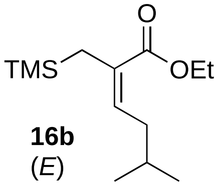

16b
(*E*)

Purification of **16** (dr = 3:1) by flash column chromatography (hexanes/$PhCF_3$ 10:1) provided **16b** as a pure (*E)* diastereomer.

$\boldsymbol{R_f}$ = 0.59 (hexanes: $PhCF_3$ = 10:1)

TLC Visualization: UV active; stains with $KMnO_4$ solution.

^{1}H NMR (400 MHz; $CDCl_3$) δ: 5.67 (1H, t, *J* = 7.6 Hz), 4.16 (2H, q, *J* = 7.2 Hz), 2.29 (2H, t, *J* = 7.2 Hz), 1.74 (2H, s), 1.66 (1H, sept, *J* = 6.7 Hz), 1.29 (3H, t, *J* = 7.1 Hz), 0.90 (6H, d, *J* = 6.7 Hz), -0.02 (9H, s).

^{13}C{^{1}H} NMR (100 MHz; $CDCl_3$) δ: 168.7, 137.9, 130.1, 60.1, 38.7, 24.4, 22.6, 19.9, 14.4, 1.48.

FTIR ν_{max} (ATR, film)/cm^{-1} 2958, 2901, 2869, 1711, 1636, 1478, 1368, 1279.

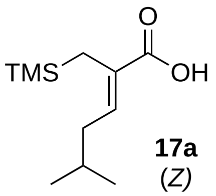

17a
(*Z*)

To a solution of **16a** (60 mg, 0.25 mmol) in 20% H_2O in EtOH (2 mL), KOH (42.3 mg, 0.75 mmol) was added as solid at 23 °C under N_2. The reaction mixture was placed in oil bath with a condenser and heated to reflux at 85 °C for 24 h. The reaction was quenched with 1.0 M $HCl_{(aq)}$ (3 mL) and saturated $NH_4Cl_{(aq)}$ solution. The aqueous phase was extracted with CH_2Cl_2 (3 × 30 mL). The combined organic layer was dried over Na_2SO_4, filtered, and concentrated under reduced pressure. The residue was purified by flash column chromatography (EtOAc/hexanes 1:5). Compound **17a** was obtained as pale orange solid (30 mg, 56% yield) as single (*Z*) diastereomer.

Note: 1.0 M $HCl_{(aq)}$ was directly added to the crude reaction mixture until pH 1–2.

$\boldsymbol{R_f}$ = 0.63 (EtOAc: hexanes = 1:5)

TLC Visualization: UV active; stains with $KMnO_4$ solution.

^{1}H NMR (400 MHz; $CDCl_3$) δ: 6.81 (1H, t, *J* = 7.3 Hz), 2.02 (2H, t, *J* = 7.0 Hz), 1.80 (2H, s), 1.78-1.70 (1H, m), 0.94 (6H, d, *J* = 6.7 Hz), 0.02 (9H, s).

^{13}C{^{1}H} NMR (100 MHz; $CDCl_3$) δ: 173.2, 140.5, 130.2, 38.5, 28.5, 22.7, 17.2, -0.8

FTIR ν_{max} (ATR, film)/cm^{-1} 2956, 2899, 2872, 1681, 1630, 1420, 1290, 1249.

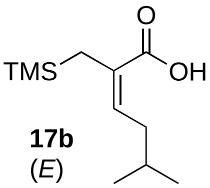

17b
(*E*)

To a solution of **16b** (40 mg, 0.16 mmol) in 20% H_2O in EtOH (2 mL), KOH (28.2 mg, 0.50 mmol) was added as solid at 23 °C under N_2. The reaction mixture was placed in oil bath with a condenser to reflux at 85 °C for 48 h. It was then quenched with 1.0 M $HCl_{(aq)}$ (3 mL) and saturated $NH_4Cl_{(aq)}$ solution. The aqueous phase was extracted with CH_2Cl_2 (3 × 30 mL). The combined organic layers were dried over Na_2SO_4, filtered, and concentrated under reduced pressure. The residue was purified by flash column chromatography (EtOAc/hexanes 1:5). Compound **17b** was obtained as pale orange oil (20 mg, 58% yield) as single (*E*) diastereomer.

Note: 1.0 M $HCl_{(aq)}$ was directly added to the crude reaction mixture until pH 1–2.

$\boldsymbol{R_f}$ = 0.55 (EtOAc:hexanes = 1:5)

TLC Visualization: UV active; stains with $KMnO_4$ solution.

^{1}H NMR (400 MHz; CDCl$_3$) δ: 5.86 (1H, t, *J* = 7.5 Hz), 2.40 (2H, t, *J* = 7.2 Hz), 1.77 (2H, s), 1.69 (1H, sept, *J* = 6.7 Hz), 0.93 (6H, d, *J* = 6.7 Hz), 0.01 (9H, s).

^{13}C{^{1}H} NMR (100 MHz; CDCl$_3$) δ: 174.1, 141.8, 129.0, 38.9, 29.2, 24.1, 22.6, 1.49.

FTIR ν_{max} (ATR, film)/cm^{-1} 2956, 2898, 2872, 1686, 1625, 1425, 1248.

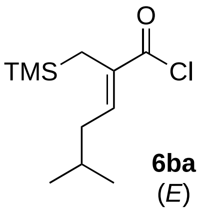

6ba
(*E*)

Oxalyl chloride (2 mL, 23.3 mmol) was directly added to **17a** (23.1 mg, 0.11 mmol) at 23 °C under N_2. The reaction mixture was placed in an oil bath at 60 °C, and stirred for 2 h. Then, excess amount of oxalyl chloride was carefully evaporated under reduced pressure by placing rotary evaporator inside well-ventilated fume hood. Compound **6ba** was obtained as pale orange oil (16.1 mg, 92% yield) as single (*E*) diastereomer.

Note: This product was used immediately used after its preparation due to its sensitivity toward moisture.

^{1}H NMR (400 MHz; CDCl$_3$) δ: 7.12 (1H, t, *J* = 7.2 Hz), 2.10 (2H, t, *J* = 7.1 Hz), 1.84 (2H, s), 1.84-1.79 (1H, m), 0.97 (6H, d, *J* = 6.6 Hz), 0.07 (9H, s).

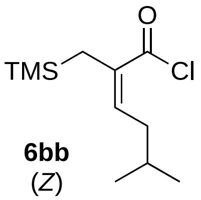

6bb
(*Z*)

Oxalyl chloride (2 mL, 23.3 mmol) was directly added to **17b** (10.1 mg, 0.05 mmol) at 23 °C under N_2. The reaction mixture was placed in an oil bath at 60 °C, and stirred for 2 h. Then, excess amount of oxalyl chloride was carefully removed under reduced pressure using a rotary evaporator placed inside a well-ventilated fume hood. Compound **6bb** was obtained as a pale orange oil (8.4 mg, 95% yield).

Note: This product was used immediately used after its preparation due to its sensitivity toward moisture.

^{1}H NMR (400 MHz; CDCl$_3$) δ: 5.74 (1H, t, *J* = 7.5 Hz), 2.21 (2H, t, *J* = 7.2 Hz), 1.93 (2H, s), 1.73-1.67 (1H, m), 0.92 (6H, d, *J* = 6.7 Hz), 0.00 (9H, s).

X-ray diffraction analysis of aza-Nazarov product 19l:

A suitable crystal of compound **19l** was selected for data collection which was performed on a Bruker diffractometer equipped with a graphite-monochromatic MoKα radiation at 296 K. We used these procedures for our analysis: solved by direct methods; SHELXS-2013;[9] refined by full-matrix least-squares methods; SHELXL-2013;[10] data collection: Bruker APEX2;[11] molecular graphics: MERCURY;[12] solution: WinGX.[13] Details of data collection and crystal structure determinations are given in Table S3.

Table S3. Crystal data and structure refinement parameters for **19l**.

Empirical formula	$C_{22}H_{25}NO$
Formula weight	319.43
Crystal system	Monoclinic
Space group	$P2_1$
a (Å)	10.2688 (12)
b (Å)	9.2623 (10)
c (Å)	11.0055 (13)
β (º)	115.655 (4)
V (Å^3)	943.57 (19)
Z	2
D_c (g cm^{-3})	1.124
μ (mm^{-1})	0.07
θ range (º)	2.3-25.9
Measured refls.	22679
Independent refls.	3700
R_{int}	0.080
S	0.95
R1/wR2	0.087/0.181
$\Delta\rho_{max}/\Delta\rho_{min}$ (eÅ^{-3})	0.39/-0.21
CCDC	2116978

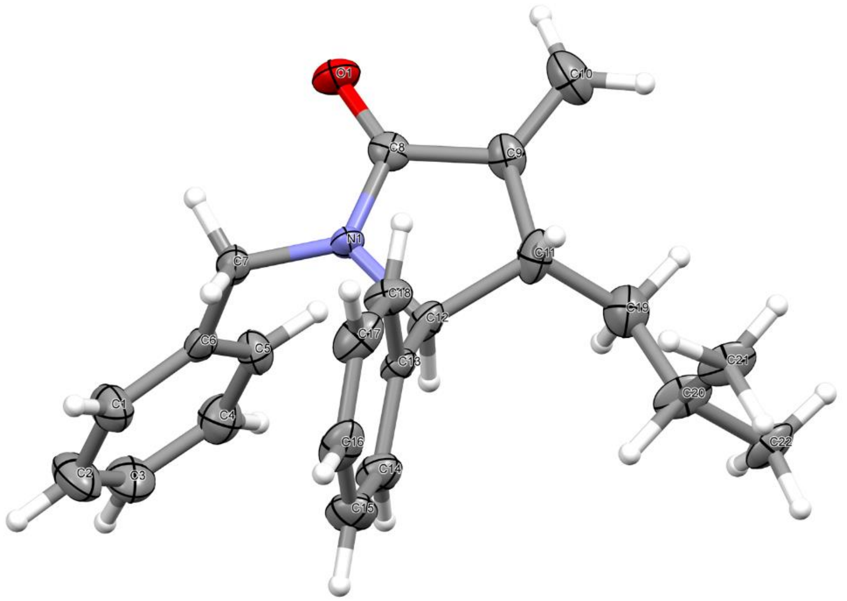

Figure S1: The molecular structure of **19l**, showing 50% probability displacement ellipsoids and the atomic numbering.

References

1. Donmez, S. E.; Soydaş, E.; Aydın, G.; Şahin, O.; Bozkaya, U.; Türkmen, Y. E. *Org. Lett.* **2019**, *21*, 554-558.
2. Hirose, T.; Sunazuka, T.; Shirahata, T.; Yamamoto, D.; Harigaya, Y.; Kuwajima, I.; Omura, S. *Org. Lett.* **2002**, *4*, 501-503.
3. Lv, F.; Li, Z.-F.; Hu, W.; Wu, X. *Bioorg. Med. Chem.* **2015**, *23*, 7661-7670.
4. Herz, W.; Tsai, L. *J. Am. Chem. Soc.* **1955**, *77*, 3529-3533.
5. Langille, N. F.; Jamison, T. F. *Org. Lett.* **2006**, *8*, 3761-3764.
6. Anzo, T.; Suzuki, A.; Sawamura, K.; Motozaki, T.; Hatta, M.; Takao, K.; Tadano, K. *Tetrahedron Lett.* **2007**, *48*, 8442-8448.
7. Fleming, I.; Gil, S.; Sarkar, A. K.; Schmidlin, T. *J. Chem. Soc. Perkin Trans. 1* **1992**, 3351-3361.
8. Wilson, S. R.; Di Grandi, M. J. *J. Org. Chem.* **1991**, *56*, 4766-4772.
9. Sheldrick, G.M. (2008). Acta Cryst. A64, 112.
10. Sheldrick, G. M. (2015). Acta Cryst. C71, 3.
11. APEX2, Bruker AXS Inc. Madison Wisconsin USA (2013).
12. Macrae, C. F.; Sovago, I.; Cottrell, S. J.; Galek, P. T. A.; McCabe, P.; Pidcock, E.; Platings, M.; Shields, G. P.; Stevens, J. S.; Towler, M.; Wood, P. A. (2020). J. Appl. Cryst. 53, 226-235.
13. Farrugia, L. J. (2012). J. Appl. Cryst. 45, 849–854.

^{1}H NMR and ^{13}C{^{1}H} NMR spectra:

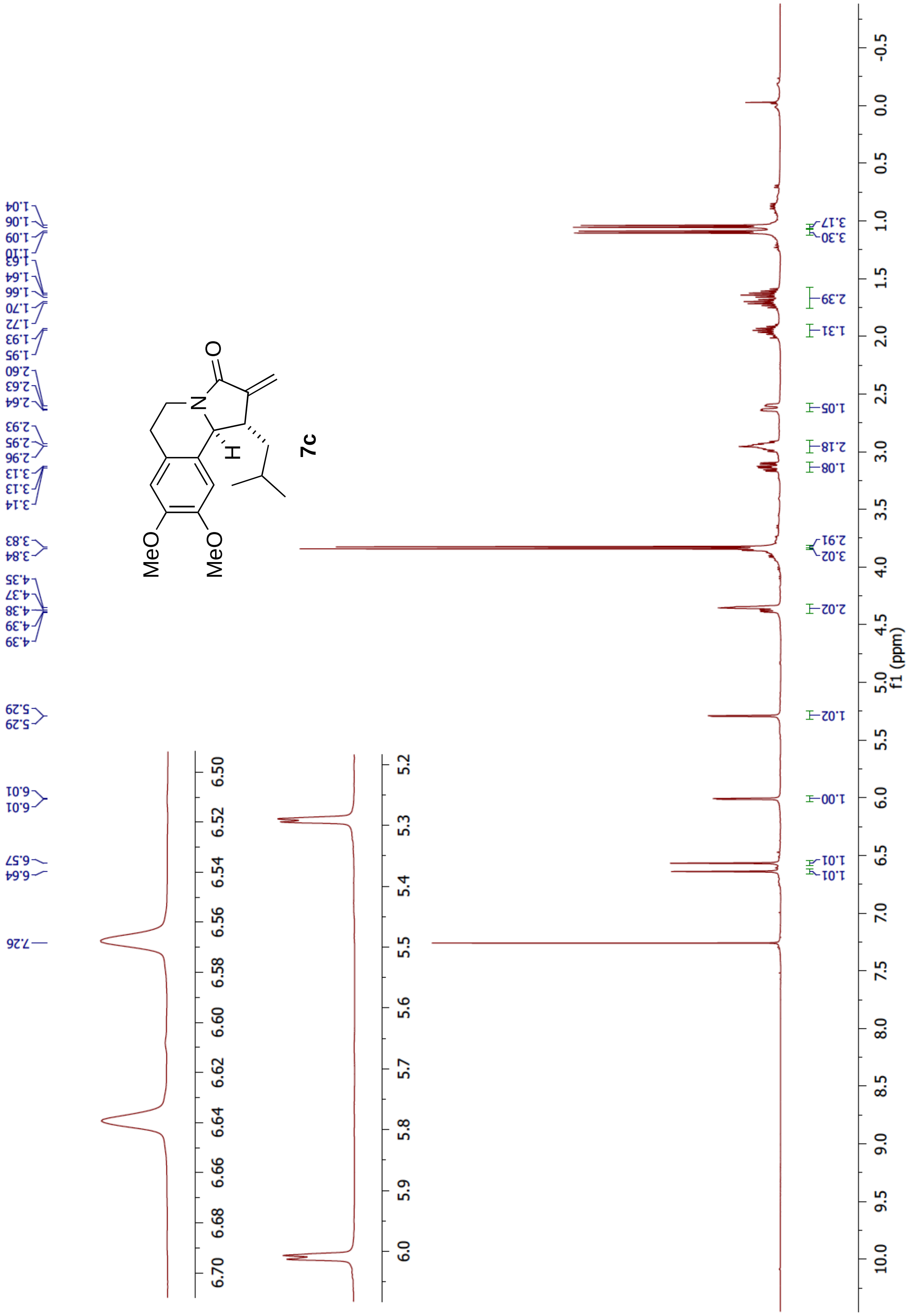

Figure S2: ^{1}H NMR spectrum of **7c** in $CDCl_3$.

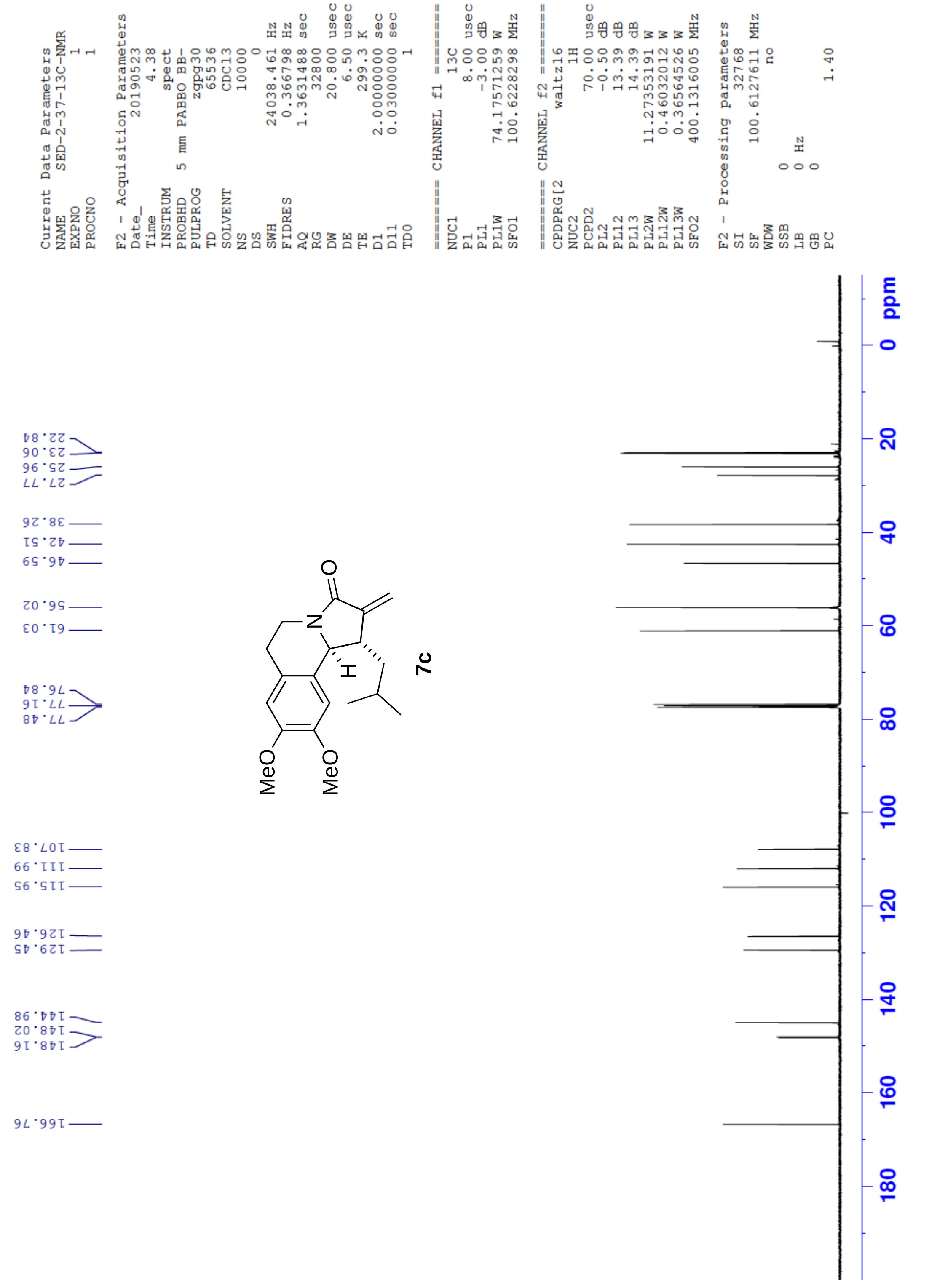

Figure S3: $^{13}C\{^{1}H\}$ NMR spectrum of **7c** in $CDCl_3$.

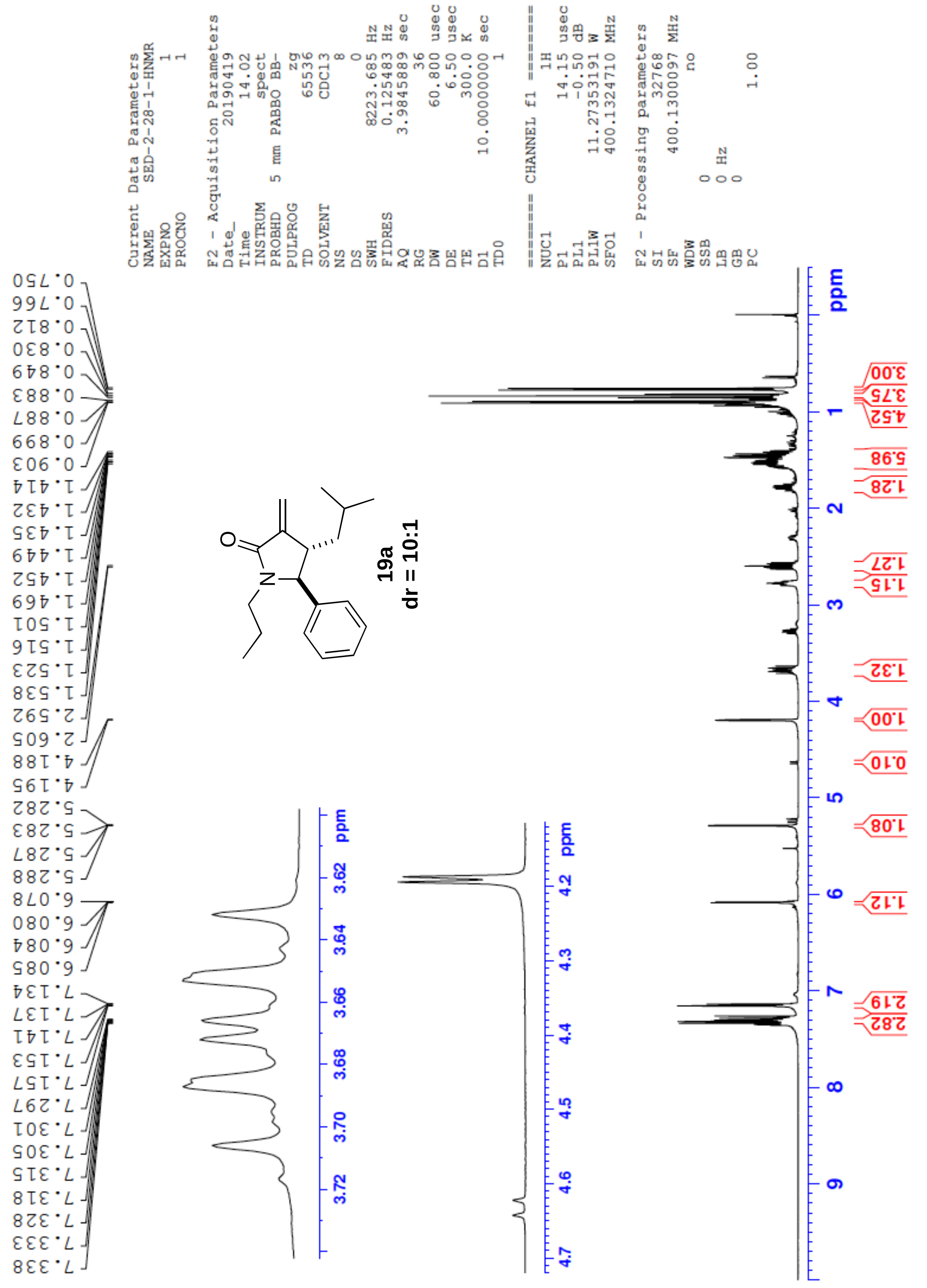

Figure S4: ^{1}H NMR spectrum of **19a** in $CDCl_3$.

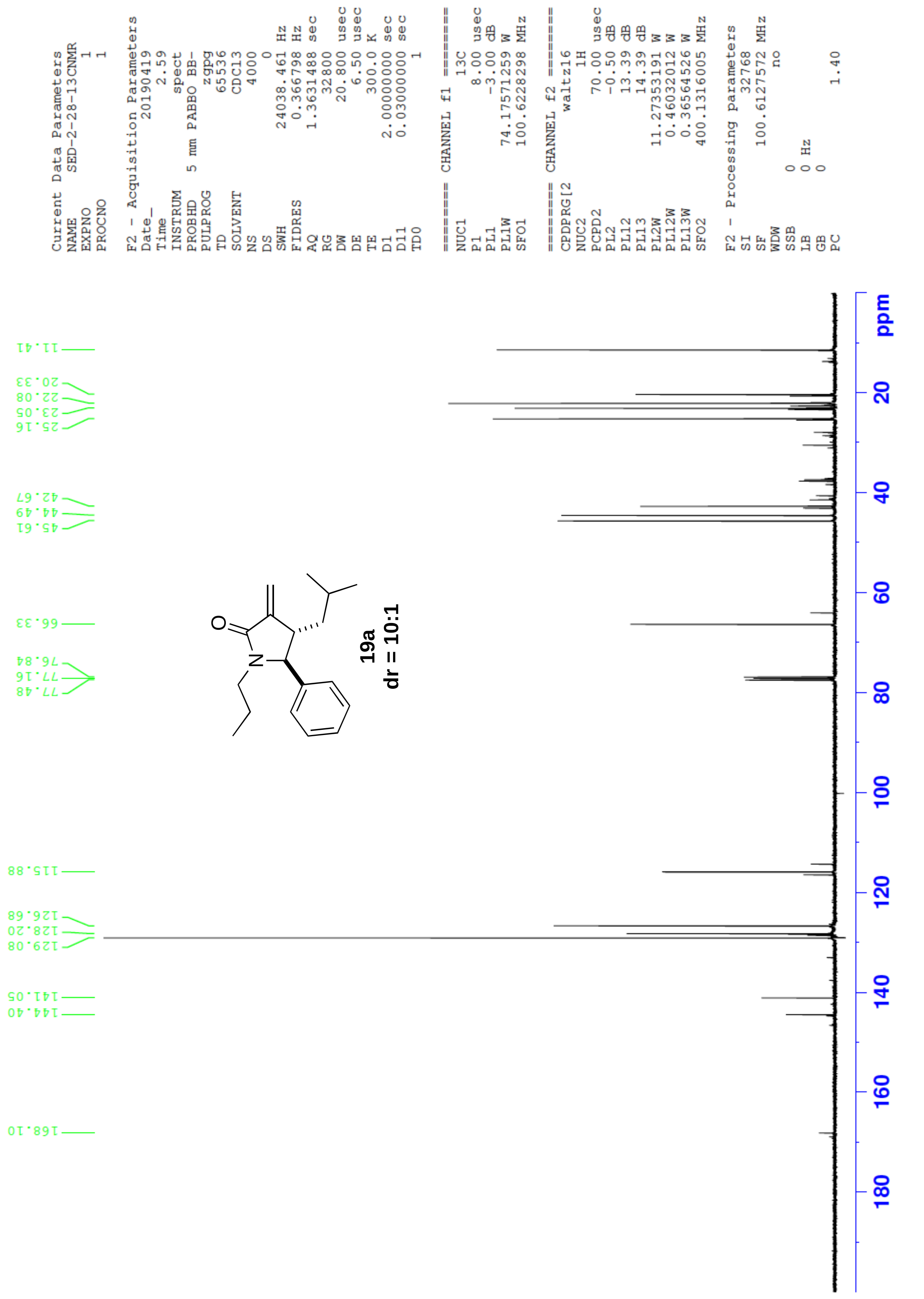

Figure S5: $^{13}C\{^{1}H\}$ NMR spectrum of **19a** in $CDCl_3$.

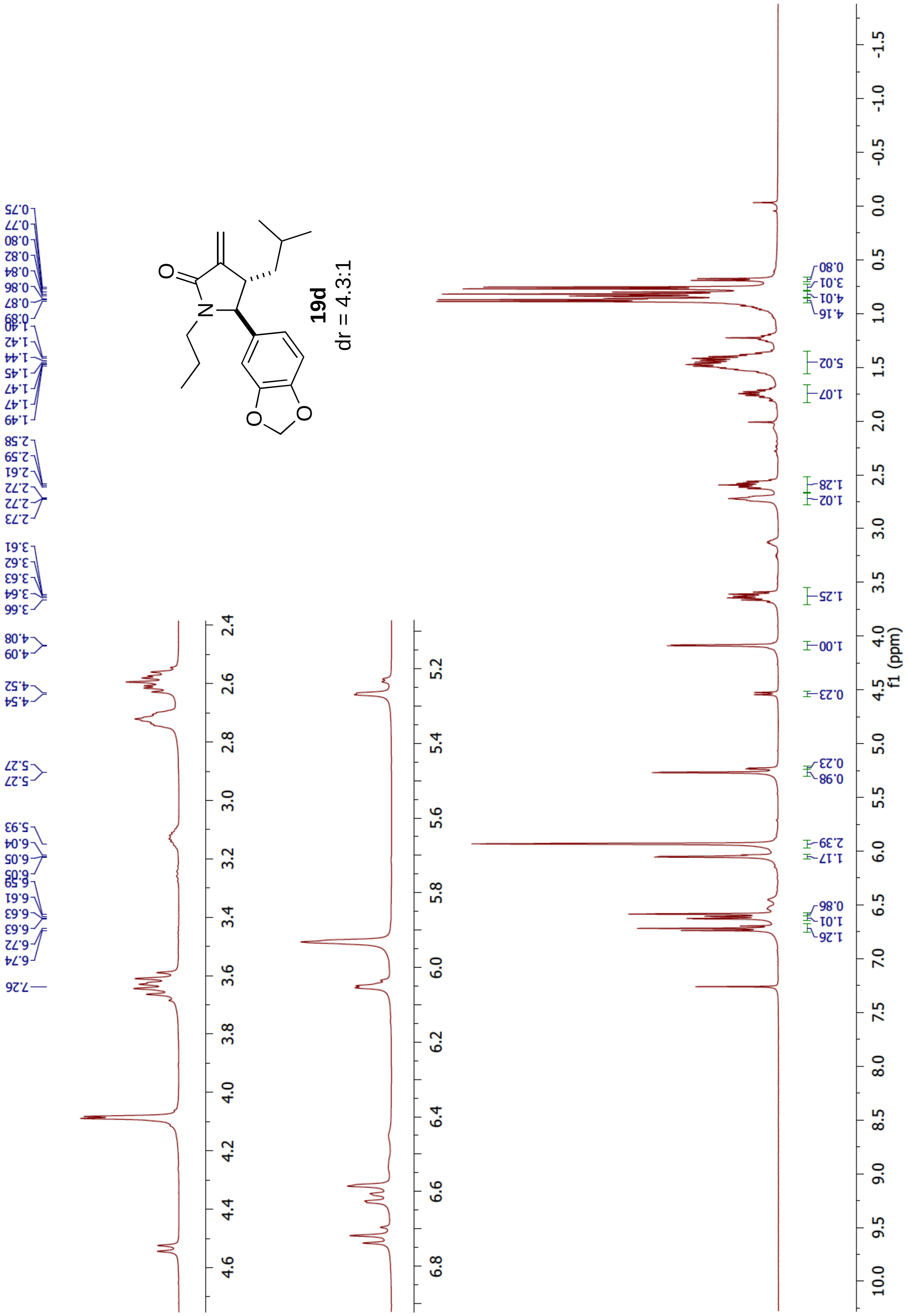

Figure S6: ^{1}H NMR spectrum of **19d** in $CDCl_3$.

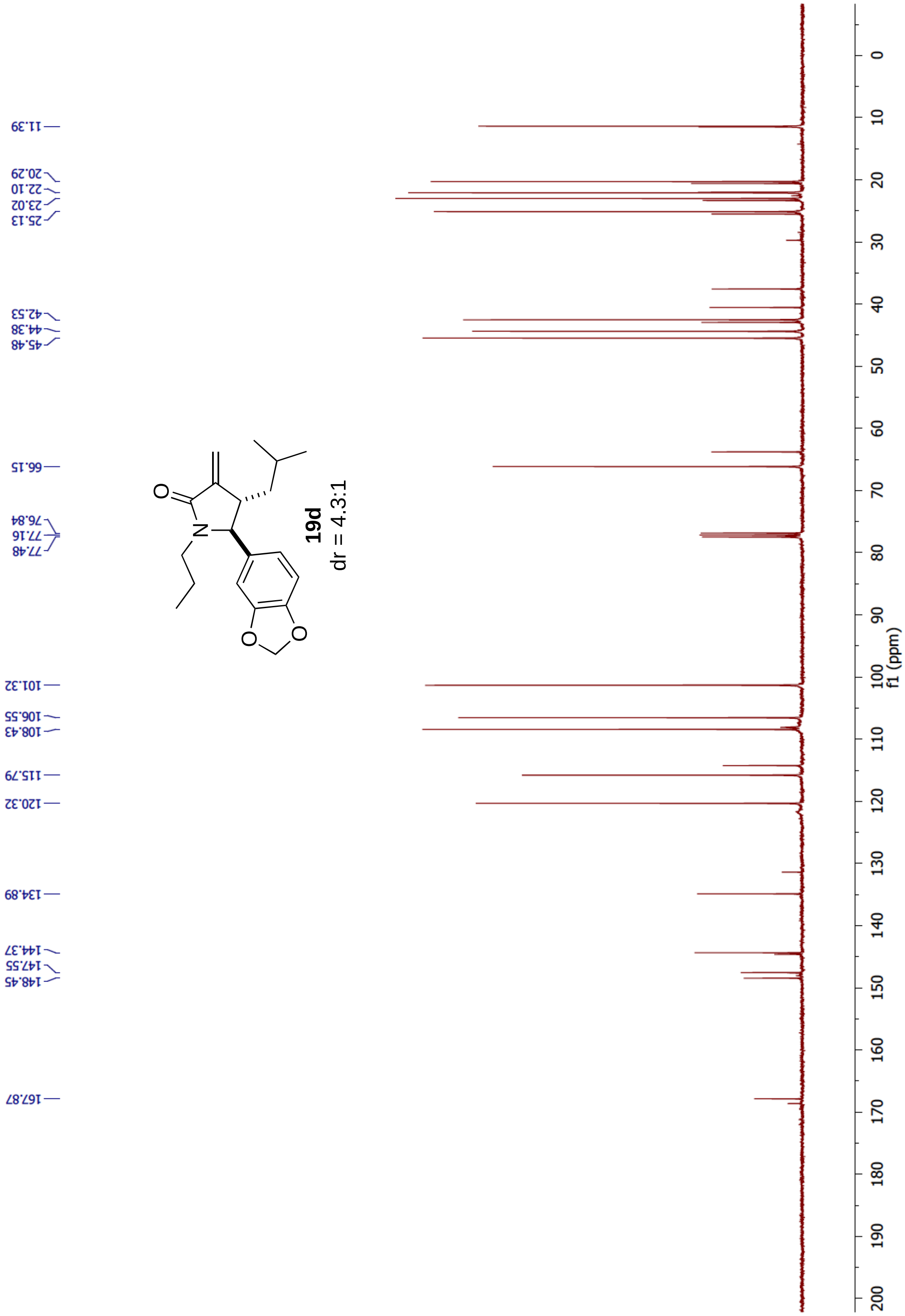

Figure S7: $^{13}C\{^{1}H\}$ NMR spectrum of **19d** in $CDCl_3$.

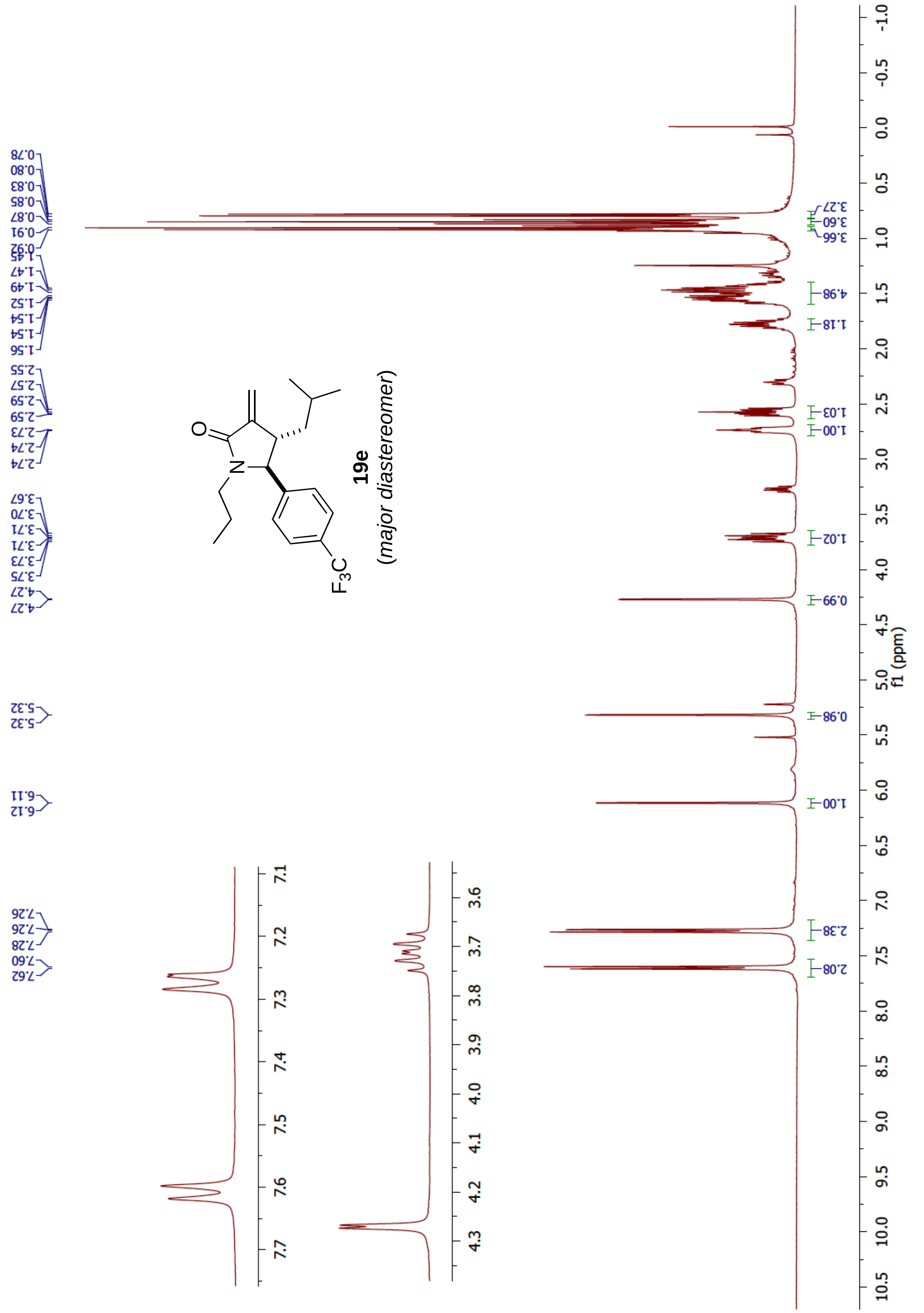

Figure S8: ^{1}H NMR spectrum of **19e** in $CDCl_3$.

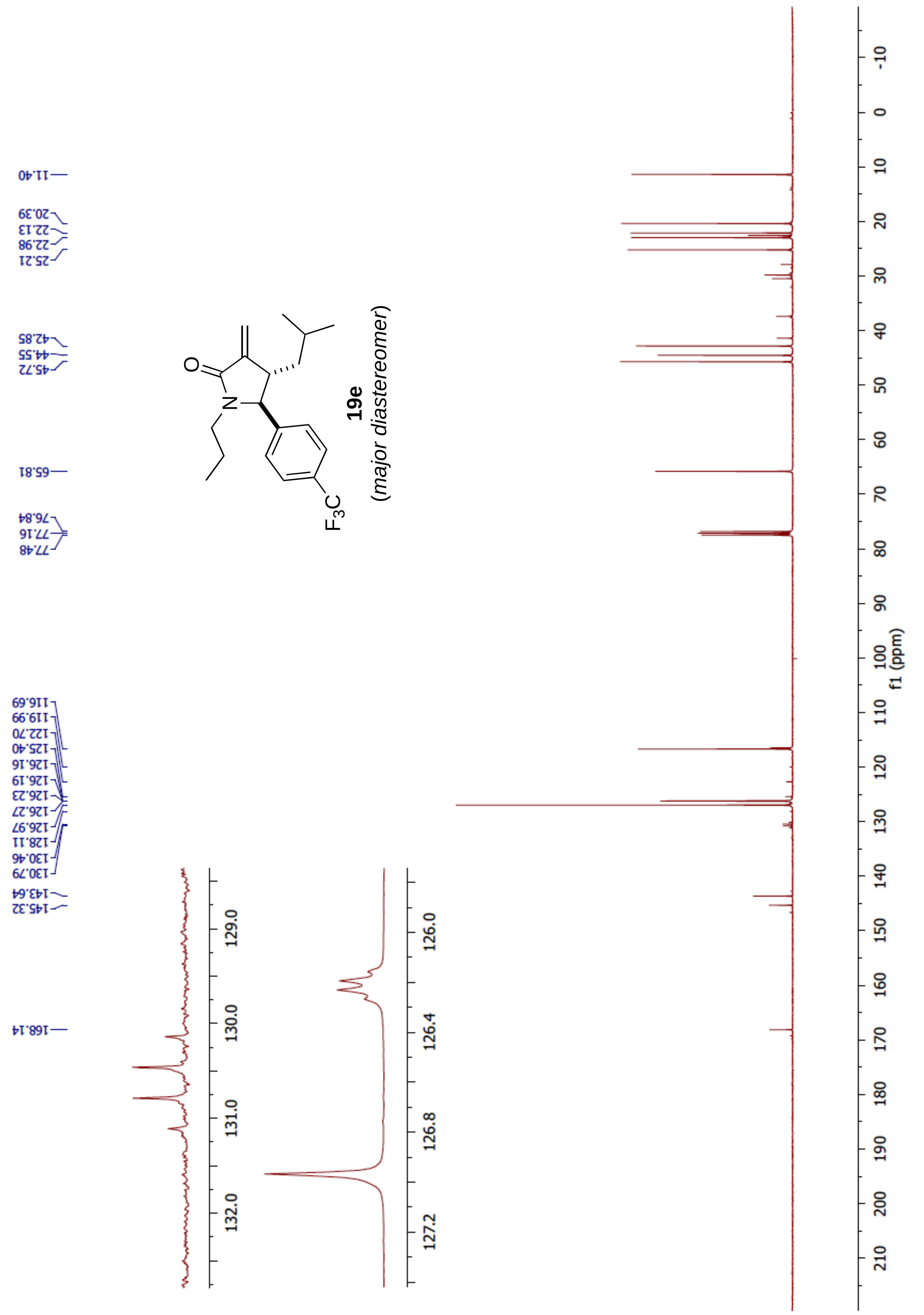

Figure S9: $^{13}C\{^{1}H\}$ NMR spectrum of **19e** in $CDCl_3$.

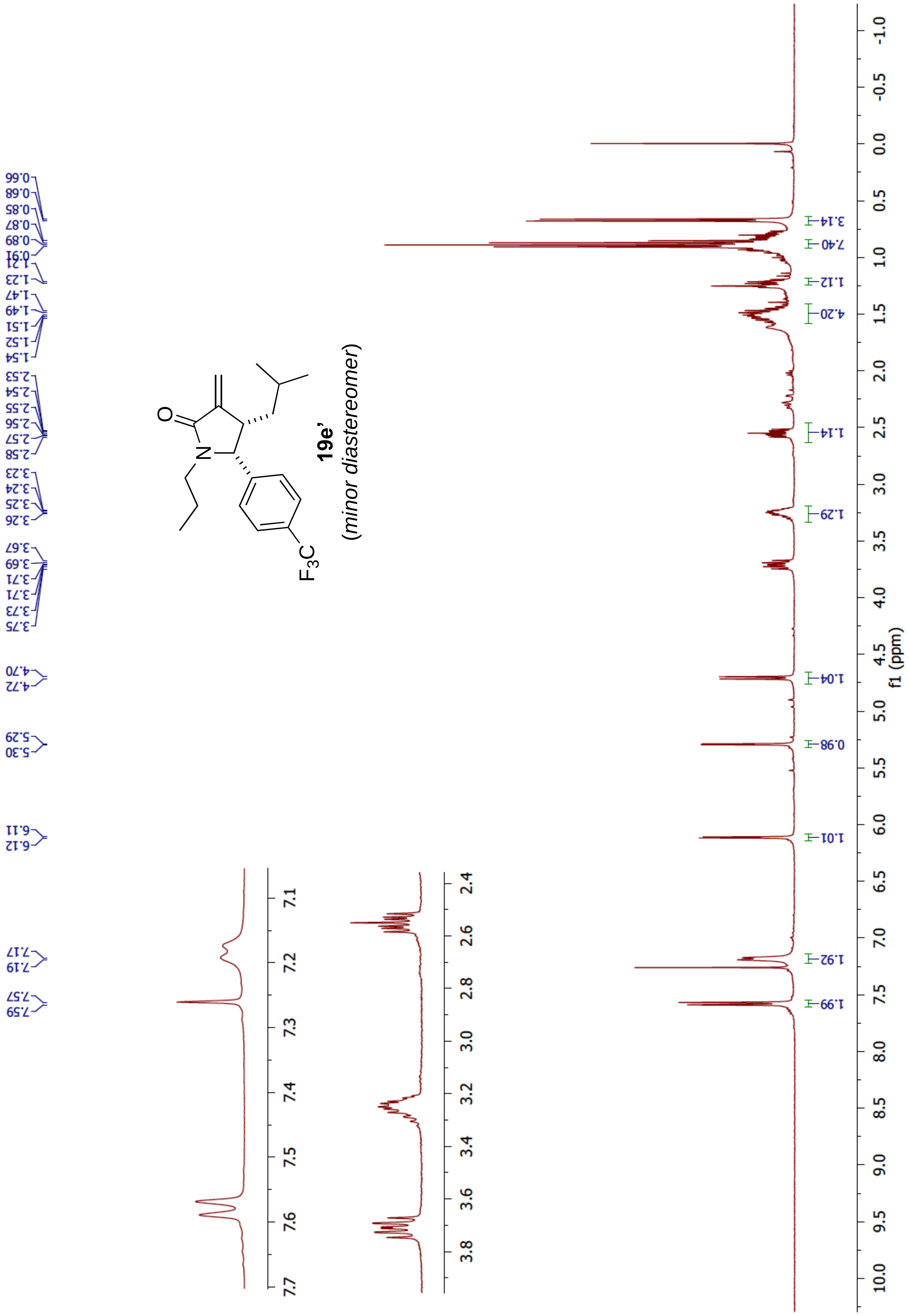

Figure S10: ^{1}H NMR spectrum of **19e'** in $CDCl_3$.

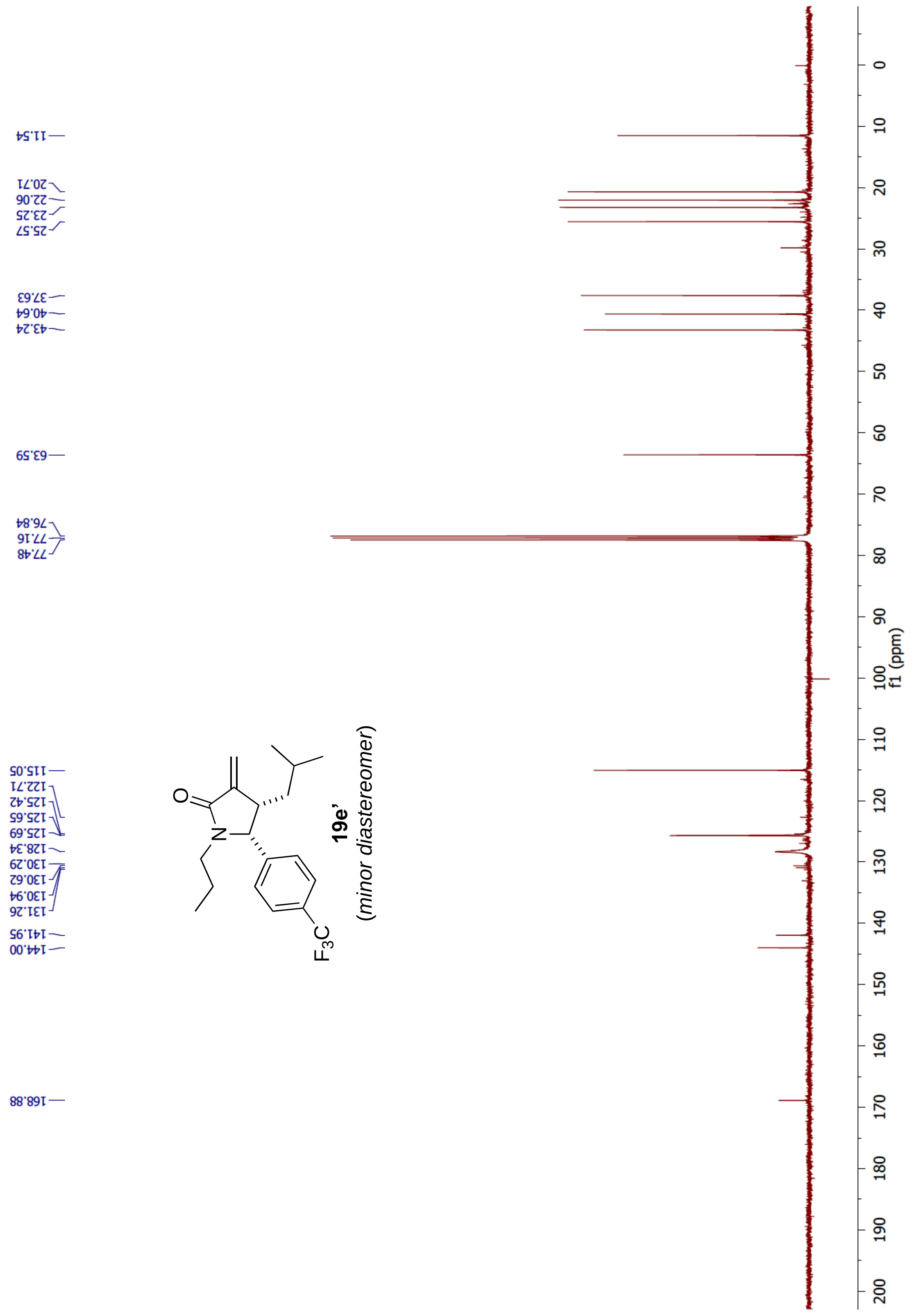

Figure S11: $^{13}C\{^{1}H\}$ NMR spectrum of **19e'** in $CDCl_3$.

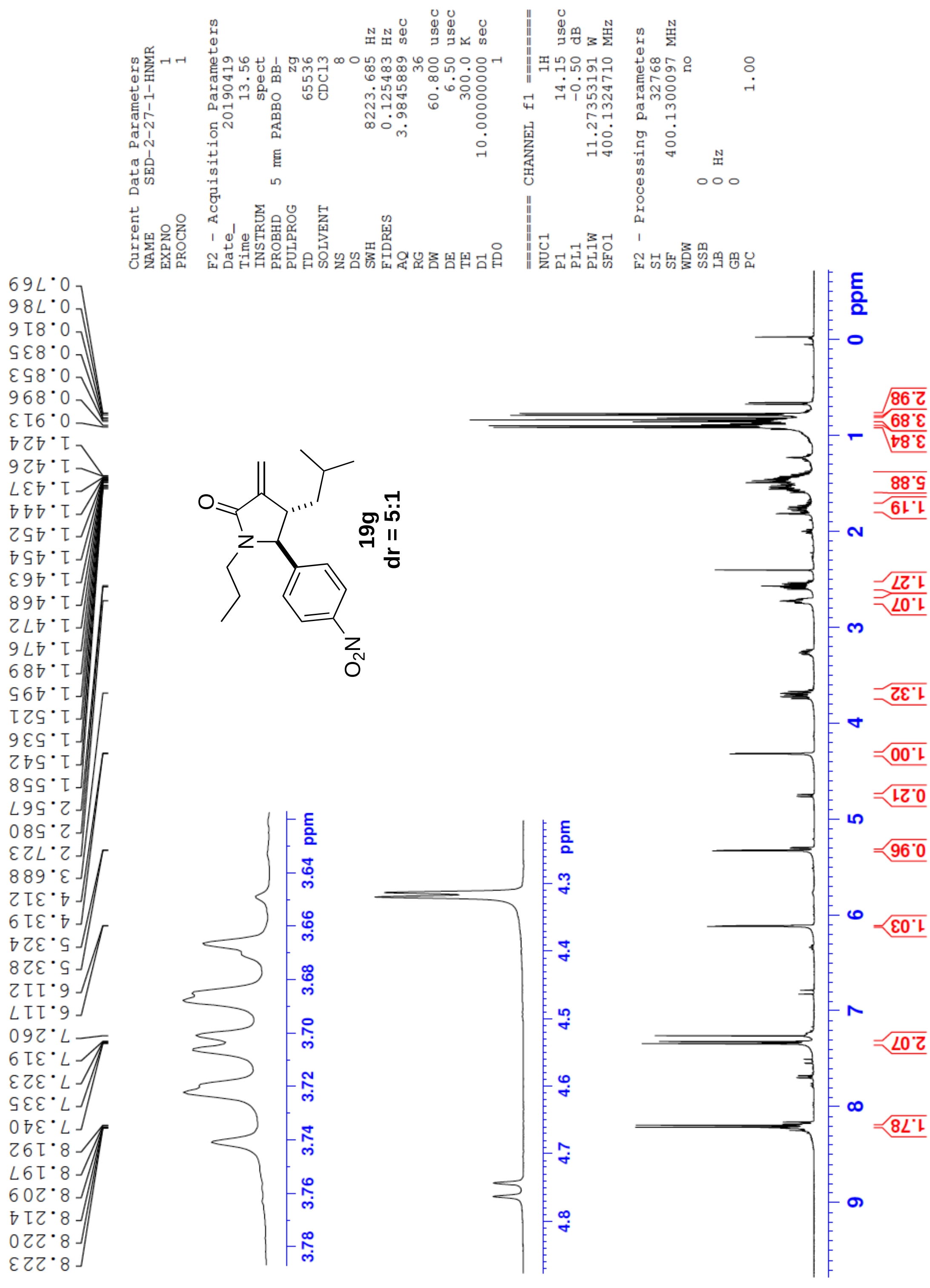

Figure S12: ^{1}H NMR spectrum of **19g** in $CDCl_3$.

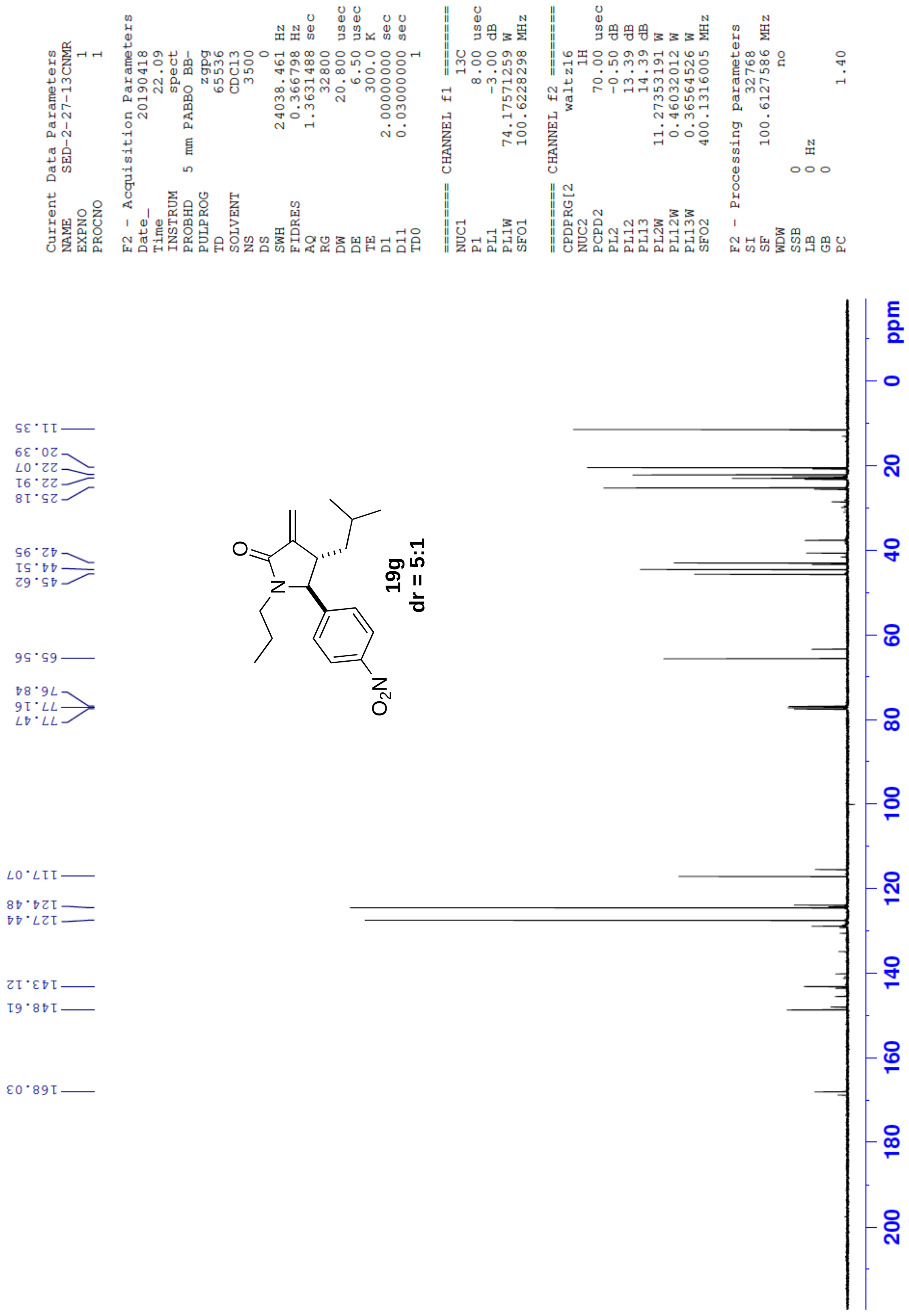

Figure S13: $^{13}C\{^{1}H\}$ NMR spectrum of **19g** in $CDCl_3$.

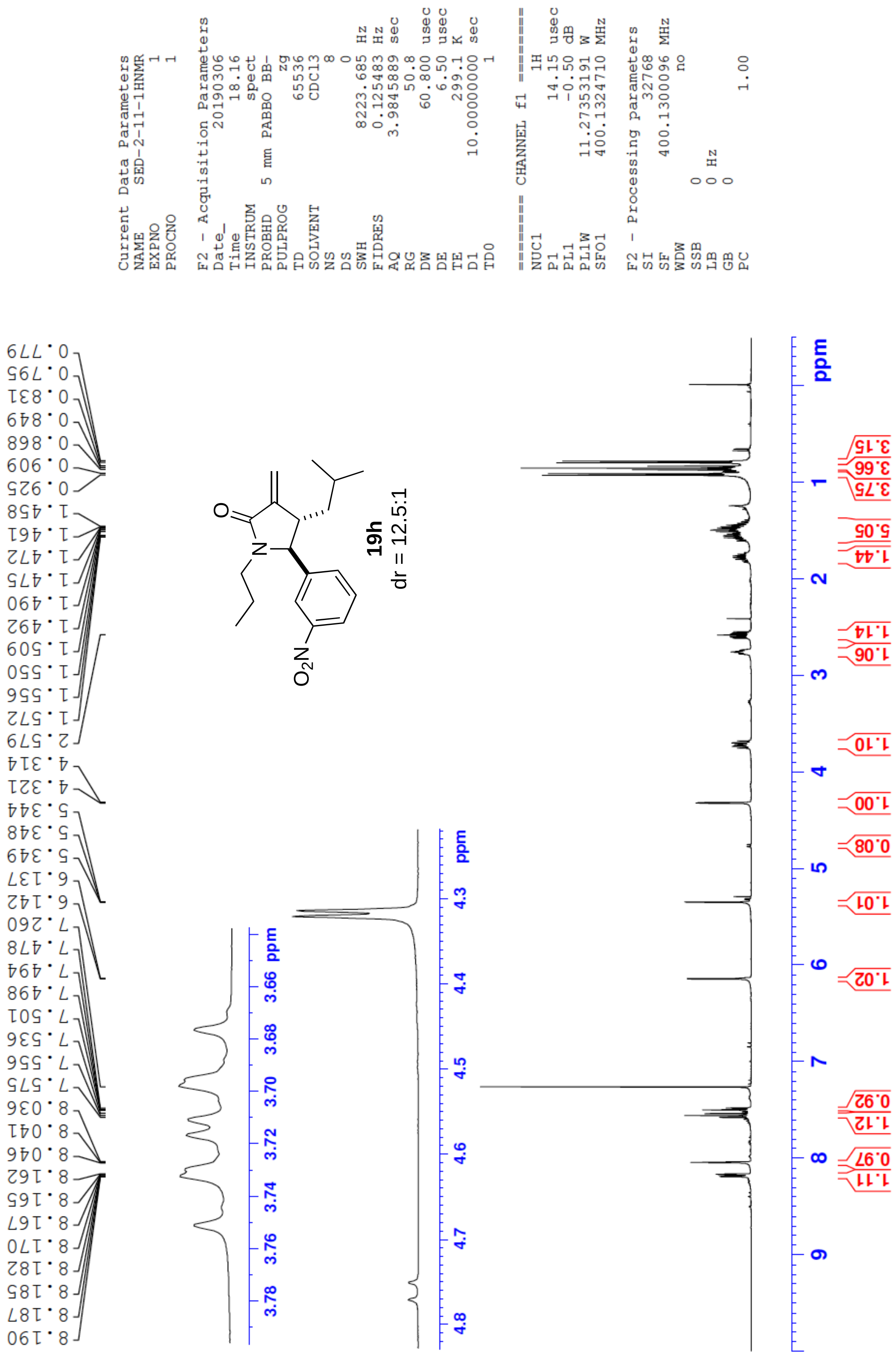

Figure S14: ^{1}H NMR spectrum of **19h** in $CDCl_3$.

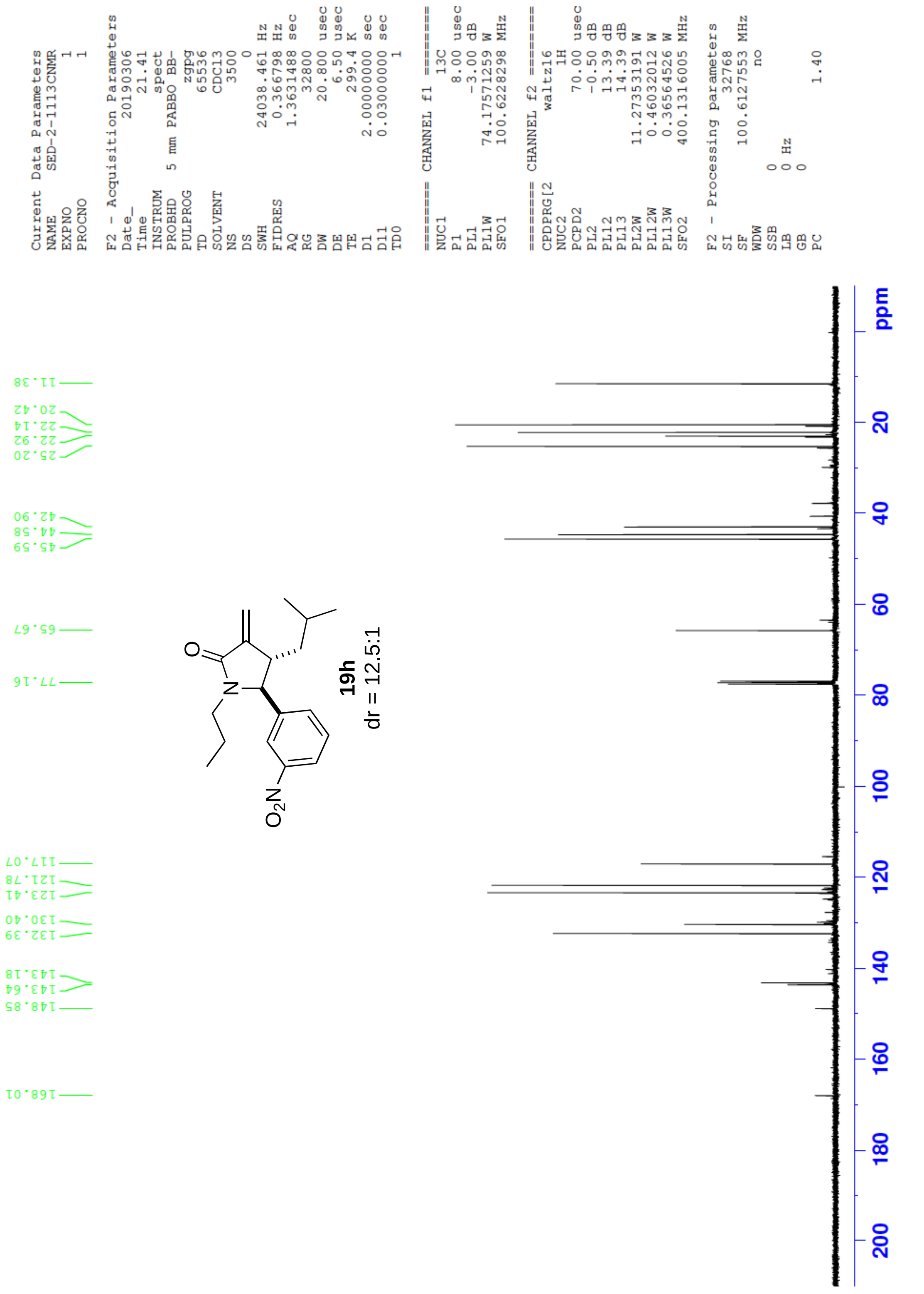

Figure S15: $^{13}C\{^{1}H\}$ NMR spectrum of **19h** in $CDCl_3$.

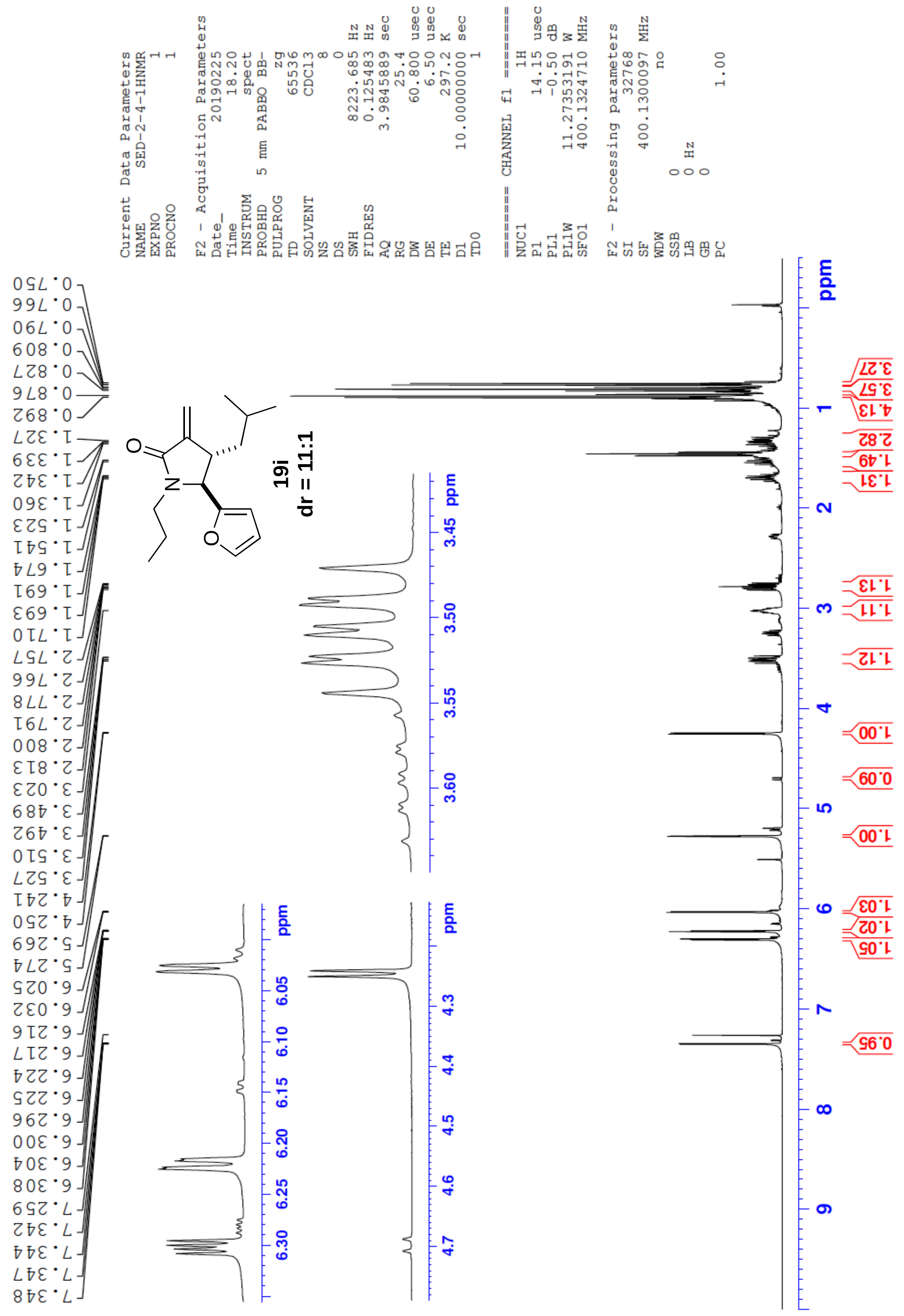

Figure S16: ^{1}H NMR spectrum of **19i** in $CDCl_3$.

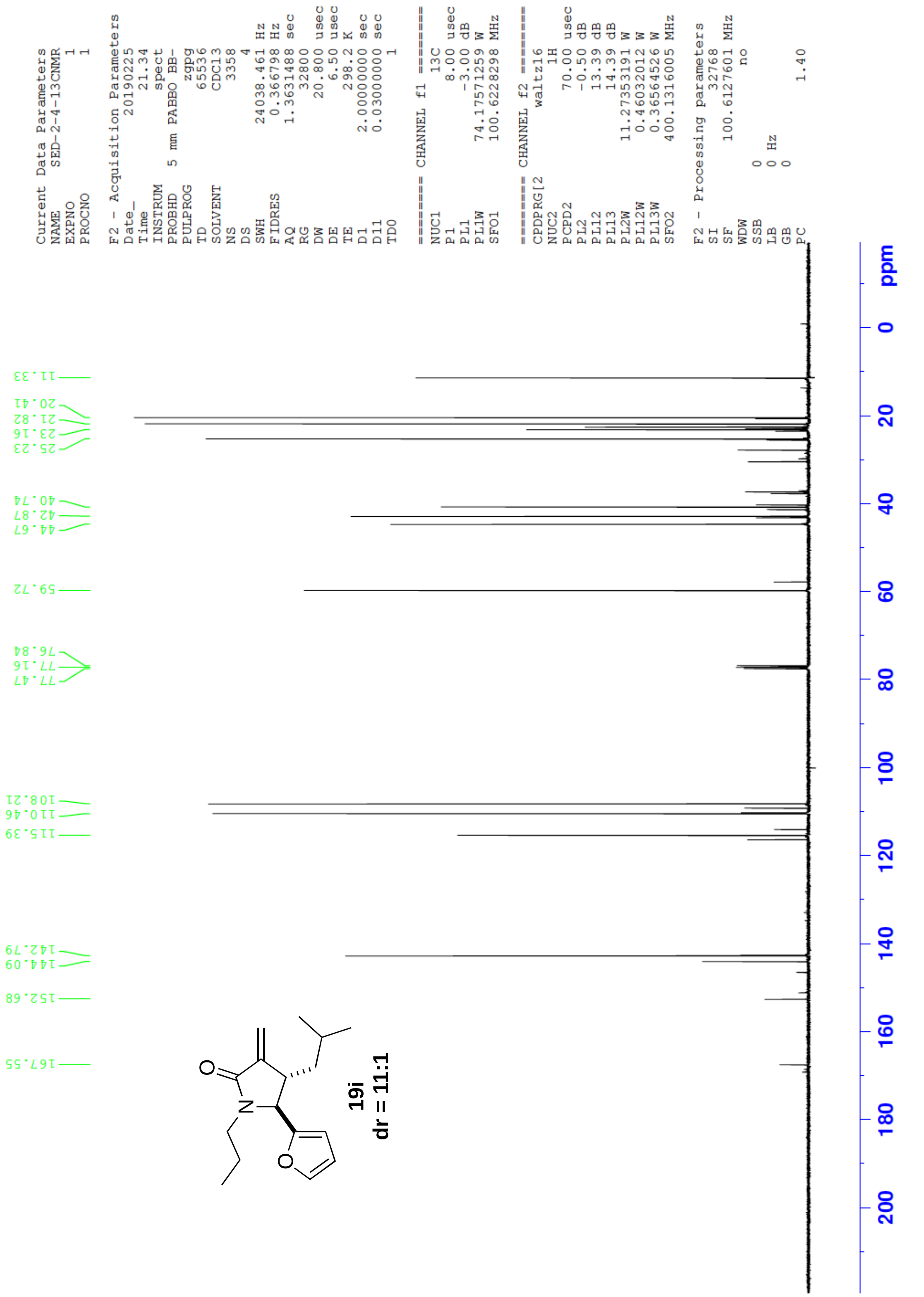

Figure S17: $^{13}C\{^{1}H\}$ NMR spectrum of **19i** in $CDCl_3$.

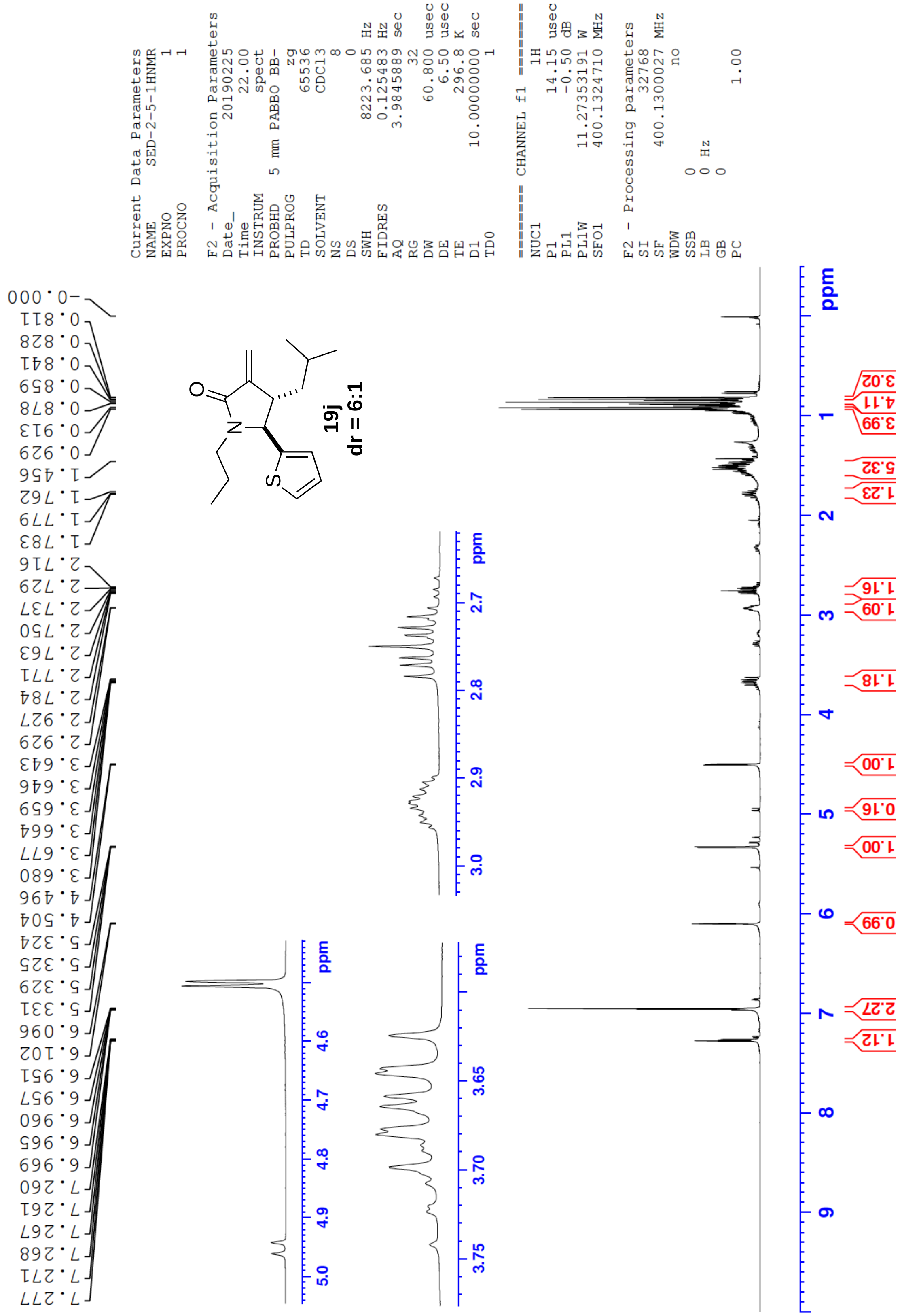

Figure S18: ^{1}H NMR spectrum of **19j** in $CDCl_3$.

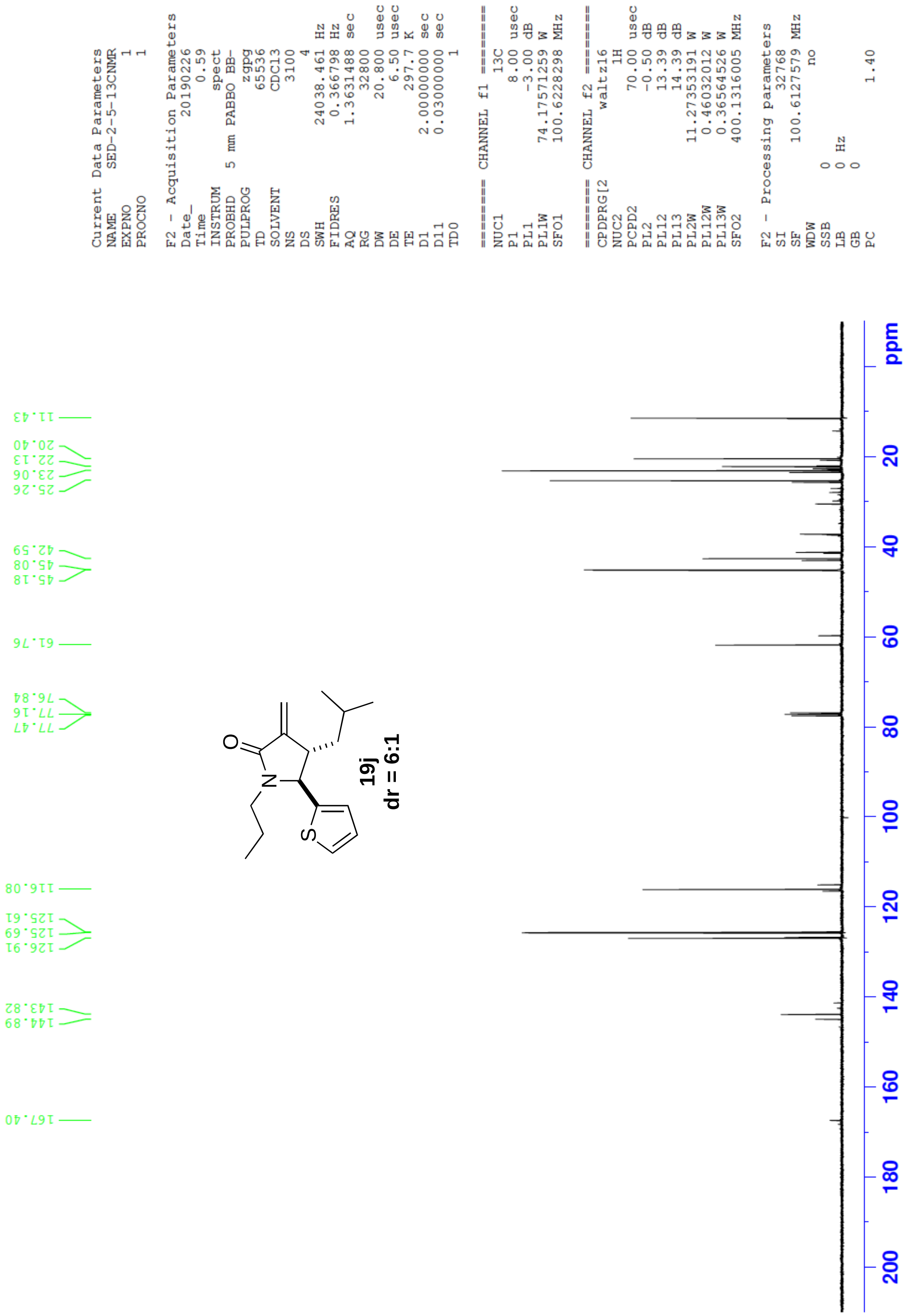

Figure S19: $^{13}C\{^{1}H\}$ NMR spectrum of **19j** in $CDCl_3$.

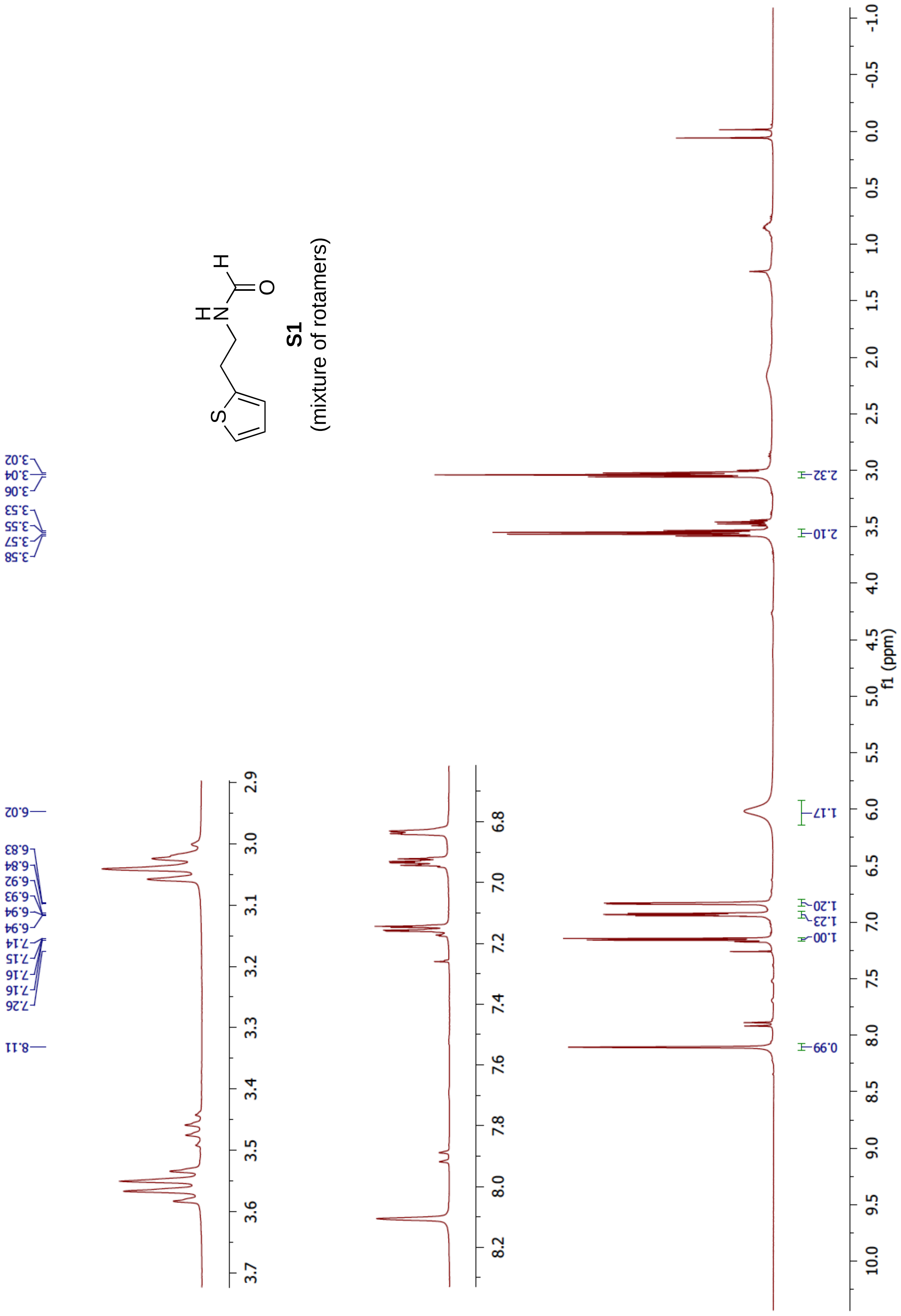

Figure S20: ^{1}H NMR spectrum of **S1** in $CDCl_3$.

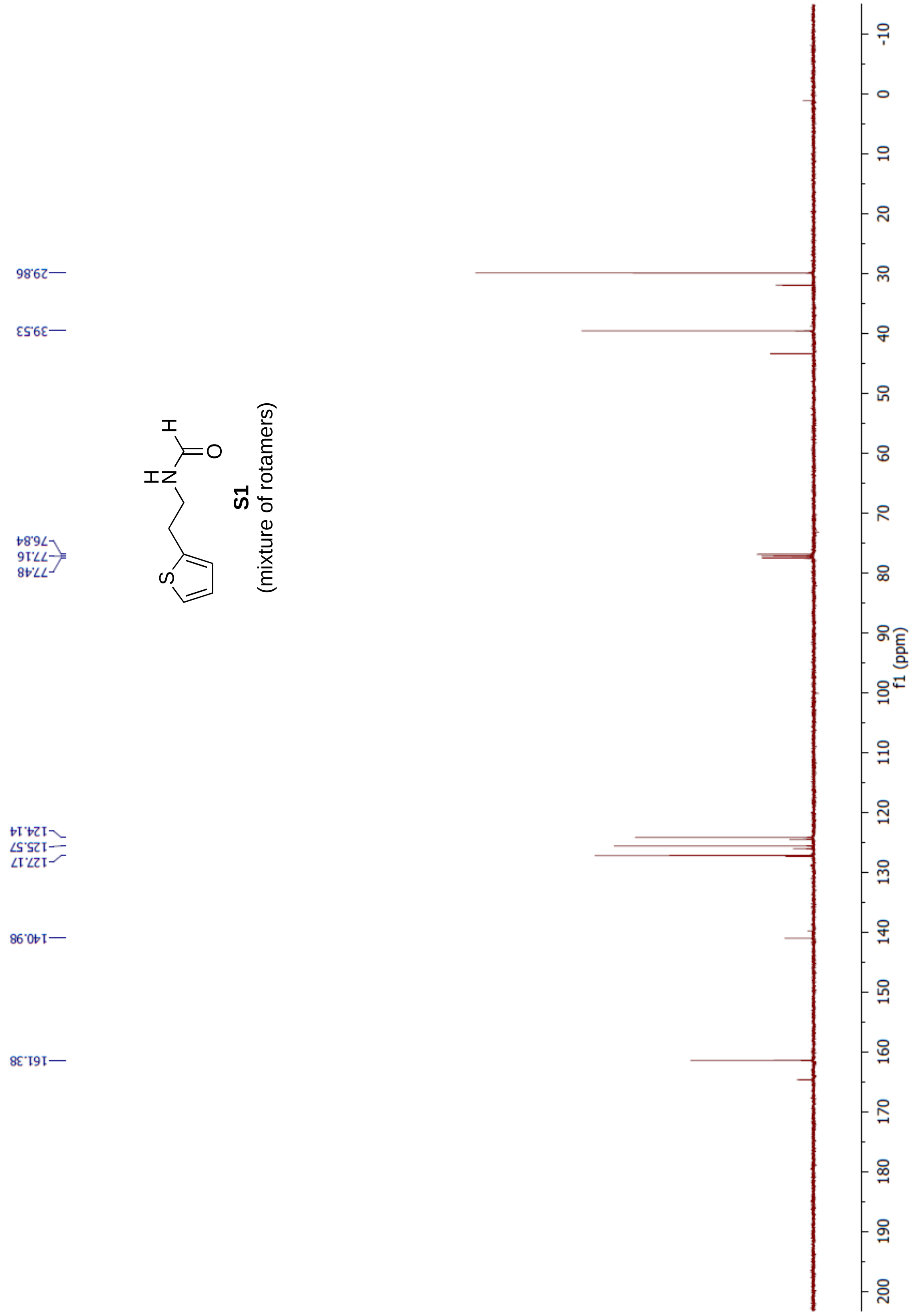

Figure S21: $^{13}C\{^{1}H\}$ NMR spectrum of **S1** in $CDCl_3$.

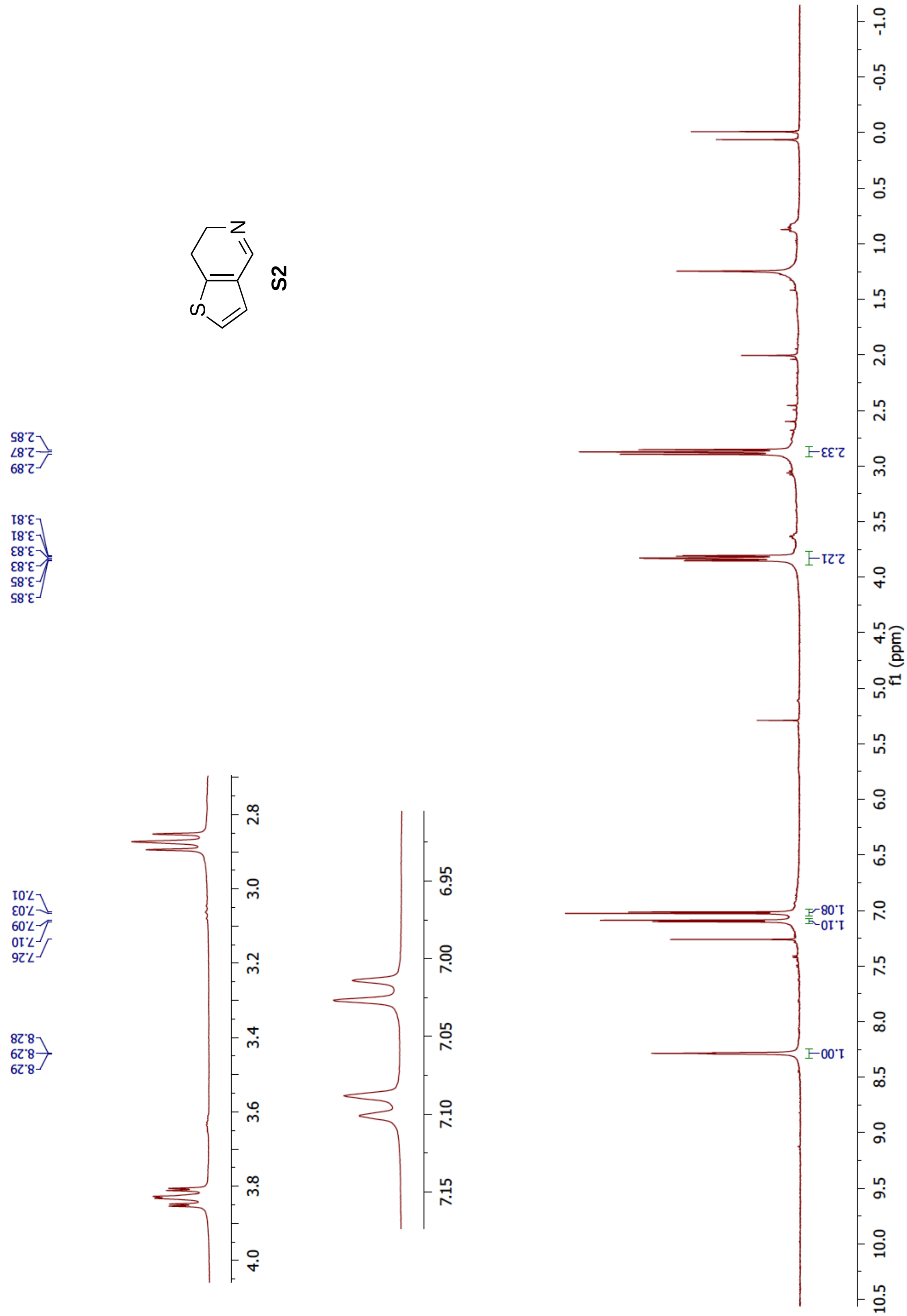

Figure S22: ^{1}H NMR spectrum of **S2** in $CDCl_3$.

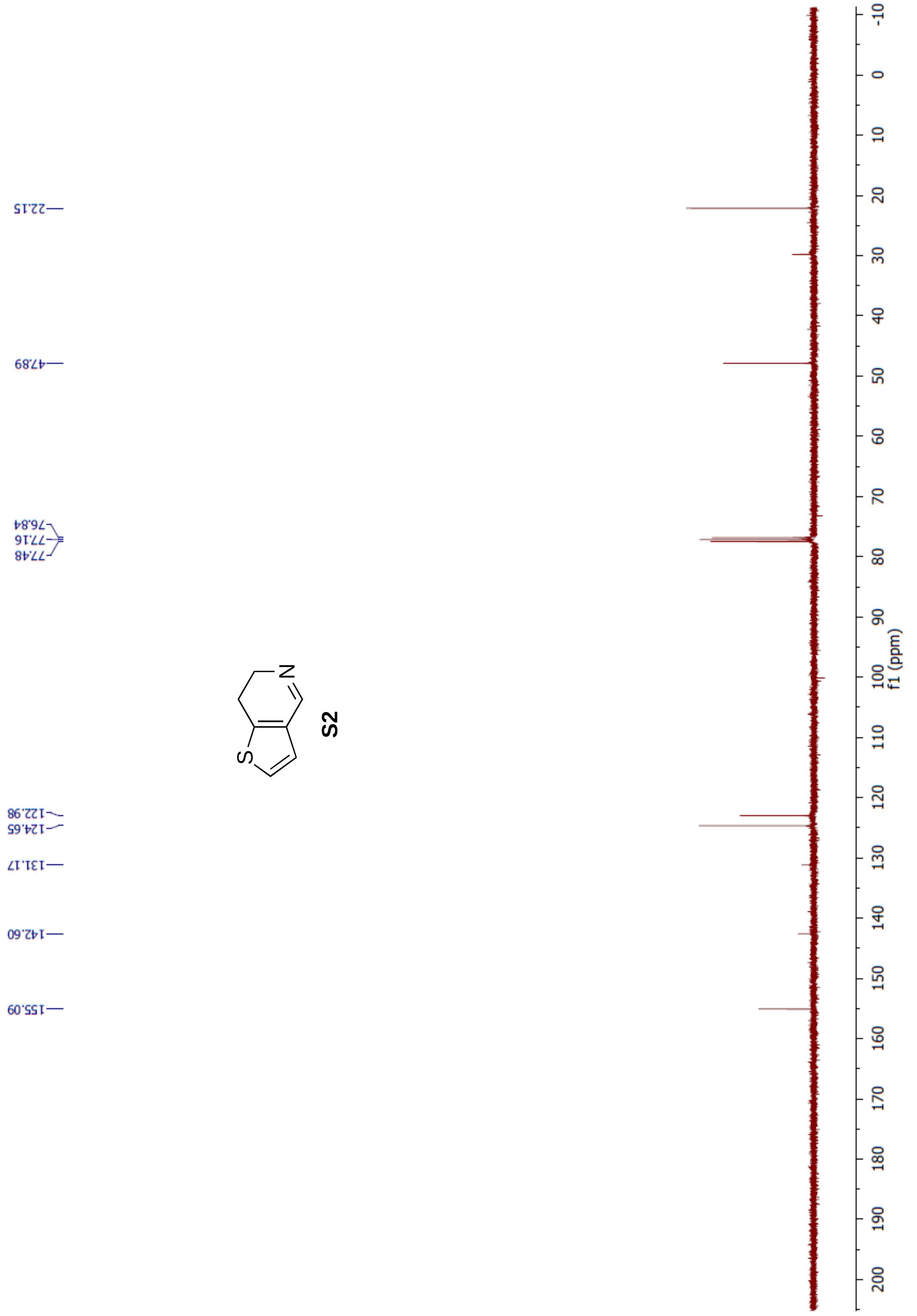

Figure S23: **^{13}C{^{1}H}** NMR spectrum of **S2** in $CDCl_3$.

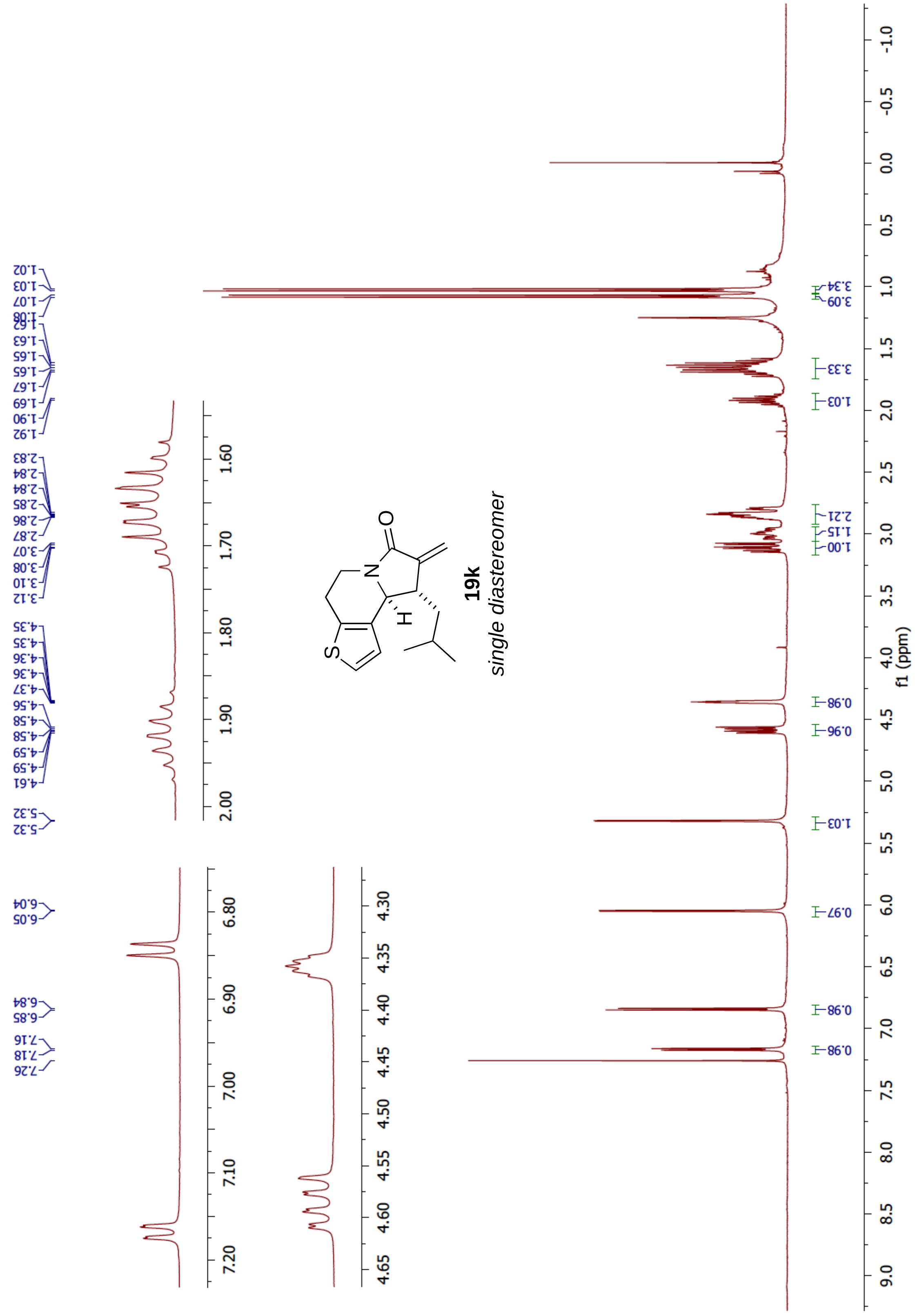

Figure S24: ^{1}H NMR spectrum of **19k** in $CDCl_3$.

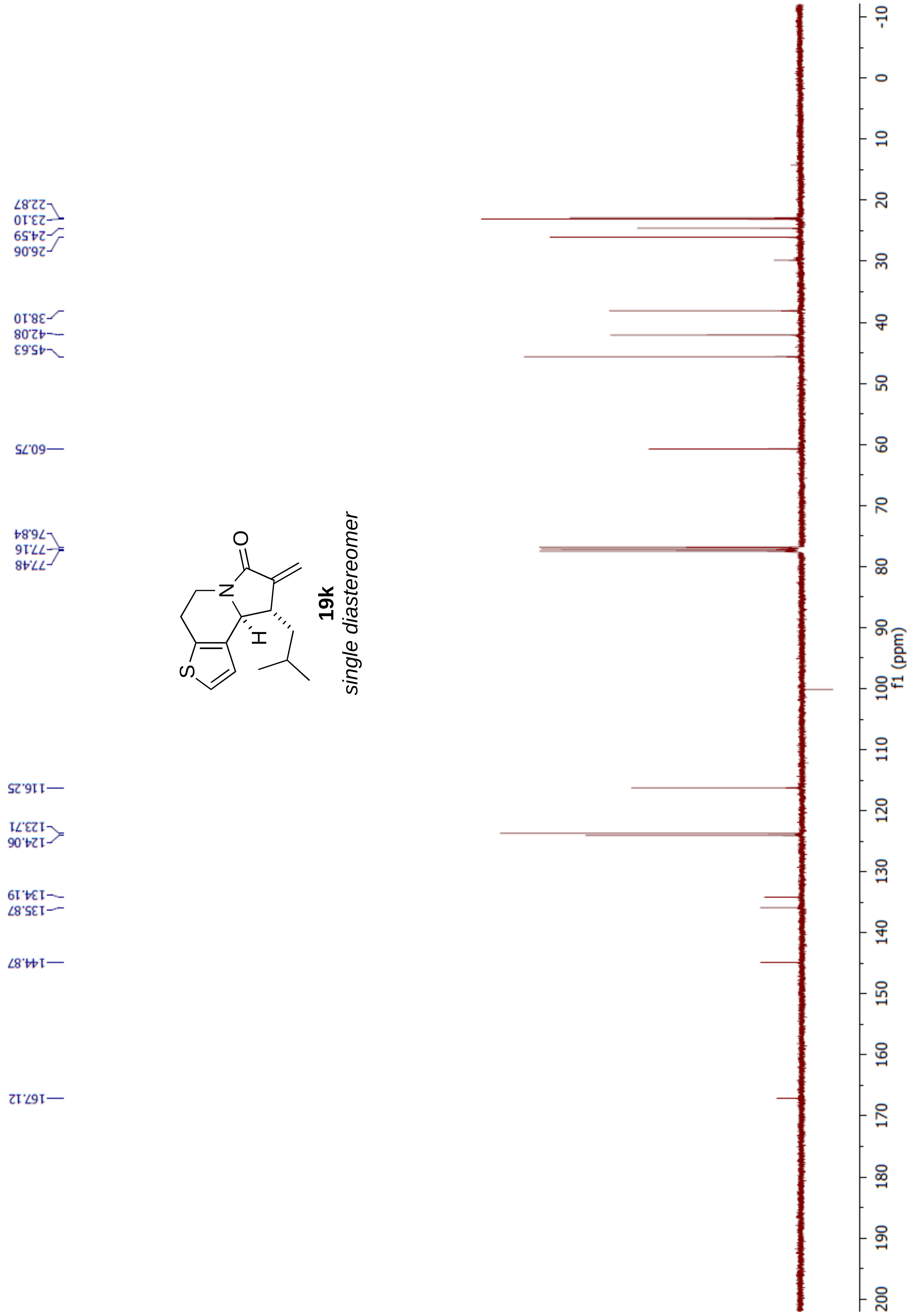

Figure S25: $^{13}C\{^{1}H\}$ NMR spectrum of **19k** in $CDCl_3$.

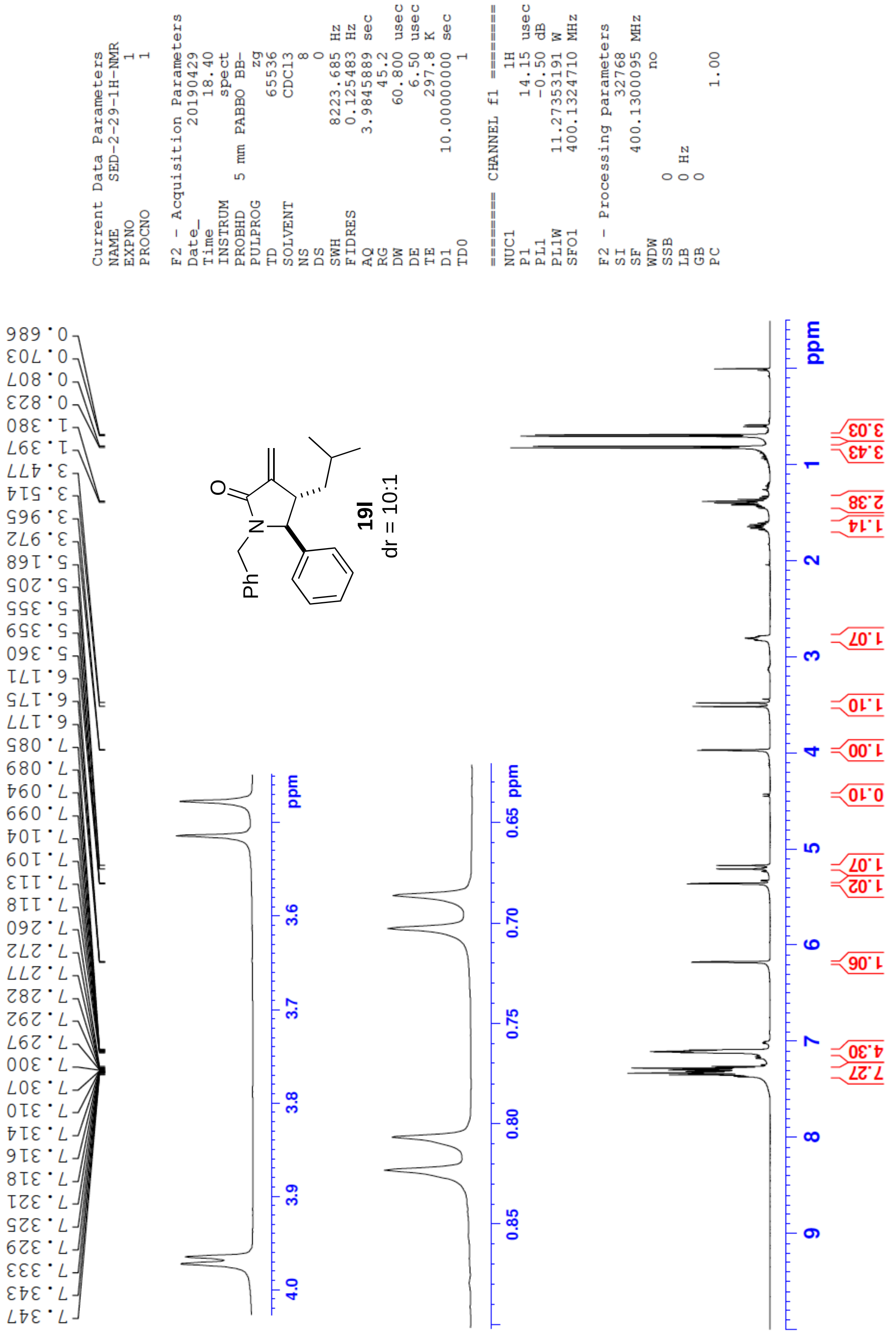

Figure S26: ^{1}H NMR spectrum of **19l** in $CDCl_3$.

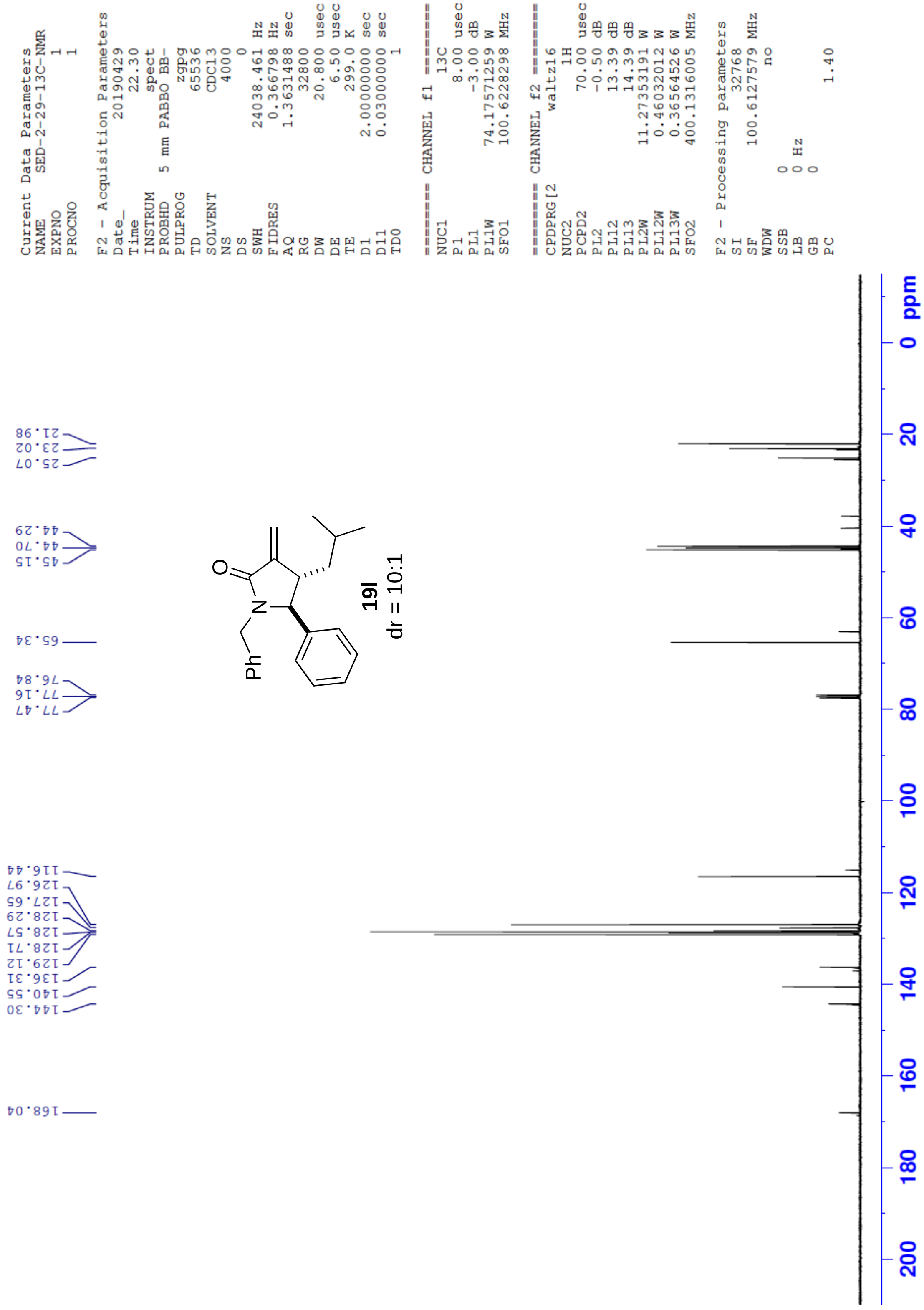

Figure S27: $^{13}C\{^{1}H\}$ NMR spectrum of **19l** in $CDCl_3$.

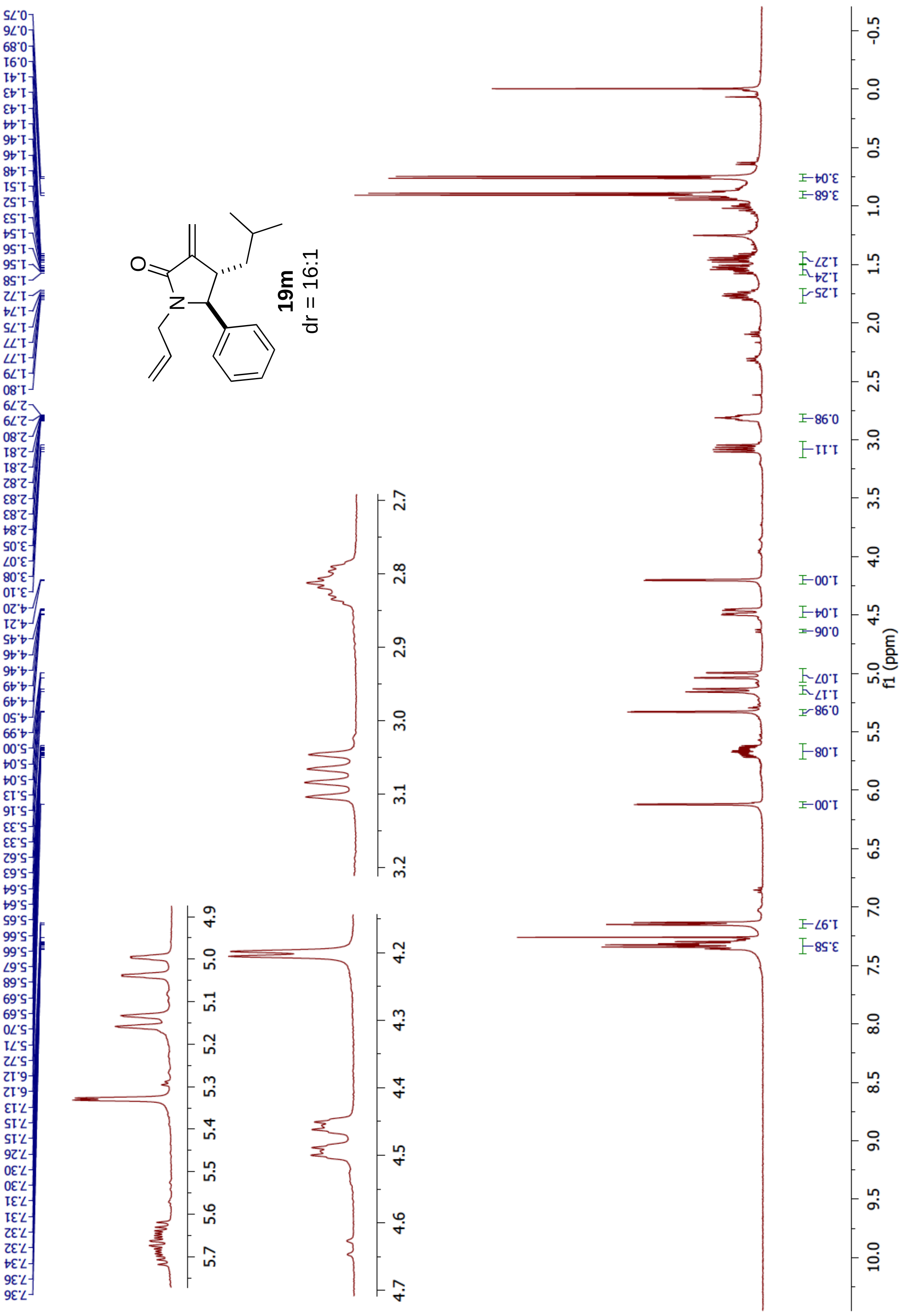

Figure S28: ^{1}H NMR spectrum of **19m** in $CDCl_3$.

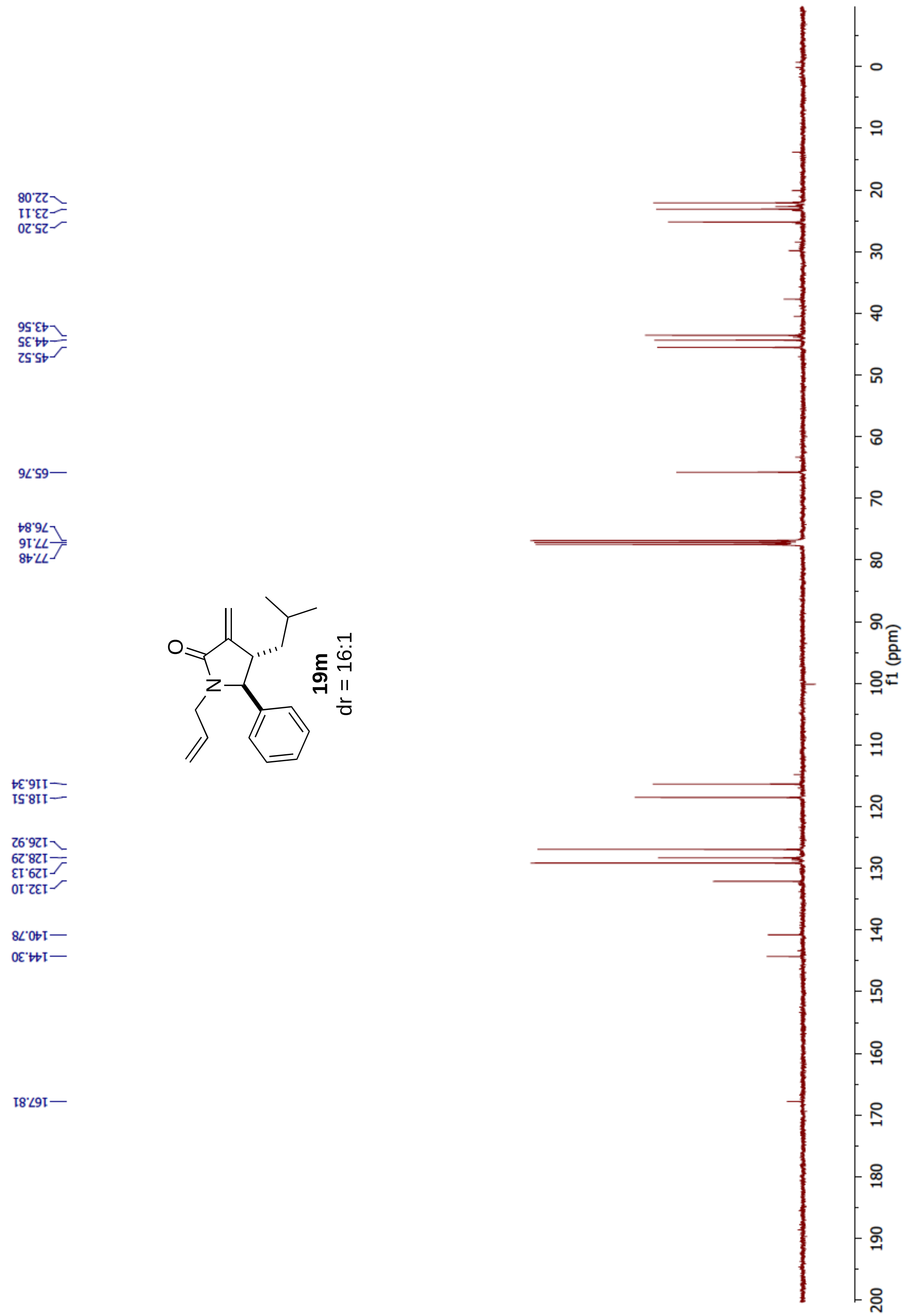

Figure S29: $^{13}C\{^{1}H\}$ NMR spectrum of **19m** in $CDCl_3$.

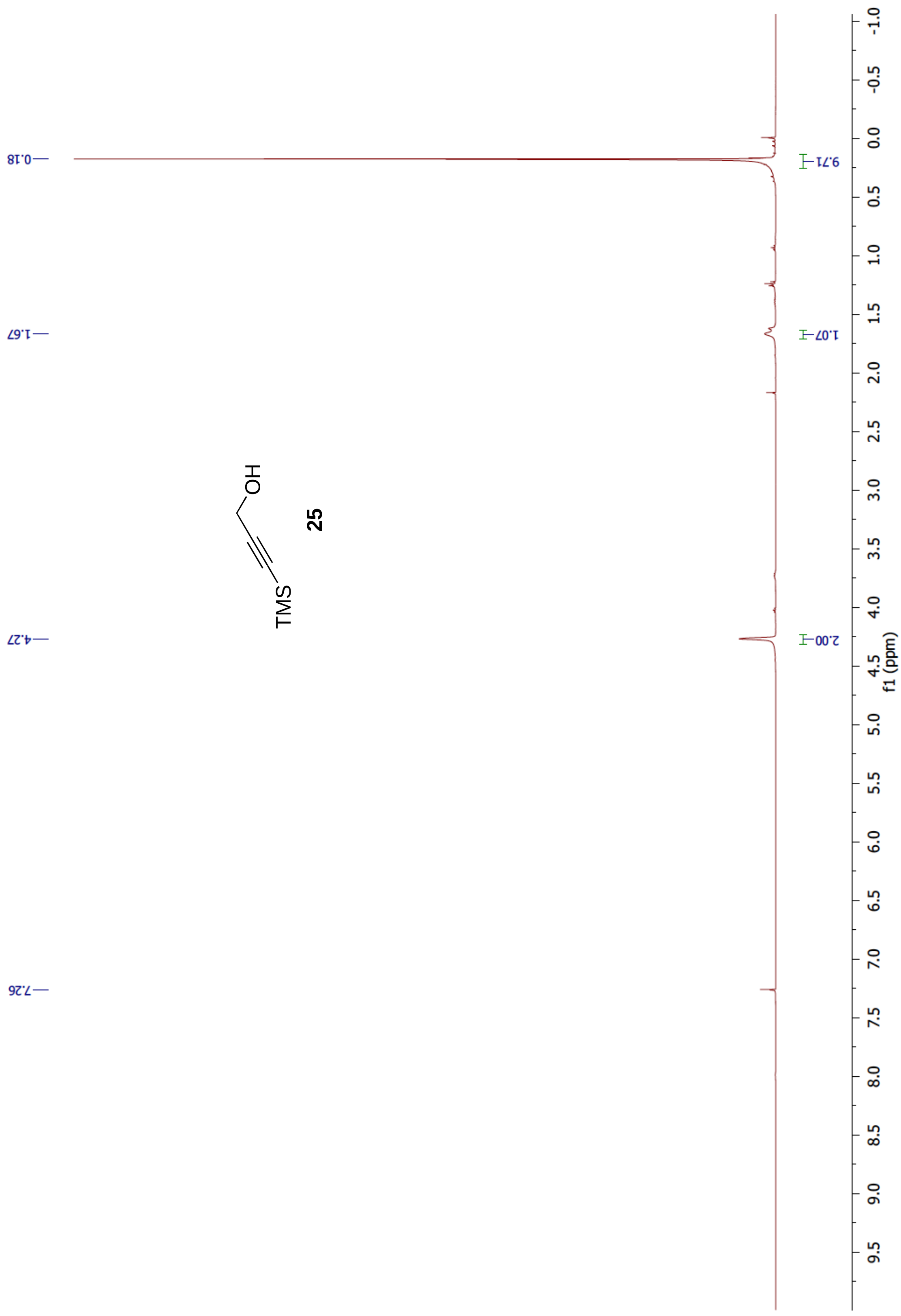

Figure S30: ^{1}H NMR spectrum of **25** in $CDCl_3$.

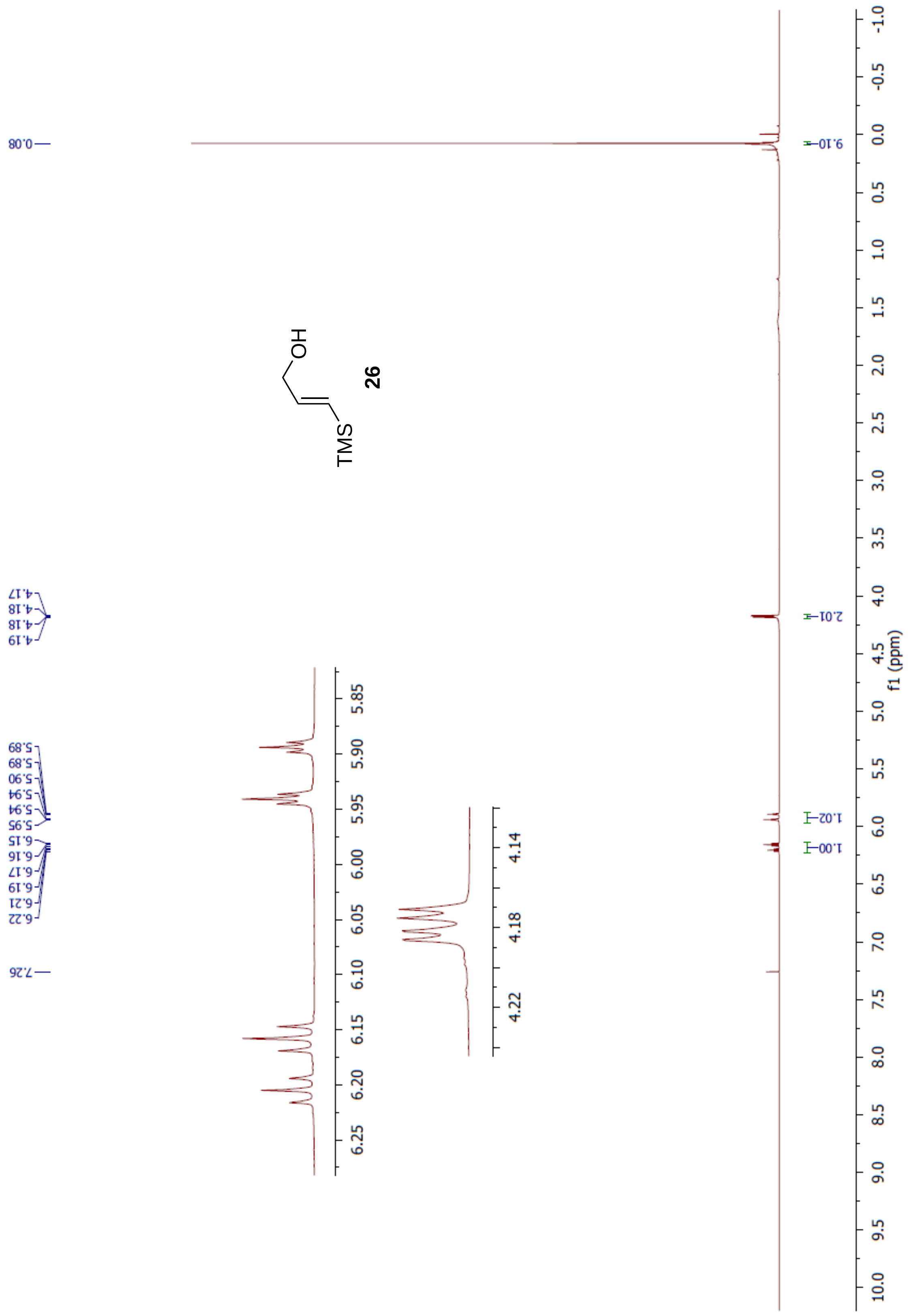

Figure S31: ^{1}H NMR spectrum of **26** in $CDCl_3$.

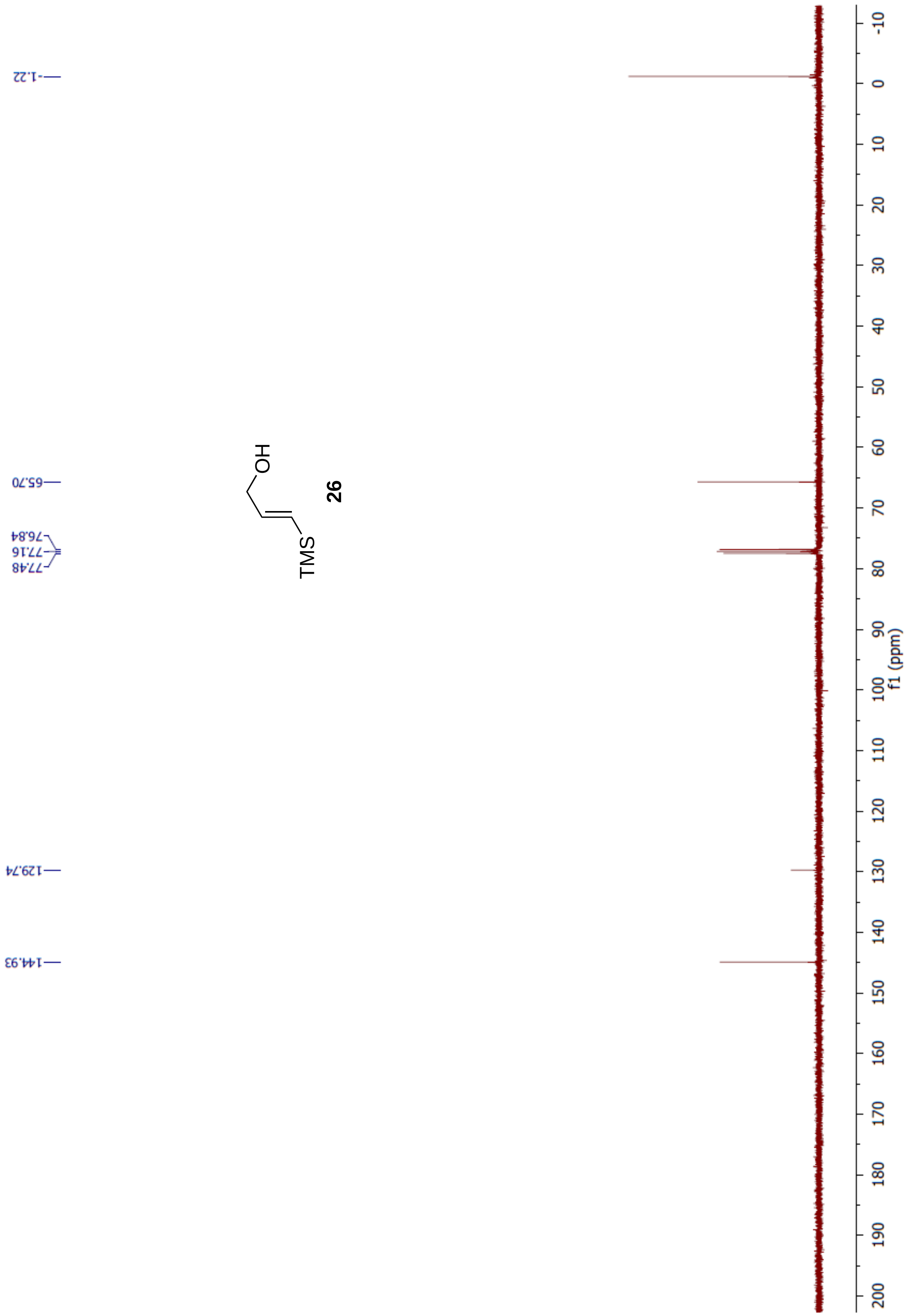

Figure S32: $^{13}C\{^{1}H\}$ NMR spectrum of **26** in $CDCl_3$.

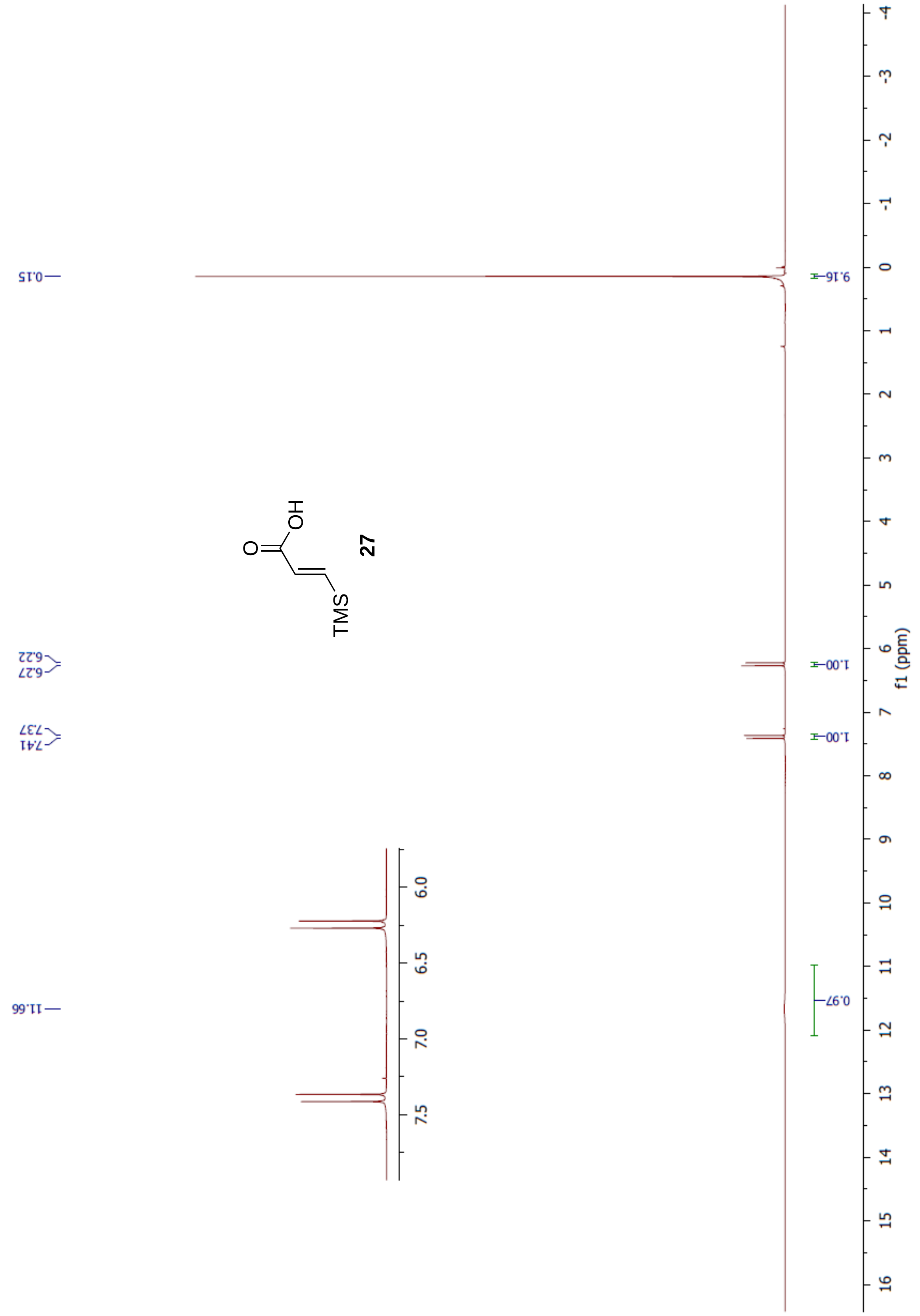

Figure S33: ^{1}H NMR spectrum of **27** in $CDCl_3$.

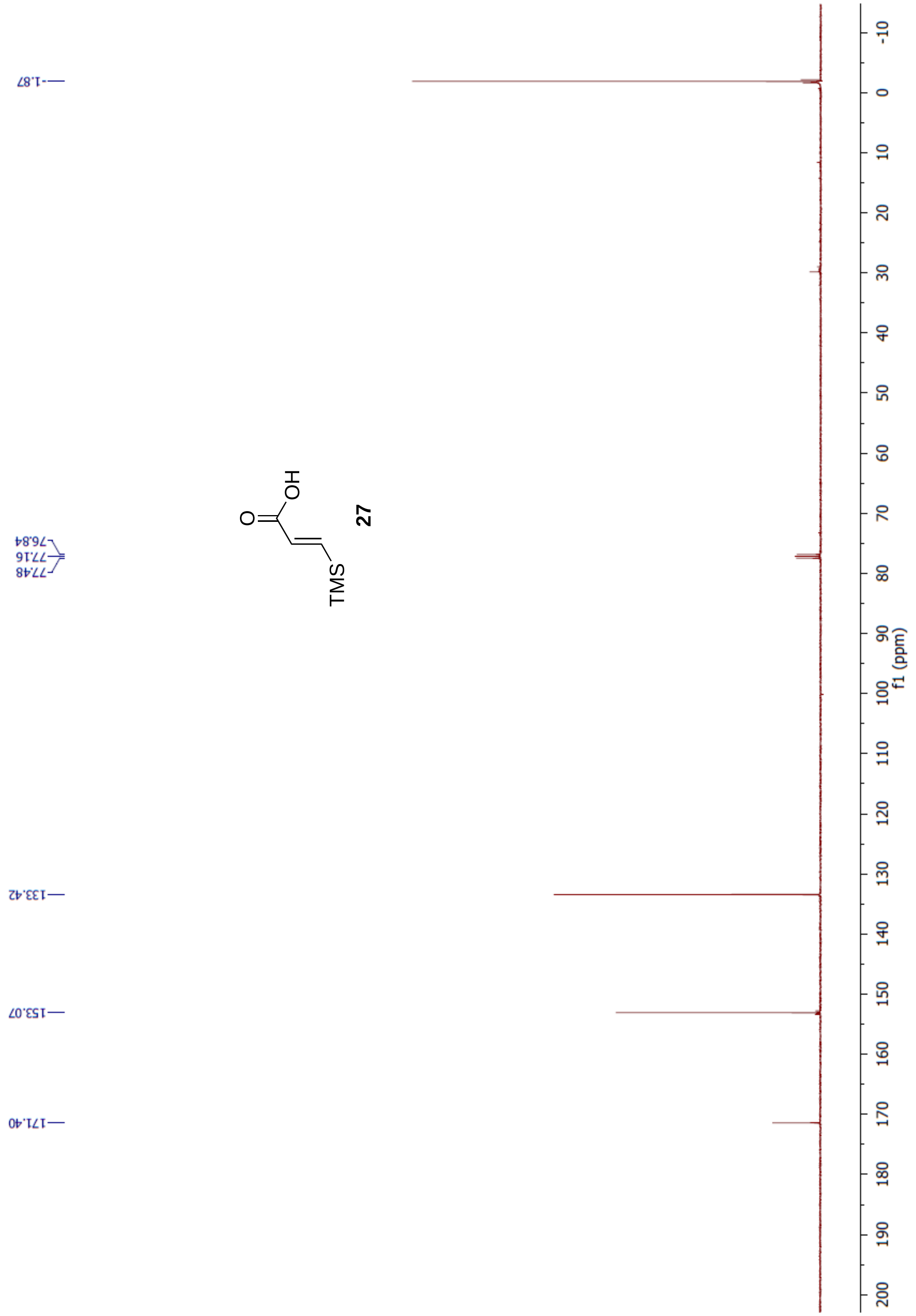

Figure S34: $^{13}C\{^{1}H\}$ NMR spectrum of **27** in $CDCl_3$.

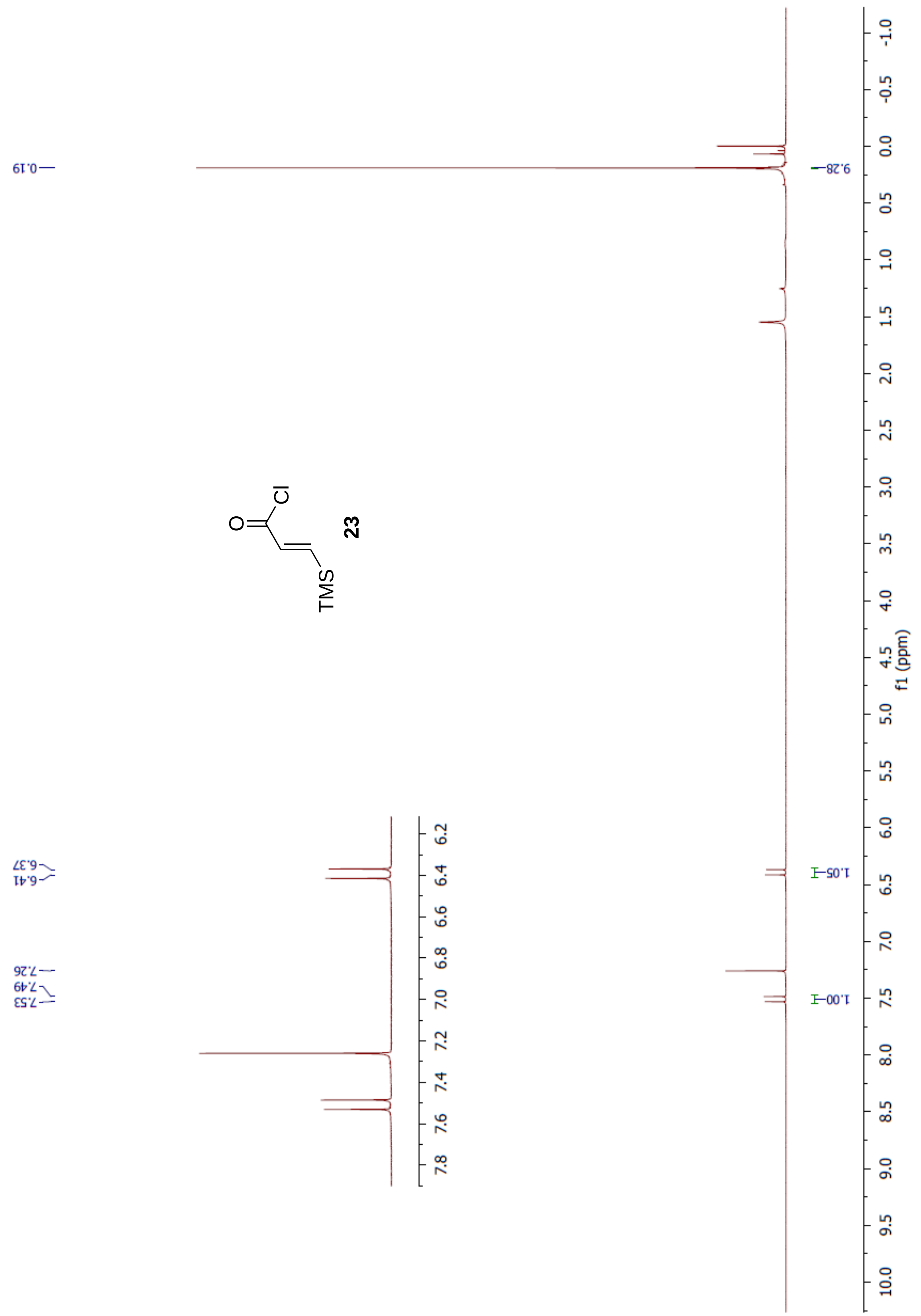

Figure S35: ^{1}H NMR spectrum of **23** in $CDCl_3$.

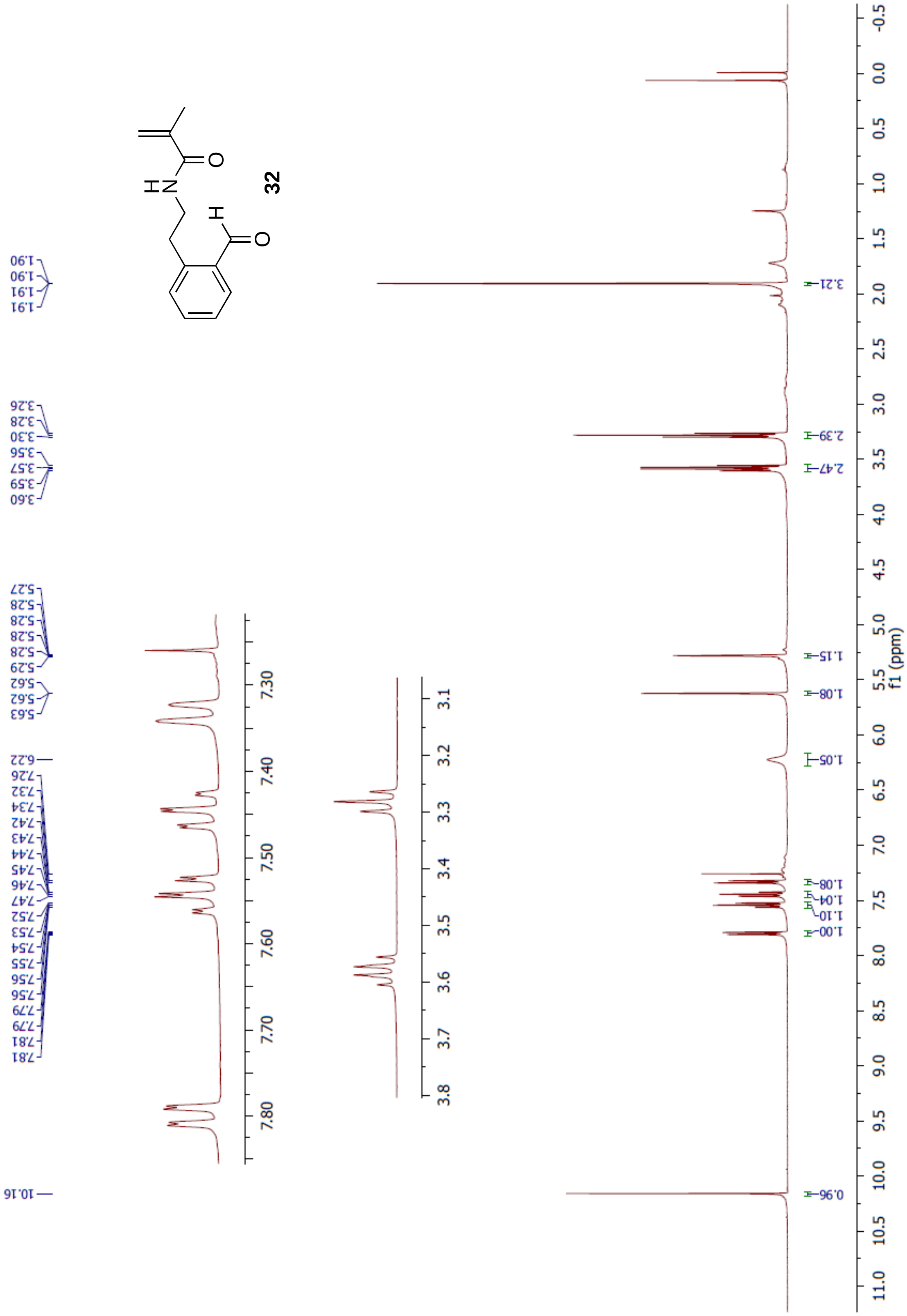

Figure S36: ^{1}H NMR spectrum of **32** in $CDCl_3$.

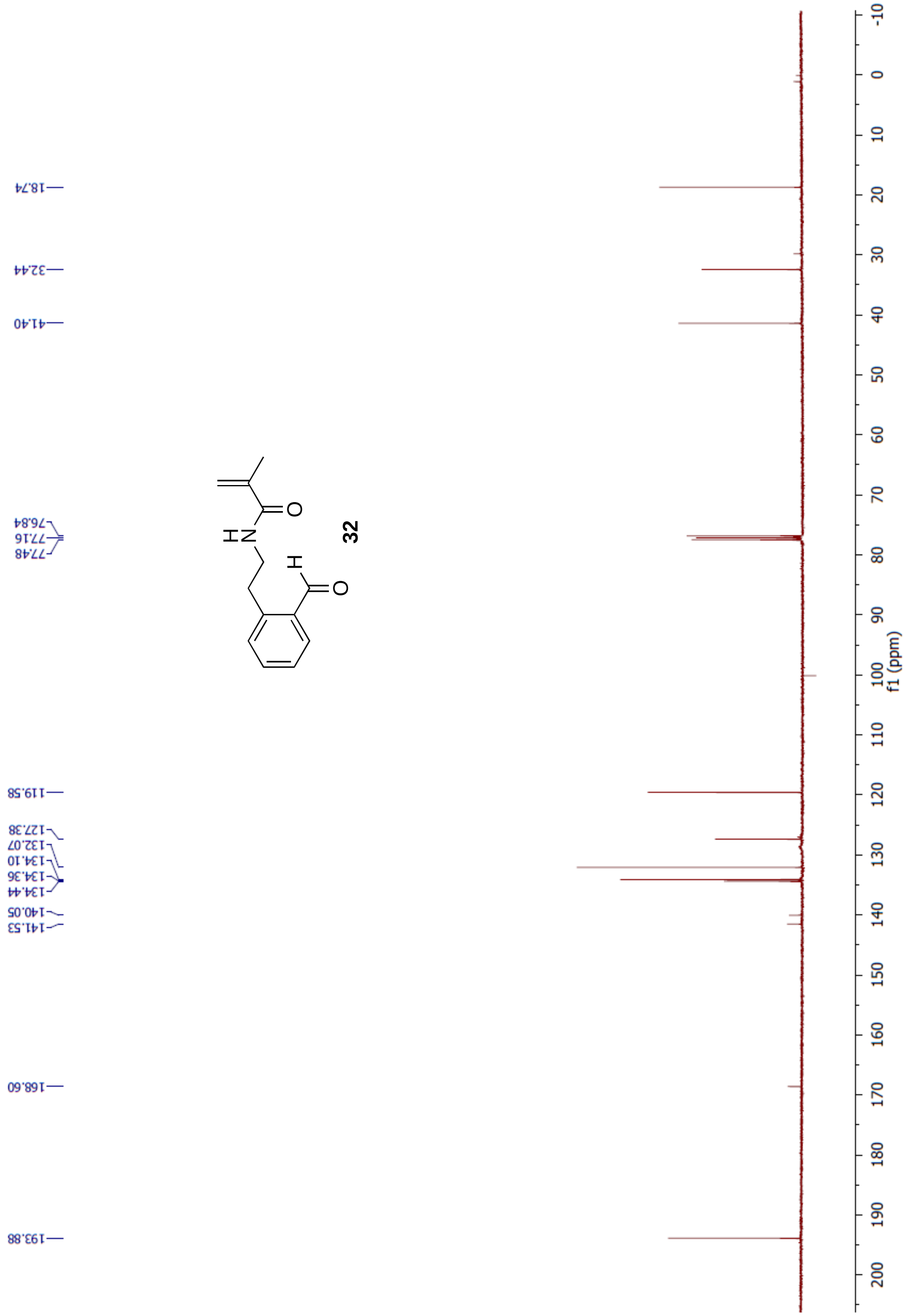

Figure S37: $^{13}C\{^{1}H\}$ NMR spectrum of **32** in $CDCl_3$.

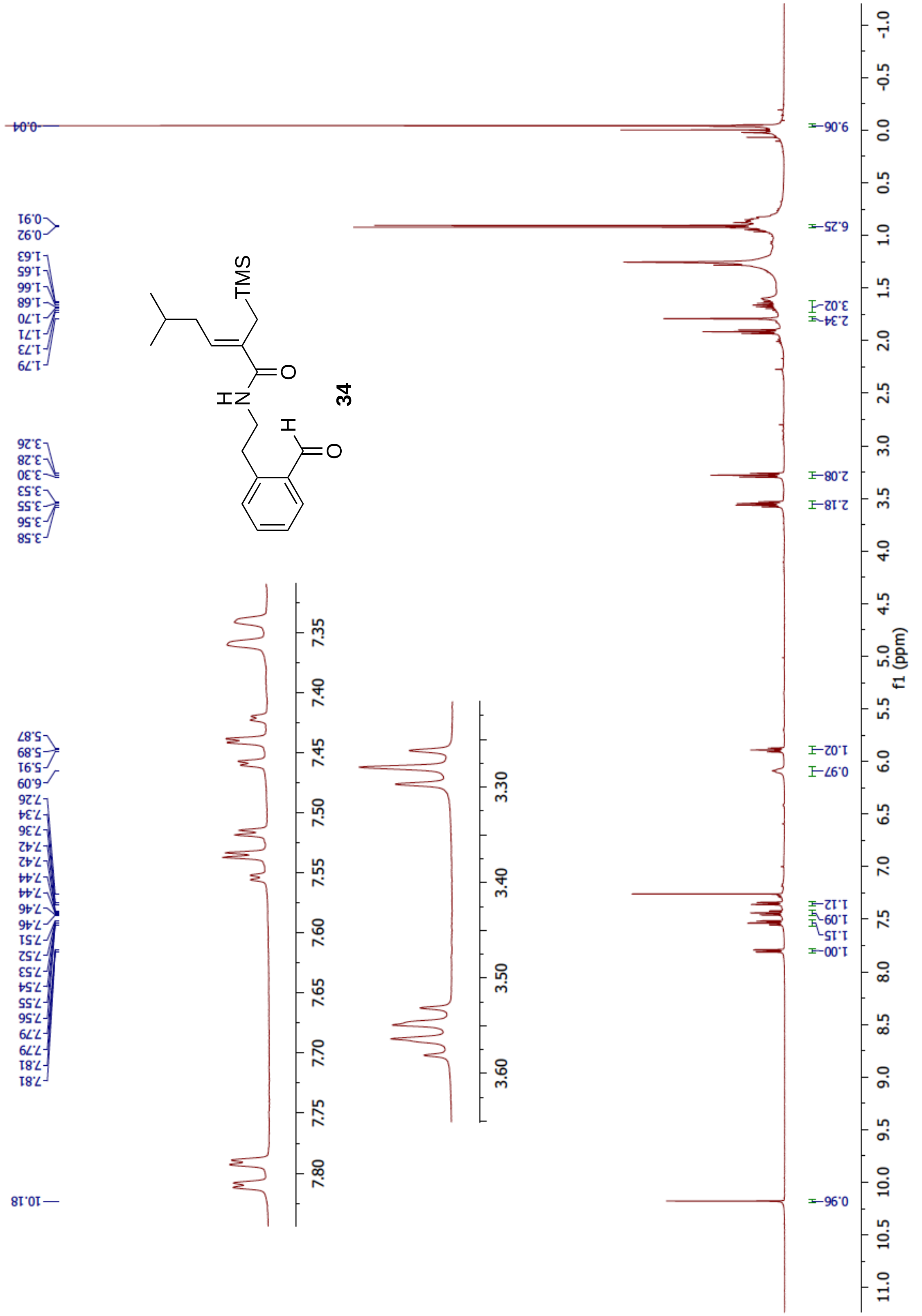

Figure S38: ^{1}H NMR spectrum of **34** in $CDCl_3$.

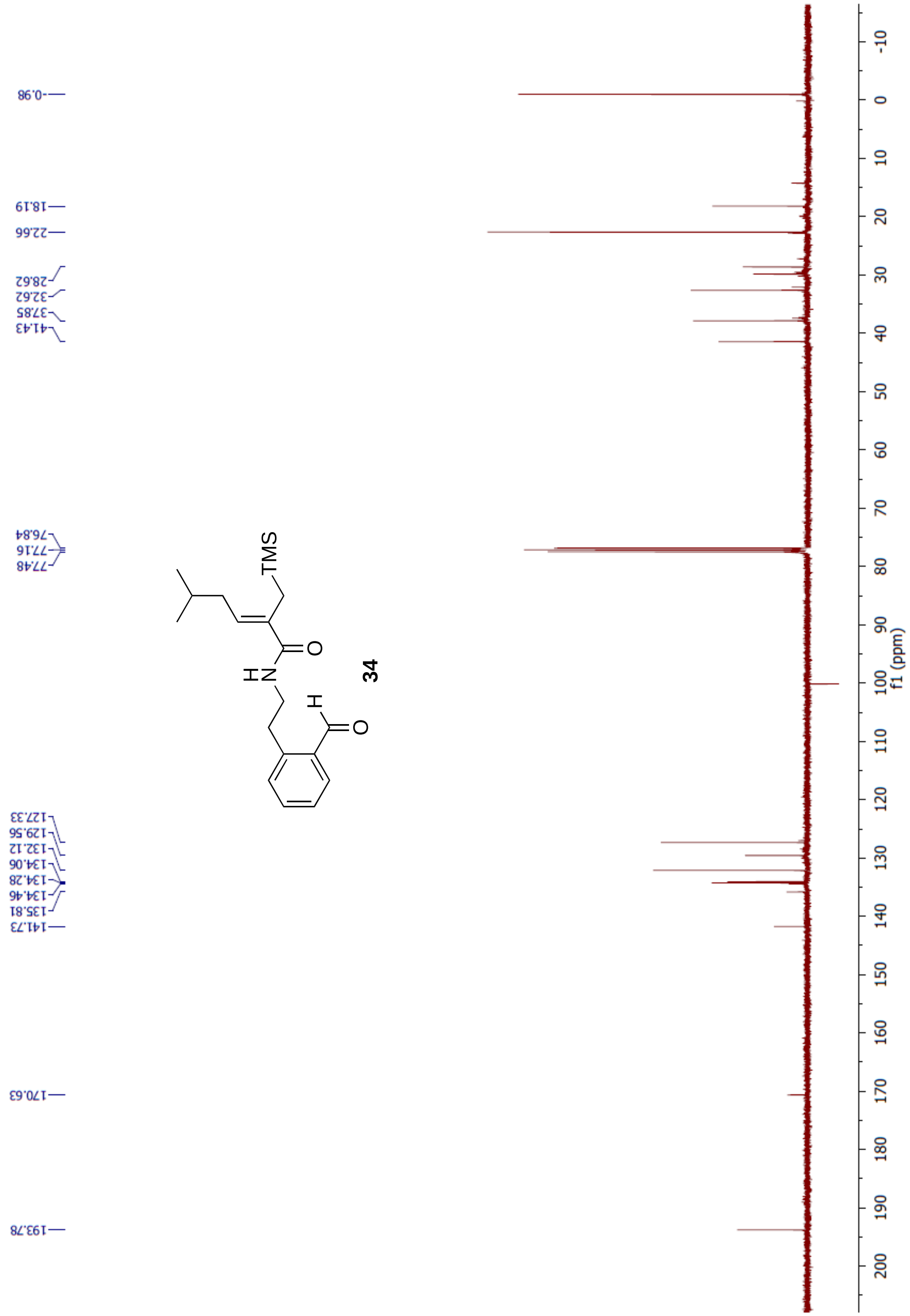

Figure S39: $^{13}C\{^{1}H\}$ NMR spectrum of **34** in $CDCl_3$.

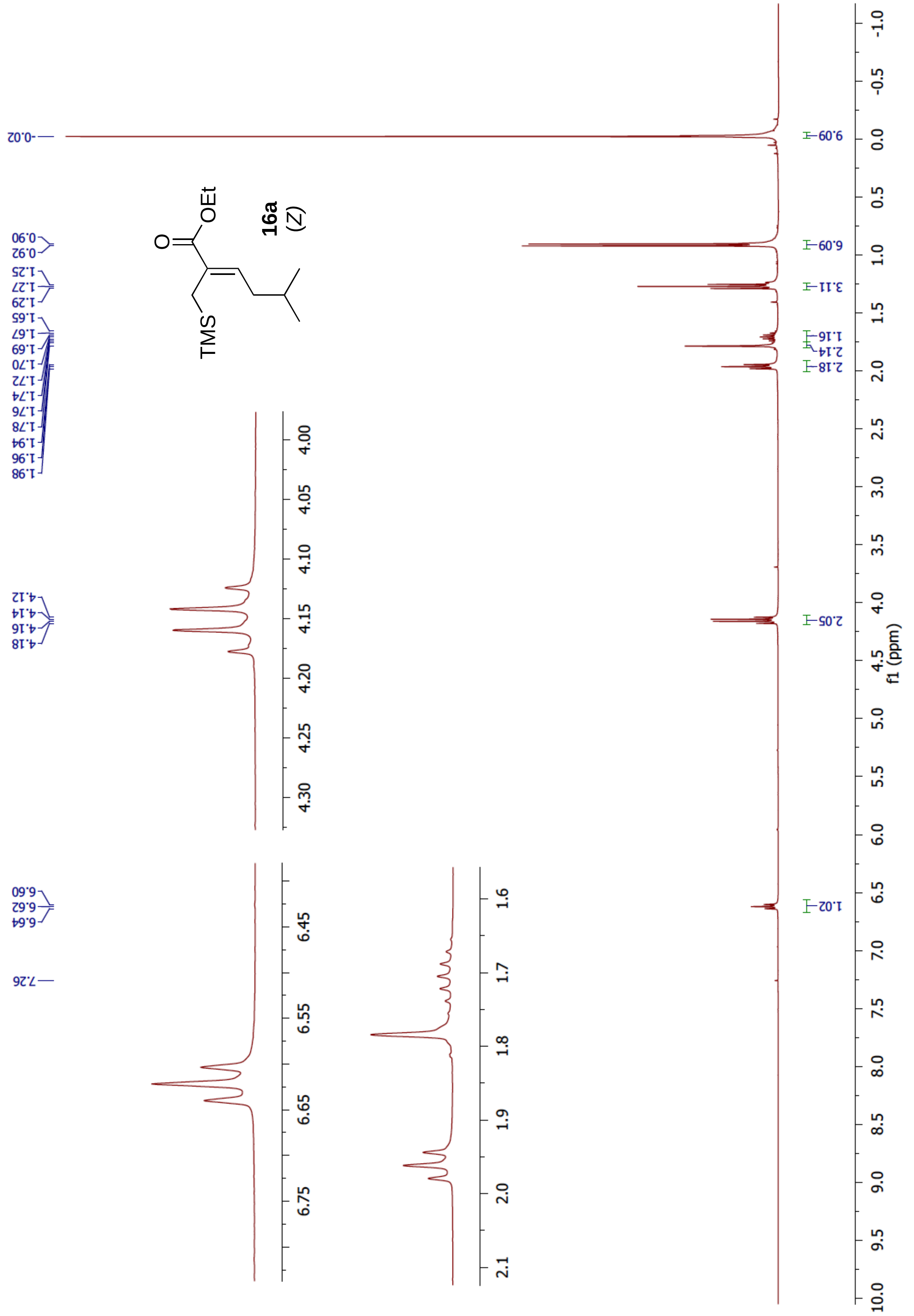

Figure S40: ^{1}H NMR spectrum of **16a** in $CDCl_3$.

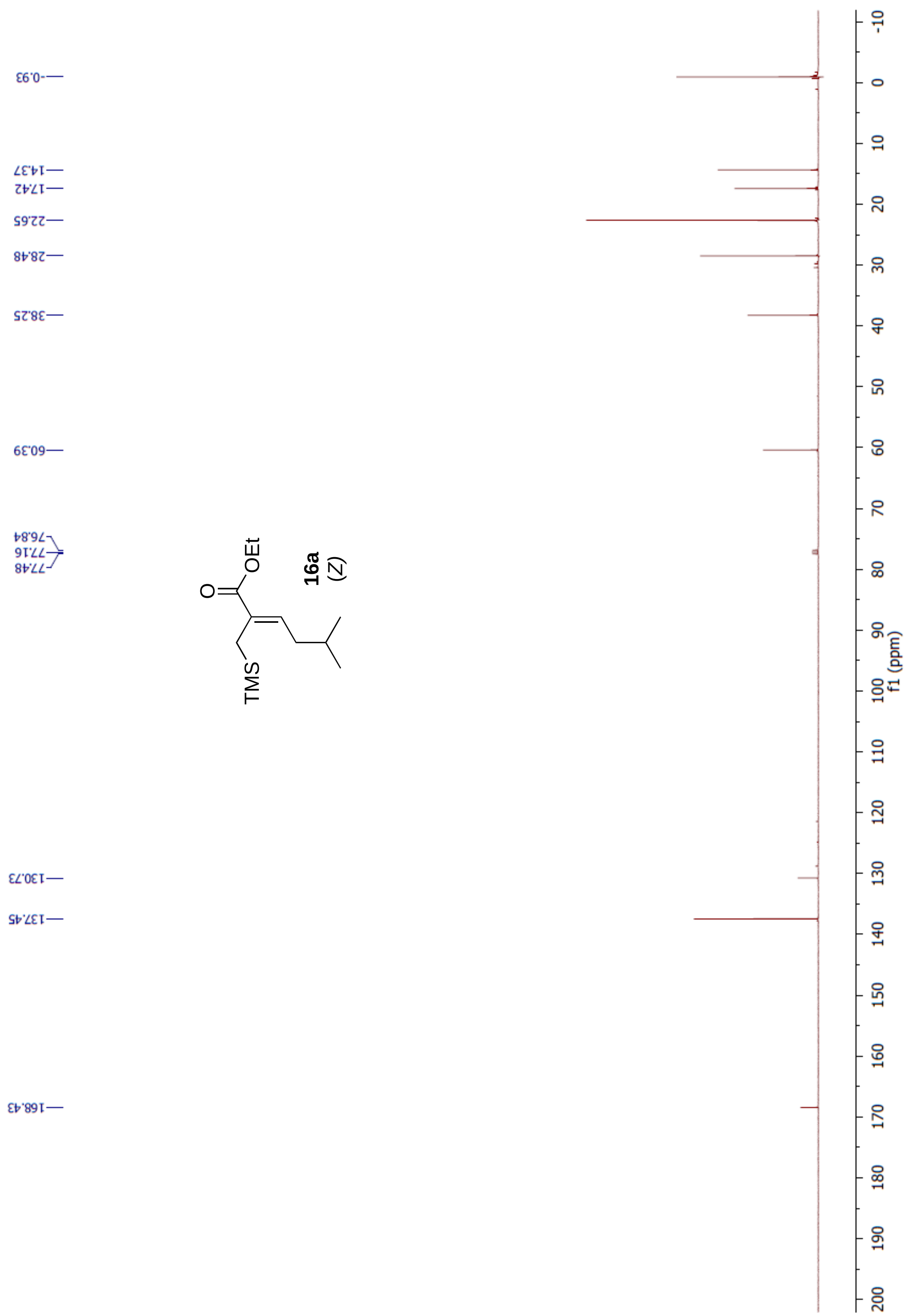

Figure S41: $^{13}C\{^1H\}$ NMR spectrum of **16a** in $CDCl_3$.

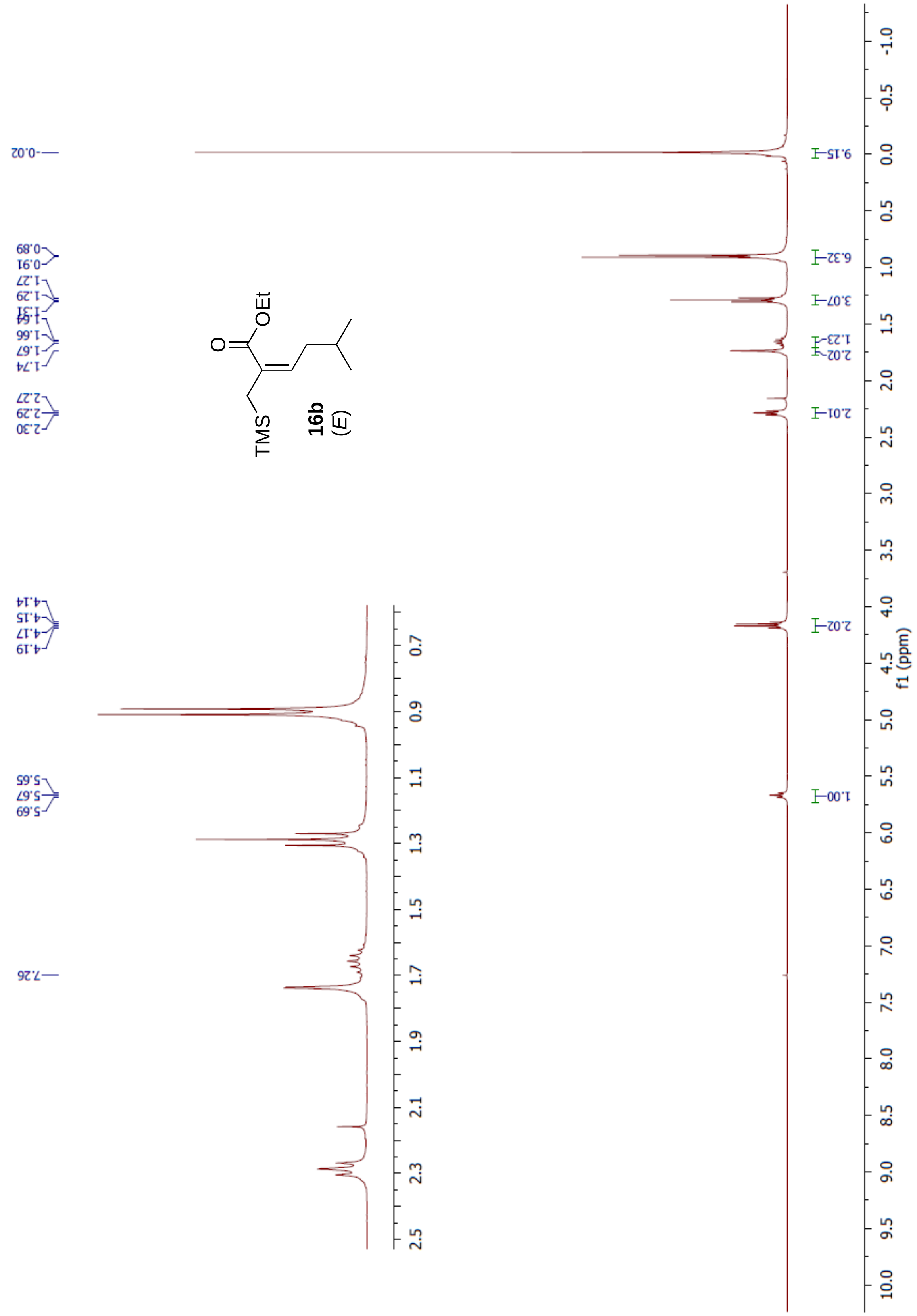

Figure S42: ^{1}H NMR spectrum of **16b** in $CDCl_3$.

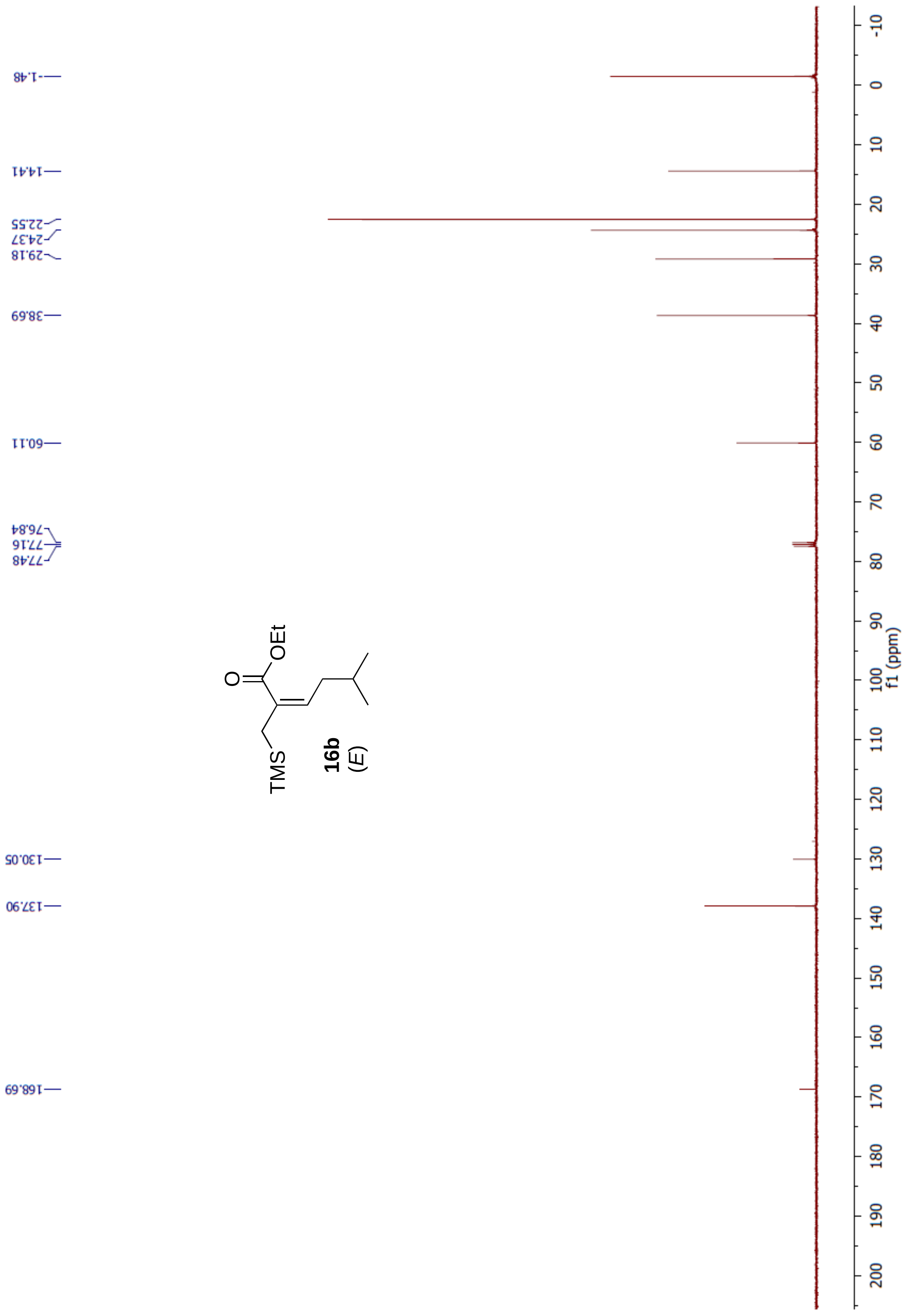

Figure S43: $^{13}C\{^{1}H\}$ NMR spectrum of **16b** in $CDCl_3$.

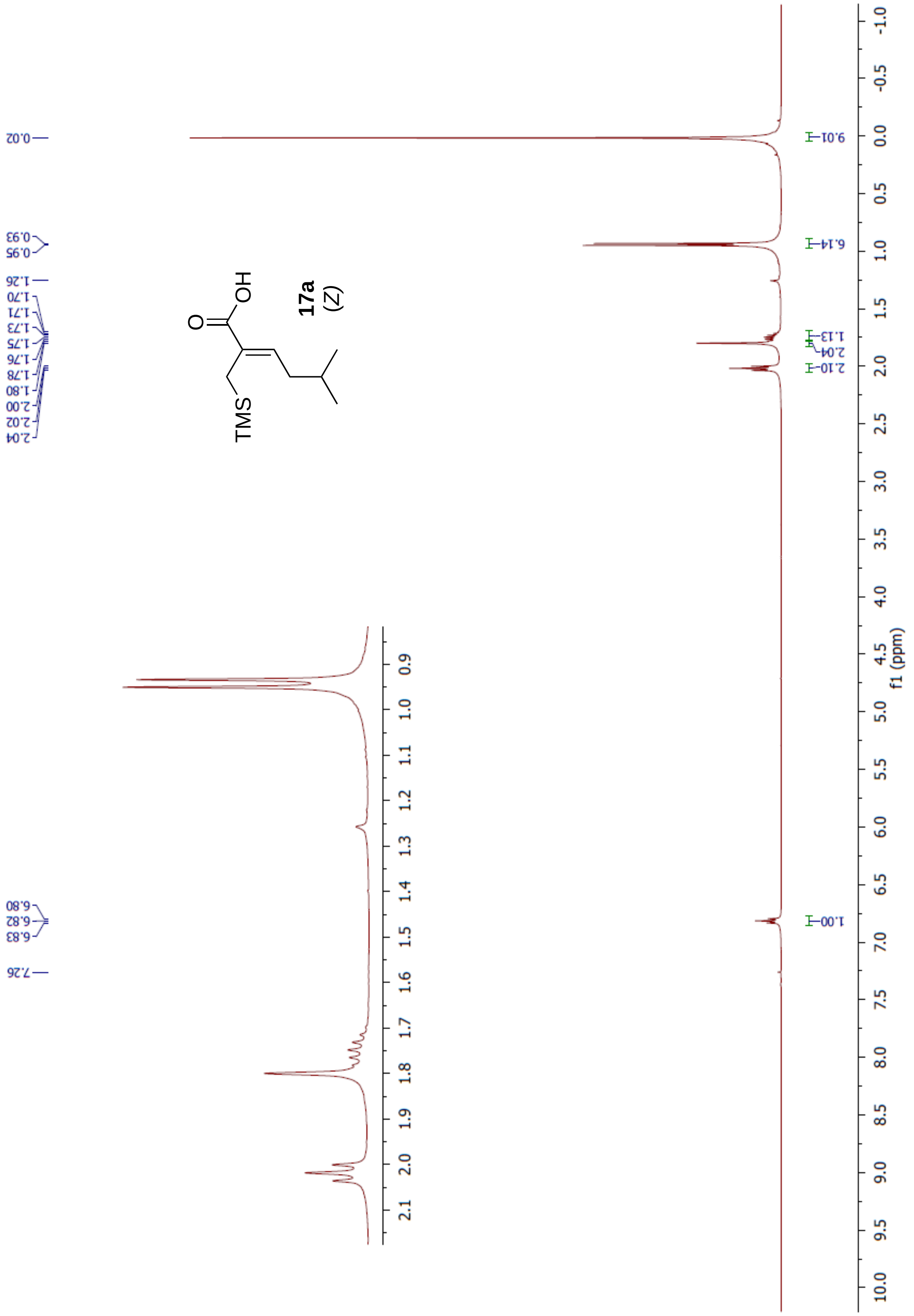

Figure S44: ^{1}H NMR spectrum of **17a** in $CDCl_3$.

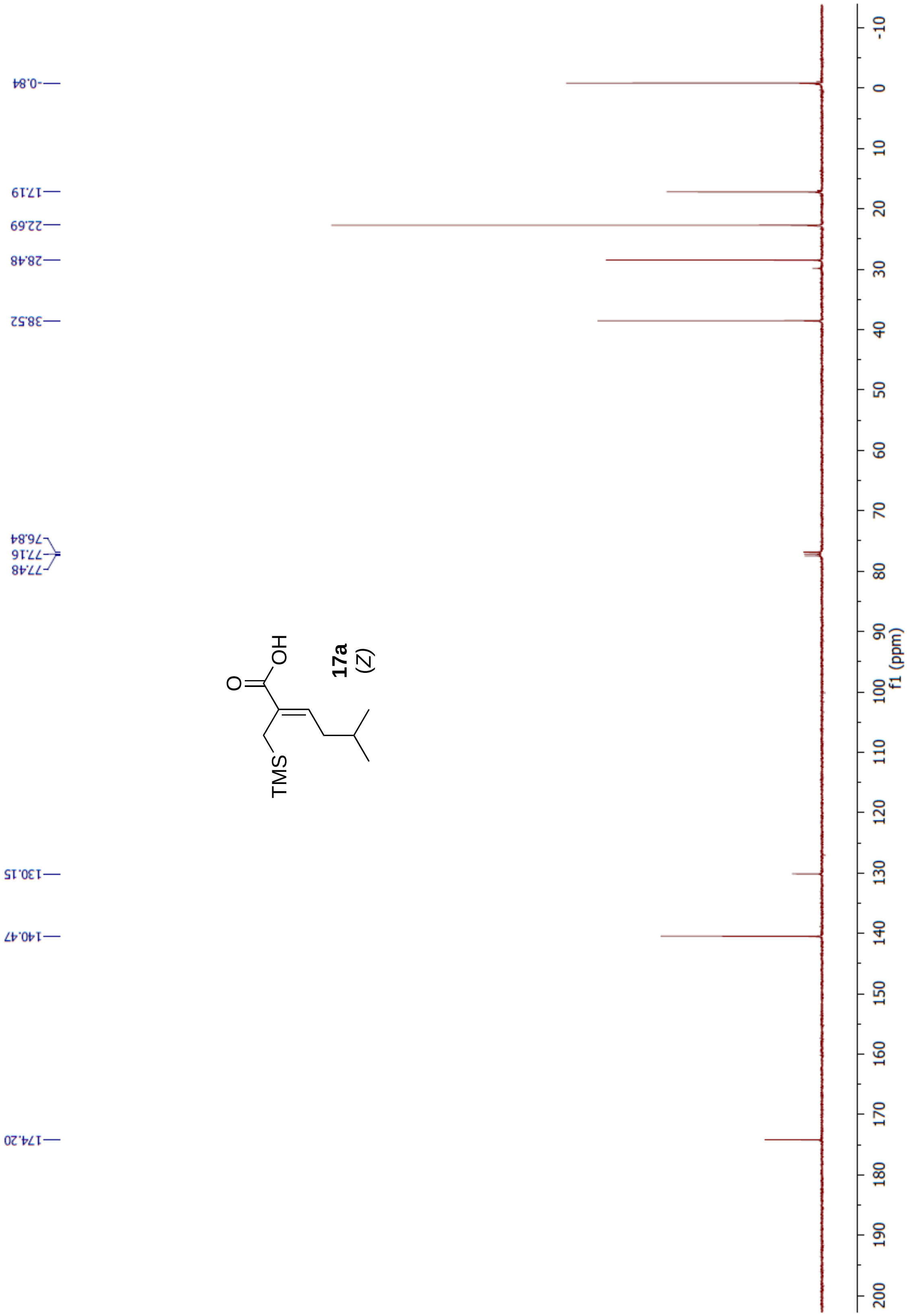

Figure S45: $^{13}C\{^1H\}$ NMR spectrum of **17a** in $CDCl_3$.

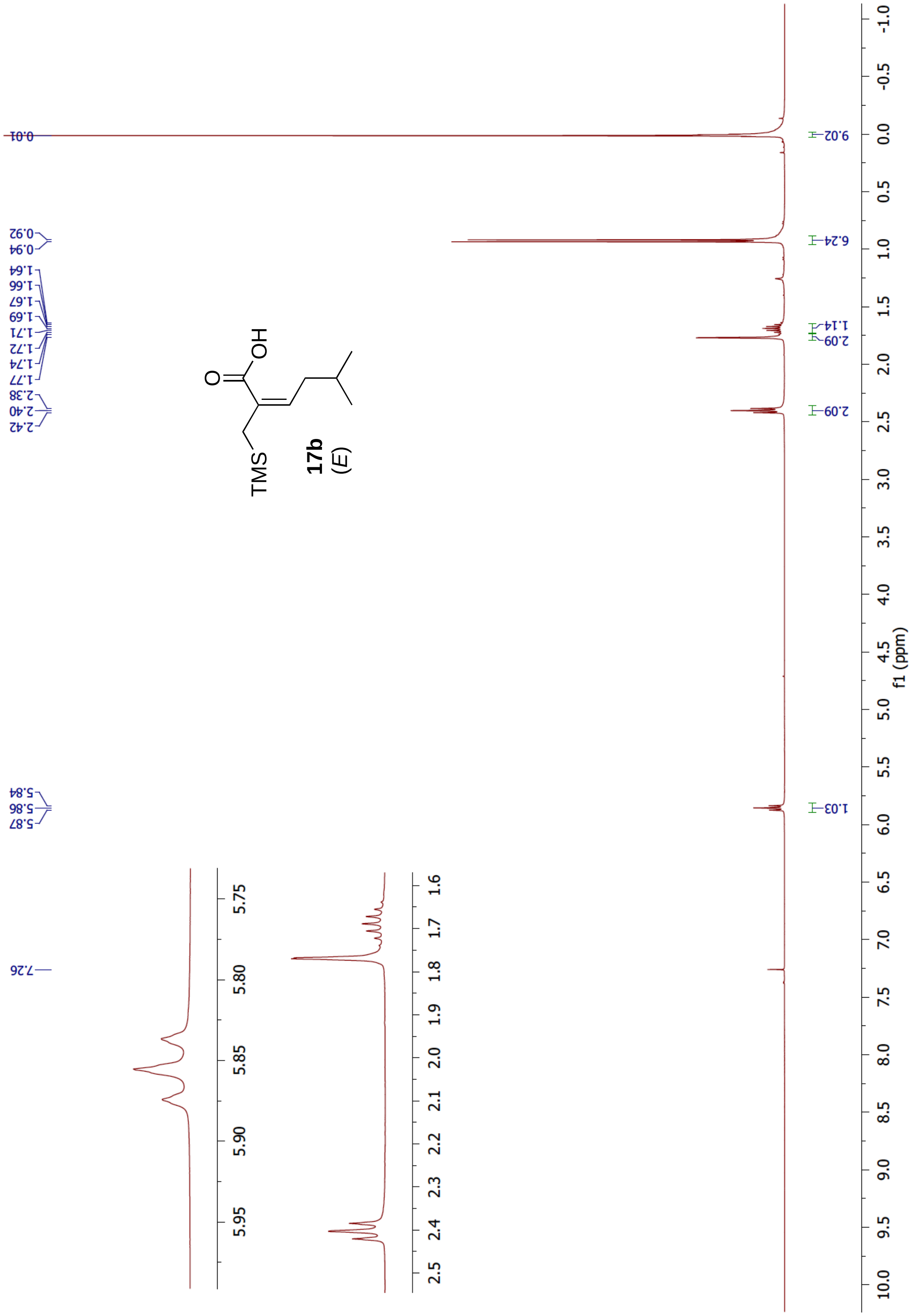

Figure S46: ^{1}H NMR spectrum of **17b** in $CDCl_3$.

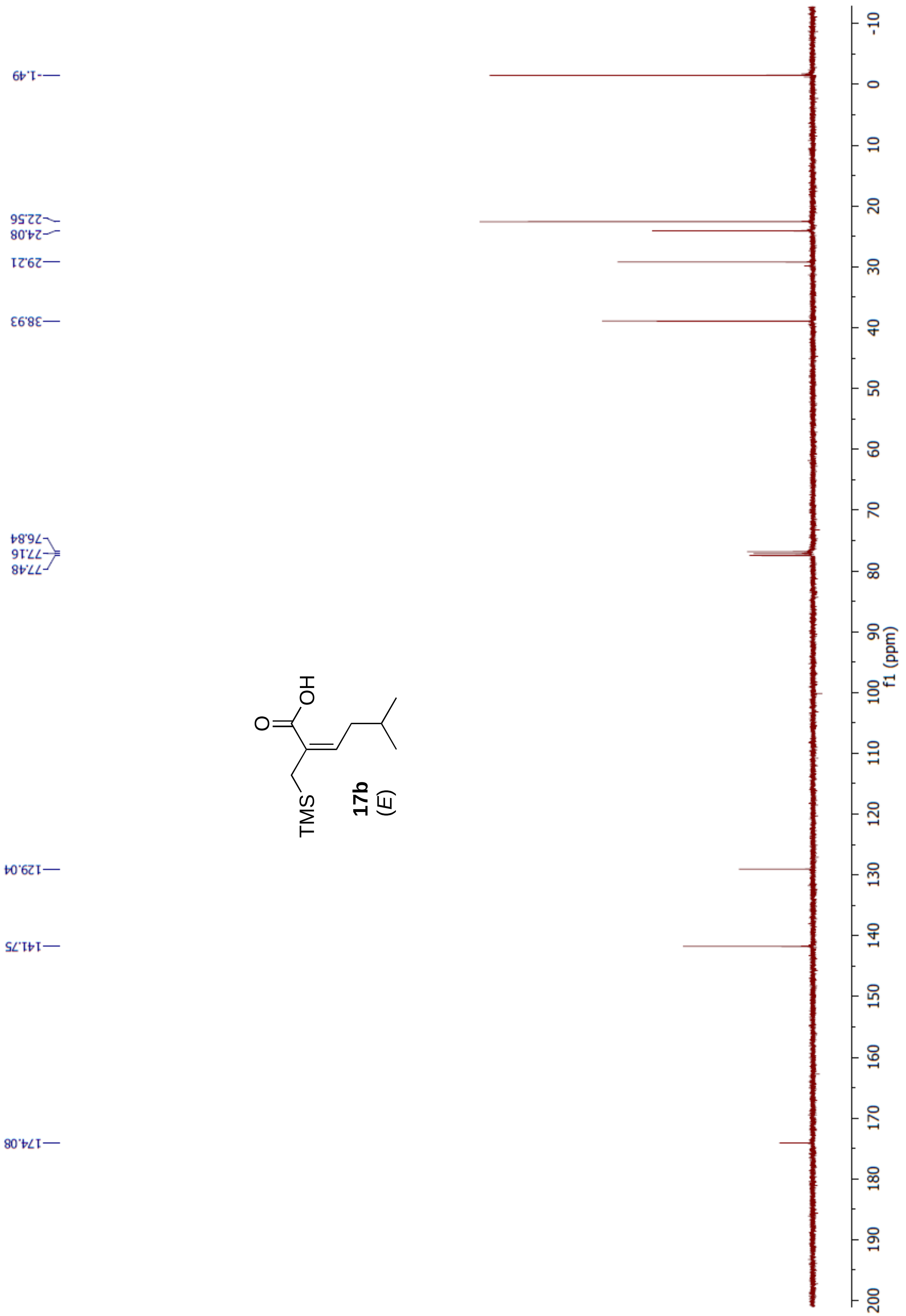

Figure S47: $^{13}C\{^{1}H\}$ NMR spectrum of **17b** in $CDCl_3$.

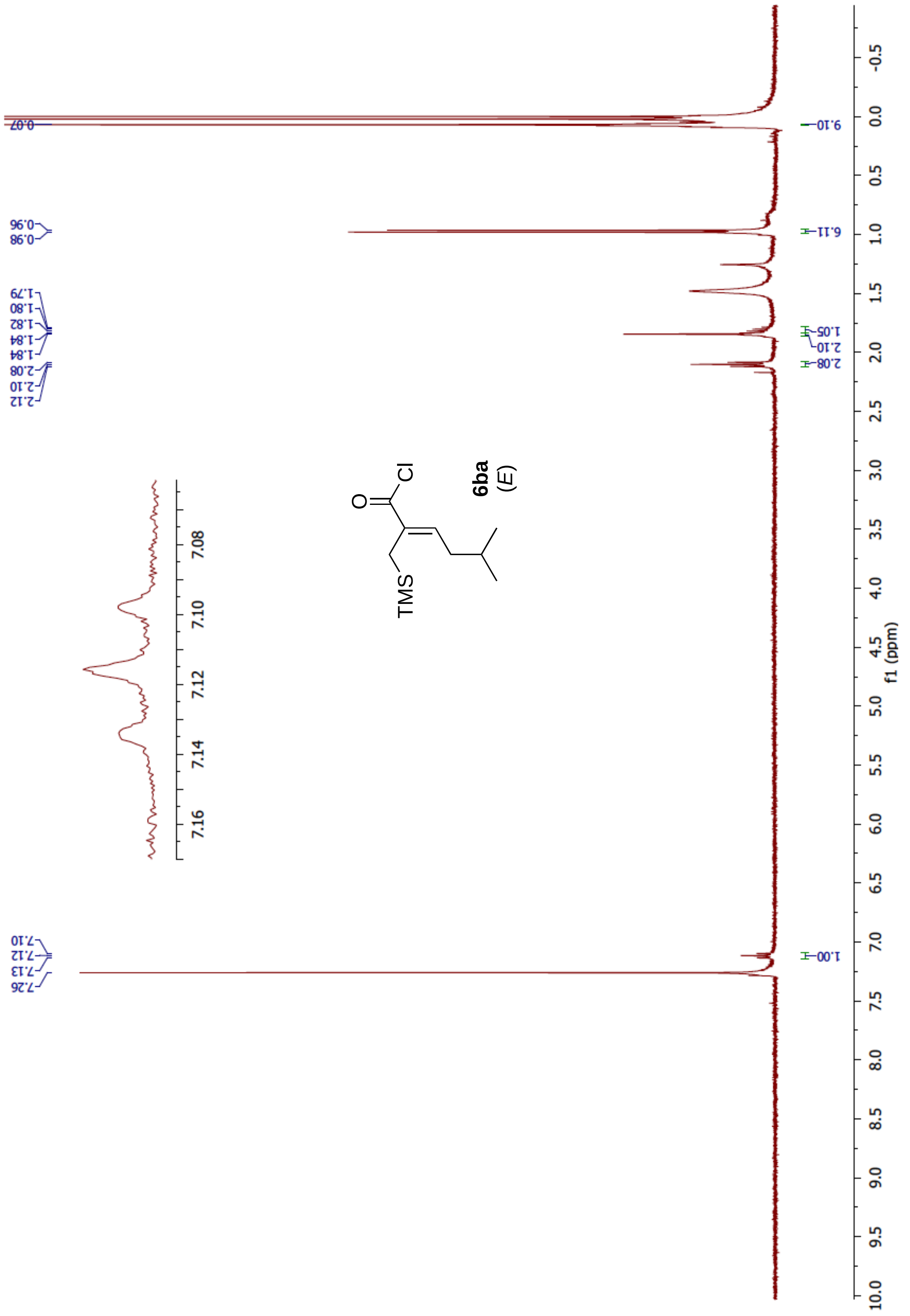

Figure S48: ^{1}H NMR spectrum of **6ba** in $CDCl_3$.

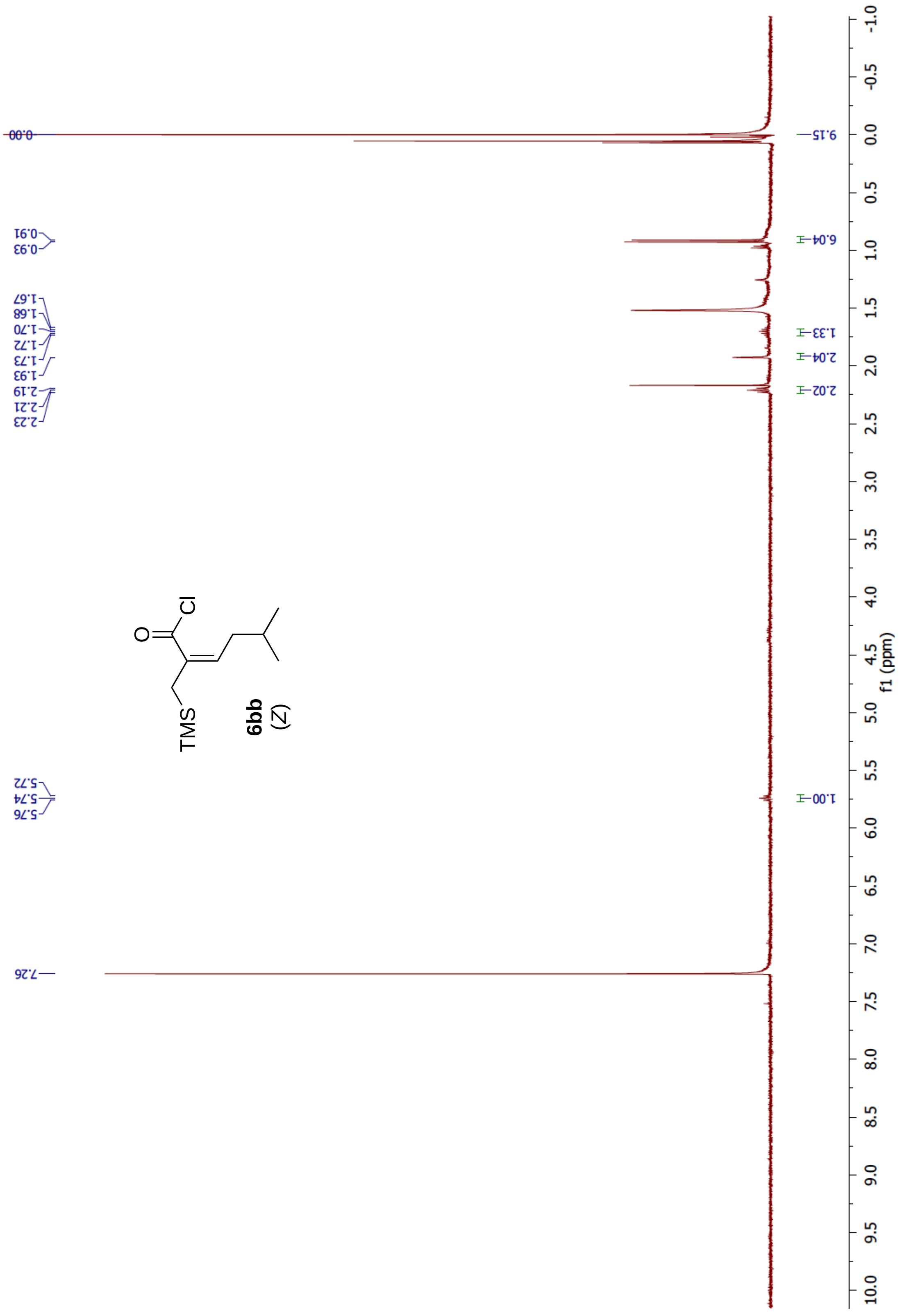

Figure S49: ^{1}H NMR spectrum of **6bb** in $CDCl_3$.
